# Therapeutic relevance of the protein phosphatase 2A in cancer

**DOI:** 10.18632/oncotarget.11399

**Published:** 2016-08-19

**Authors:** Chelsea E. Cunningham, Shuangshuang Li, Frederick S. Vizeacoumar, Kalpana Kalyanasundaram Bhanumathy, Joo Sang Lee, Sreejit Parameswaran, Levi Furber, Omar Abuhussein, James M. Paul, Megan McDonald, Shaina D. Templeton, Hersh Shukla, Amr M. El Zawily, Frederick Boyd, Nezeka Alli, Darrell D. Mousseau, Ron Geyer, Keith Bonham, Deborah H. Anderson, Jiong Yan, Li-Yuan Yu-Lee, Beth A. Weaver, Maruti Uppalapati, Eytan Ruppin, Anna Sablina, Andrew Freywald, Franco J. Vizeacoumar

**Affiliations:** ^1^ Department of Pathology, Cancer Cluster, College of Medicine, University of Saskatchewan, Saskatoon, Saskatchewan, S7N 5E5 Canada; ^2^ Center for Bioinformatics and Computational Biology, Department of Computer Science, University of Maryland, Maryland, MD 20742, USA; ^3^ College of Pharmacy, University of Saskatchewan, Saskatoon, Saskatchewan, S7N 2Z4, Canada; ^4^ Cell Signaling Laboratory, University of Saskatchewan, Saskatoon, Saskatchewan, S7N 5E5 Canada; ^5^ Cancer Research, Saskatchewan Cancer Agency, Saskatoon, Saskatchewan, S7N 5E5, Canada; ^6^ Department of Molecular and Cellular Biology, Baylor College of Medicine, Houston, TX 77030, USA; ^7^ Department of Cell and Regenerative Biology and Carbone Cancer Center, University of Wisconsin-Madison, Madison, WI 53705-2275, USA; ^8^ VIB Center for the Biology of Disease, VIB, 3000 Leuven, Belgium

**Keywords:** synthetic dosage lethality, PLK1, PP2A, chromosomal instability, mitotic checkpoint

## Abstract

Chromosomal Instability (CIN) is regarded as a unifying feature of heterogeneous tumor populations, driving intratumoral heterogeneity. Polo-Like Kinase 1 (PLK1), a serine-threonine kinase that is often overexpressed across multiple tumor types, is one of the key regulators of CIN and is considered as a potential therapeutic target. However, targeting PLK1 has remained a challenge due to the off-target effects caused by the inhibition of other members of the polo-like family. Here we use synthetic dosage lethality (SDL), where the overexpression of PLK1 is lethal only when another, normally non-lethal, mutation or deletion is present. Rather than directly inhibiting PLK1, we found that inhibition of PP2A causes selective lethality to PLK1-overexpressing breast, pancreatic, ovarian, glioblastoma, and prostate cancer cells. As PP2A is widely regarded as a tumor suppressor, we resorted to gene expression datasets from cancer patients to functionally dissect its therapeutic relevance. We identified two major classes of PP2A subunits that negatively correlated with each other. Interestingly, most mitotic regulators, including PLK1, exhibited SDL interactions with only one class of PP2A subunits (PPP2R1A, PPP2R2D, PPP2R3B, PPP2R5B and PPP2R5D). Validation studies and other functional cell-based assays showed that inhibition of PPP2R5D affects both levels of phospho-Rb as well as sister chromatid cohesion in PLK1-overexpressing cells. Finally, analysis of clinical data revealed that patients with high expression of mitotic regulators and low expression of Class I subunits of PP2A improved survival. Overall, these observations point to a context-dependent role of PP2A that warrants further exploration for therapeutic benefits.

## INTRODUCTION

Continued efforts in tumor sequencing have greatly facilitated the identification of the molecular alterations that provide novel opportunities to develop customized precision medicine [1]. However, the depth of molecular alterations observed within the malignancies that manifest as tumor heterogeneity represent a major roadblock, as the genetic diversity within a single tumor may lead to differential response of the tumors to targeted therapies and subsequent treatment failure [2, 3]. Genome instability is one of the key driving forces of tumor heterogeneity, ensuring that no two tumors are exactly alike and that no single tumor is composed of genetically identical cells [4, 5]. Chromosomal INstability (CIN) is a type of genome instability that is observed in up to 50% of human cancers [6–8]. As CIN is a common feature of cancer cells, therapeutic strategies targeting CIN could limit treatment failure and have the potential to overcome drug resistance.

Mitotic checkpoint components are frequently overexpressed and are known to induce CIN [9–15], and so targeting the mitotic checkpoint or its associated components is conceived as a potential avenue to overcome CIN. However, decreasing the activity of mitotic checkpoint components also promotes chromosome missegregation and CIN. Therefore, alternate strategies to mitigate tumor heterogeneity without directly inhibiting mitotic components is expected to benefit cancer patients. A recent elegant study [16], took advantage of an approach called synthetic dosage lethality (SDL) [17], where the overexpression of one gene caused lethality only when another non lethal gene was deleted. This study identified protein phosphatase 2A (PP2A) as a therapeutic target in cells overexpressing the checkpoint protein MAD2 [16]. Since MAD2 overexpression is known to induce CIN [16, 18–21], this lethal genetic interaction between MAD2 and PP2A, could lend to the selective killing of CIN cells. Interestingly, inhibition of some of the PP2A subunits has been reported to impair the high-fidelity homologous recombination repair pathway and sensitized cells to either PARP inhibitors [22] or to radiation therapy [23]. In addition, several subunits of PP2A have also been described to interact with mitotic regulators, including the serine-threonine Polo-like kinase 1 (PLK1), which is essential for spindle pole separation and has recently been implicated in the mitotic checkpoint [24]. These studies suggest a role for the PP2A components in the maintenance of genome stability [22, 23, 25–27]. These results also suggest that targeting of specific PP2A complexes might lead to selective lethality of CIN cells and perhaps alleviate tumor heterogeneity related issues.

Controversially, PP2A is widely recognized as a tumor suppressor and has been shown to play a role in metabolism, cell cycle and mitotic progression, DNA replication, gene expression and translation, signal transduction, proliferation, and apoptosis [28–30]. PP2A is a heterotrimeric enzyme, consisting of a catalytic, scaffolding, and regulatory subunit. There are two genes coding the catalytic subunit, two genes coding the scaffolding subunit, and twelve distinct regulatory subunits (Supplementary Table S1). This allows formation of more than 70 different PP2A holoenzymes [30]. The regulatory subunits provide specificity to the substrate interactions and allow the PP2A complex to selectively regulate signaling pathways [31].

While the activity of protein phosphatases or kinases can be fine-tuned to alter cellular signaling for therapeutic benefits, we hypothesized that the dissection of the functional and clinical relevance of PP2A complexes, across multiple cancer types, might provide an opportunity to overcome tumor heterogeneity. Here we show a specific subset of PP2A subunits to exhibit SDL interactions with several mitotic proteins, and inhibition of these proteins could be effectively employed to mitigate tumor heterogeneity.

## RESULTS

### PP2A inhibition impairs the growth of PLK1 and MAD1-overexpressing cells

**Figure 1:**
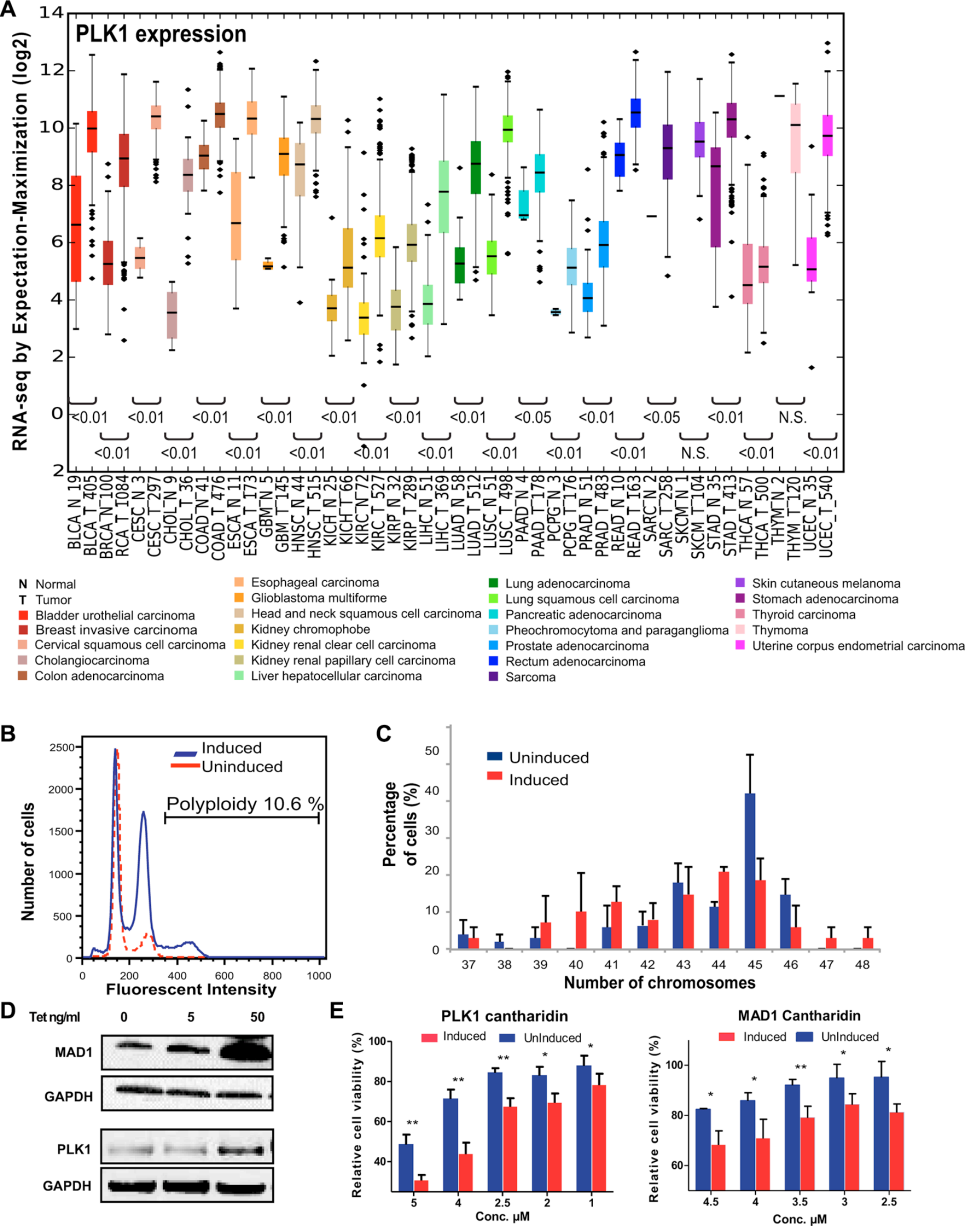
Mitotic regulators are frequently overexpressed in many tumor types, cause aneuploidy and confer sensitivity to PP2A-inhibitor mediated cell death (**A**) Expression scores for PLK1 within 24 different types of cancer and normal tissue from TCGA. The numbers in x-axis labels denotes the number of patient samples in each cancer type. Statistical significance of the difference in expression between the normal and tumor samples are depicted for each cancer type. N.S. denotes not significant. The abbreviation of each cancer in the axis label is represented as described in the TCGA portal. (**B**) Histogram of the DNA content in uninduced and induced HCT116-PLK1 cells as analyzed by flow cytometry. (**C**) Quantification of the number of chromosomes in HCT116-PLK1 uninduced and induced cells as seen in metaphase-spread analysis after 24 hours of induction. *N* = 12 to 20 cells per condition with Mean ± SD from three independent experiments represented. (**D**) Western blot analysis of the inducible DLD1-MAD1 and HCT116-PLK1 cells showing increased protein expression with increasing concentrations of TET. (**E**) Bar graphs displaying cell survival as measured by resazurin assay relative to a DMSO-treated control of each inducible cell line treated with varying concentrations of cantharidin for 96 hours for the uninduced and induced populations. *N* = 3 with Mean ± SD from three independent experiments represented. *p < 0.05; **p < 0.005.

Using the Cancer Genome Atlas (TCGA) (https://tcga-data.nci.nih.gov/tcga/) database, we determined if expression of mitotic genes in cancer patients are differentially regulated between normal tissues and the tumor tissues. We found several mitotic regulators, including PLK1, exhibited significant higher mRNA expression, compared to normal tissue, across 24 different types of cancers (Figure 1A and Supplementary Figure S1A). This indicated that overexpression of mitotic regulators is a frequent occurrence and could play an important role in tumor progression in many cancer types. Some of these mitotic regulators have been shown to induce frequent gain or loss of chromosomes leading to heterogeneity [9, 18, 19]. The recent identification of the SDL interaction between the mitotic checkpoint protein MAD2 and PPP2R1A provides a possible avenue to overcome tumor heterogeneity [16]. As most of the mitotic regulators, including PLK1, function coherently during mitosis, we tested the generalization of this SDL interaction with two mitotic components, MAD1 and PLK1. We conducted these analyses using an inducible system that allows tetracycline-dependent expression of PLK1 or MAD1 in chromosomally stable HCT116 or DLD1 cell lines, respectively [9, 32]. These cells have intact DNA damage and mitotic checkpoints and therefore do not inherently exhibit CIN [33]. While MAD1 overexpression has recently been shown to induce CIN [9], a constitutively active form of PLK1 (S137D) was used in the HCT116 cells, as expression of PLK1-S137D has been known to cause spindle assembly checkpoint failure [34]. In addition, S137D ensures elevated kinase activity rather than simply overexpressing PLK1 protein [35]. However, prior to testing the SDL interactions between PLK1 and PP2A, we assessed the efficiency of PLK1-S137D to induce CIN. We found PLK1 induction led to aneuploidy, while the control HCT116 cells were predominantly diploid (Figure 1B). In particular, induction of PLK1 in HCT116 cells for 24 hrs followed by non-inducible media for the next 24hrs led to an increase in the aneuploidy population. However, when we constitutively induced PLK1 expression for several generations, the aneuploidy decreased over time (Supplementary Figure S2A), indicating that aneuploidy triggered by constitutive PLK1 upregulation could lead to cell death. Nevertheless, analysis of the number of chromosomes per cell in both the uninduced and constitutively induced populations identified frequent gain and loss of small numbers of chromosomes in PLK1-overexpressing cells (Figure 1C) indicating that PLK1 overexpression gives rise to heterogeneous population of cells. To test the SDL interaction between PLK1 and PP2A, we chose to use a constitutive induction strategy. This strategy was best suited to support our aim, which was to use PLK1 overexpression as the vulnerability associated with cancer cells and to test if these PLK1-overexpressing, heterogeneous population of cells exhibit SDL with PP2A inhibition. To confirm the SDL interactions between PP2A and the mitotic components, we utilized chemical-genetic modeling with a PP2A inhibitor, cantharidin. Cantharidin, a small molecule inhibitor of PP2A, has been shown to have higher affinity for PP2A than PP1 [36]. Increased cantharidin sensitivity was observed in TET-induced PLK1 and MAD1-overexpressing clonal populations compared to the uninduced population that does not overexpress these mitotic checkpoint proteins (Figure 1D, 1E). These results indicated that inhibition of PP2A provides a unique opportunity to eliminate CIN cells, induced by the overexpression of PLK1 or MAD1.

### Translation of the PP2A-PLK1 SDL interaction to cancer cells that naturally overexpress PLK1

**Figure 2:**
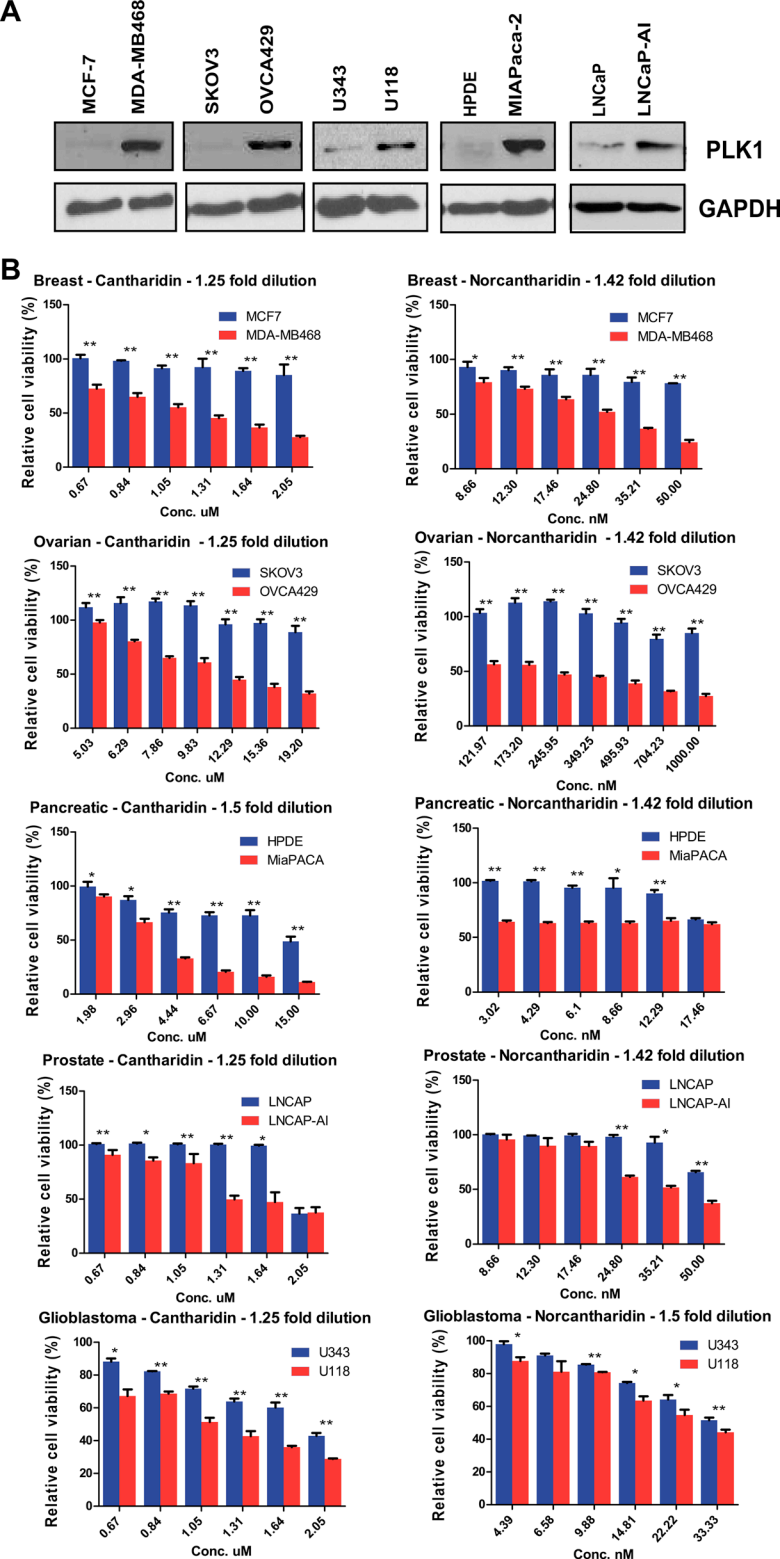
PP2A inhibition induces death in cells that naturally overexpress PLK1 (**A**) Western blot analysis of PLK1 expression in MCF7 and MDA-MB-468 breast cancer cells, HPDE and MiaPaCa-2 pancreatic cancer cells, SKOV3 and OVCA429 ovarian cancer cells, U343 and U118 glioblastoma cells, and LNCaP and LNCaP-AI prostate cancer cell lines. GAPDH is used as a loading control. (**B**) Bar graphs displaying the cell survival measured by resazurin assay relative to DMSO-treated ovarian, breast, glioblastoma, prostate and pancreatic cells treated with varying concentrations of cantharidin and norcantharidin for 72 hours. PLK1-overexpressing cells are shown in red and cell lines not overexpressing PLK1 are shown in blue. *N* = 3 with 8 replicates in each independent experiment. Mean ± SD from one independent experiment is represented. *p < 0.05; **p < 0.005.

PLK1 is overexpressed in colorectal, breast, pancreatic, ovarian, glioblastoma and prostate cancer cells [37–44]. It remains to be seen whether the SDL interactions between PP2A and PLK1 can be translated to PLK1-overexpressing tumors, regardless of the tissue type. As overexpression of PLK1 provides an opportunity to selectively kill CIN cells, we used the literature [38, 40] as well as gene expression analysis of multiple cell lines from the Cancer Cell Line Encyclopedia (CCLE) database (http://www.broadinstitute.org/ccle/home) to identify multiple non-isogenic pairs of cell lines across different tumor types, such that one cell line naturally overexpressing PLK1 could be compared to one that does not (Supplementary Figure S2B). Cell lines such as MDA-MB-468 have a genetic dependency on PLK1 [40], making it an excellent model to test the generalization of the SDL interaction. Similarly, we chose to test the pancreatic cell line MiaPaCa-2, as it has been reported to overexpress PLK1 ~60 fold compared to non-malignant HPDE cells [38]. After confirming PLK1 expression in the selected models, we tested their response to PP2A inhibition (Figure 2A).

Upon PP2A inhibition with cantharidin treatment, we found preferential loss in viability of the PLK1-overexpressing cells but not the control cells (Figure 2B). To corroborate the specificity of these results, a less toxic, de-methylated analog of cantharidin called nor-cantharidin [45] was also used. This small molecule also selectively inhibited PLK1-overexpressing cells (Figure 2B). The chemical genetic approach allowed us to validate the SDL interaction across multiple cell types. Similar results were obtained in other non-isogenic pairs of ovarian cancer and glioblastoma cell lines (Figure 2B). We also examined the effect of these small molecules in an isogenic pair of prostate cancer cells (LNCaP), one of which was derived after long-term androgen deprivation [46]. Since the expression of PLK1 is up regulated in the androgen insensitive LNCaP cells (LNCaP-AI) [37], we first confirmed the expression of PLK1 in the prostate cancer cells and then examined the sensitivity to both cantharidin and nor-cantharidin (Figure 2B). The LNCaP-AI cells were found to be more sensitive to treatment with the PP2A inhibitor (Figure 2B). While PLK1 expression was used to select cantharidin-responsive and non-responsive cells, we also analyzed expression of PLK1 in cell lines that were previously classified as either cantharidin-resistant or sensitive [36]. Gene expression data from CCLE database indicated that unlike resistant cells, cantharidin-sensitive cell lines had high expression of PLK1 (Supplementary Figure S2C). These results strongly indicate that PLK1-overexpression offers selective vulnerability to PP2A inhibition, regardless of tissue-type.

### Inhibition of PP2A by cantharidin causes mitotic defects and potentiates cell death in PLK1-overexpressing cells

**Figure 3:**
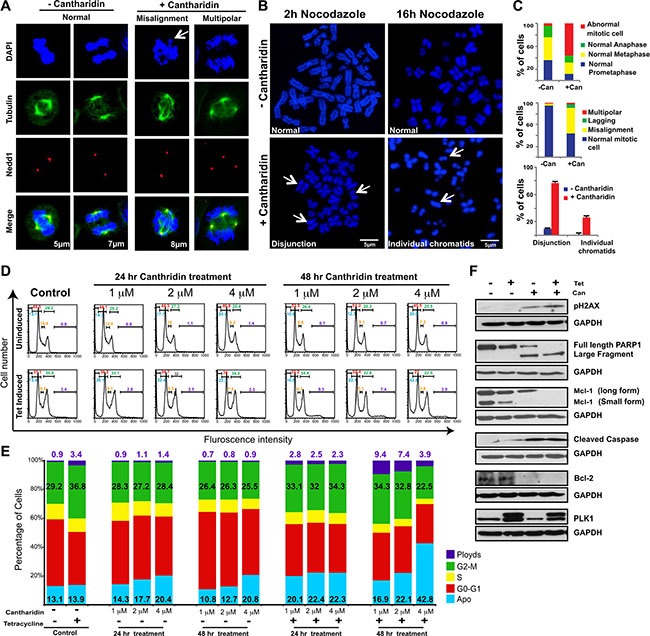
Cantharidin treatment potentiates chromosome segregation anomalies and cell death in PLK1-overexpressing cells (**A**) Representative immunofluorescence pictures showing cantharidin and DMSO-control HCT116-PLK1 cells stained with DAPI and incubated with anti-β-tubulin and anti-Nedd1 antibodies to label microtubules and centrosomes, respectively. DNA is shown in blue, microtubules in green, and centrosomes in red. The first two columns show examples of normal mitotic cells in metaphase and anaphase, respectively. The last two columns show abnormal pre-anaphase mitotic cells, with misaligned chromosomes (left) and a multipolar spindle (right). A bar graph depicting quantification of the number of normal and abnormal mitotic cells in cantharidin and DMSO-treated HCT116-PLK1 populations are shown in (C). (**B**) Representative metaphase spread images and quantification of cantharidin and DMSO-treated HCT116-PLK1 cells after 2 hours and 16 hours with nocodazole. Quantification of the 16 h nocodazole treatment is represented in (C). (**C**) Bar graph quantification of the abnormal mitotic cells from (A) and (B). The quantification of the immunofluorescence for HCT116-PLK1 cantharidin-treated and DMSO control cells is represented in the top and middle graph. *N* = 3 with 30 to 50 cells quantified in each independent experiment and the average of 3 replicates represented. The quantification of the metaphase spread after 16 hours of nocodazole treatment for cantharidin-treated (red) and DMSO control cells (blue) is represented in the bottom graph. *N* = 3 with 30 cells quantified in each independent experiment. Mean ± SD from three independent experiments is represented. (**D**) Representative histograms for the cell cycle analysis of HCT116-PLK1 cells. Control cells and treatment with varying concentrations of cantharidin are shown for uninduced and PLK1-induced HCT116 cells at 24 and 48 hours. The gated values of the histogram represent apoptosis (cyan), aneuploidy (violet), G0-G1 (red), S (yellow), and G2-M (green) for each condition. (**E**) Bar graph quantification of the cell cycle analysis presented in (D). The percentage of cells in each stage for apoptosis is shown in cyan, aneuploidy in violet, G0-G1 in red, S in yellow, and G2-M in green for each condition. *N* = 2. (**F**) Western blot analysis of HCT116-PLK1 uninduced and induced cells treated with cantharidin or DMSO and probed for pH2AX, cleaved caspase 3, and Bcl-2 detection after 24 hours and Mcl-1 and PARP detection after 48 hours of treatment. Levels of small and long form Mcl-1, PARP, pH2AX, Bcl-2, cleaved caspase 3 and PLK1 are represented.

Treatment with cantharidin has previously been shown to activate ERK signaling [47], induce oxidative stress and DNA damage [48], activate the JNK pathway [49], and cause mitotic defects [50]. We hypothesized that the SDL between the mitotic components and PP2A could be due to mitosis related defects and/or DNA damage. Immunofluorescence analysis revealed that cantharidin-treated HCT116 cells exhibited more misaligned chromosome defects, without an increase in multipolar spindles or lagging chromosomes, compared to untreated cells (Figure 3A). While control cells aligned the chromosomes at the metaphase plate when the spindle poles were ~5 to 6 μm apart, over 30% of cantharidin treated cells displayed misalignment of their chromosomes, even when the pole to pole distance had reached over 8 μm (Figure 3A). Accumulation of a large number of cells with improper chromosome alignment prompted us to monitor the pairing of sister chromatids with CENP-A staining (Supplementary Figure S3A). We found PP2A inhibition led to a higher percentage of unpaired, single chromatids at the polar ends, as visualized by one CENP-A dot (Supplementary Figure S3A), indicating that cantharidin treatment might disrupt the cohesion of the sister chromatids.

The Shugoshin family of proteins protects centromeric cohesion and is known to recruit PP2A to the centromere [51]. At the centromere, PP2A maintains the dephosphorylated state of the cohesin ring proteins Rad21/Scc1 and SA2, by counteracting PLK1-mediated phosphorylation, which causes cohesin to dissociate during prophase and be primed for cleavage by Separase at the metaphase-to-anaphase transition [51]. As PP2A prevents precocious separation of sister chromatids, protection of centromeric cohesion could be deregulated by PP2A inhibition. Consistent with this model, metaphase spreads of cantharidin-treated cells displayed premature sister chromatid separation (Figure 3B). We also confirmed that treatment of cells with cantharidin resulted in the cleavage of the cohesin protein Rad21 in PLK1-overexpressing cells [52] (Supplementary Figure S3B). These results suggest that inhibition of PP2A activity in PLK1-overexpressing cells may trigger precocious sister chromatid separation. We used flow cytometry to assess the consequence of cantharidin treatment on PLK1-overexpressing cells. We found that cantharidin treatment of PLK1-overexpressing cells resulted in either increased apoptosis, aneuploidy, or mild G2M delays (Figure 3D, 3E). While previously cantharidin treatment was shown to induce strong G2M arrest, most of these observations used higher doses of cantharidin (> 5 mM)[53, 54]. Here, we found sub-lethal doses of cantharidin induce aneuploidy in spite of constitutive induction of PLK1 using tetracycline. However, treatment with 4 μM cantharidin for 48 hours led to a drop in aneuploidy and to a concurrent increase in apoptosis indicating that these aneuploid cells are not compatible with viability (Figure 3D, 3E).

As PLK1 is also involved in the response to DNA damage [48], we monitored the DNA damage response after cantharidin treatment. A slight increase in the protein level of γ-H2AX within 24 hours after cantharidin treatment in PLK1-overexpressing cells suggested an impaired DNA damage response (Figure 3F). Thus PLK1-overexpressing cells may exacerbate both mitotic defects and DNA damage stress and trigger apoptosis when treated with PP2A inhibitors. Consistent with this idea, we observed an increase in PARP and caspase-3 cleavage in cantharidin-treated PLK1-overexpressing cells as compared to cantharidin treatment or PLK1 overexpression alone (Figure 3F). Similarly, the level of the anti-apoptotic proteins Mcl-1 and Bcl-2 were considerably reduced, indicating that there is a potentiation of apoptosis upon PP2A inhibition in cells with a high level of PLK1 (Figure 3F). An apoptotic PCR array to semi-quantitatively assay the effectiveness of PLK1-overexpression in the induction of apoptotic genes at two different time points after cantharidin treatment showed increased expression of pro-apoptotic genes such as TNF, LTA, GADD45A and CD27 (Supplementary Figure S3C). In contrast, several anti-apoptotic genes like B-cell lymphoma 2 (BCL2), BCL2A1, BCL2L, and AKT were progressively down regulated upon cantharidin treatment in PLK1-overexpressing cells (Supplementary Figure S3D). Overall, our data support the hypothesis that PLK1 overexpression increases the vulnerability of cancer cells to PP2A inhibitors such as cantharidin or nor-cantharidin.

### Unbiased genomic analyses of the alterations in human cancer reveal disparate roles of individual PP2A subunits

**Figure 4:**
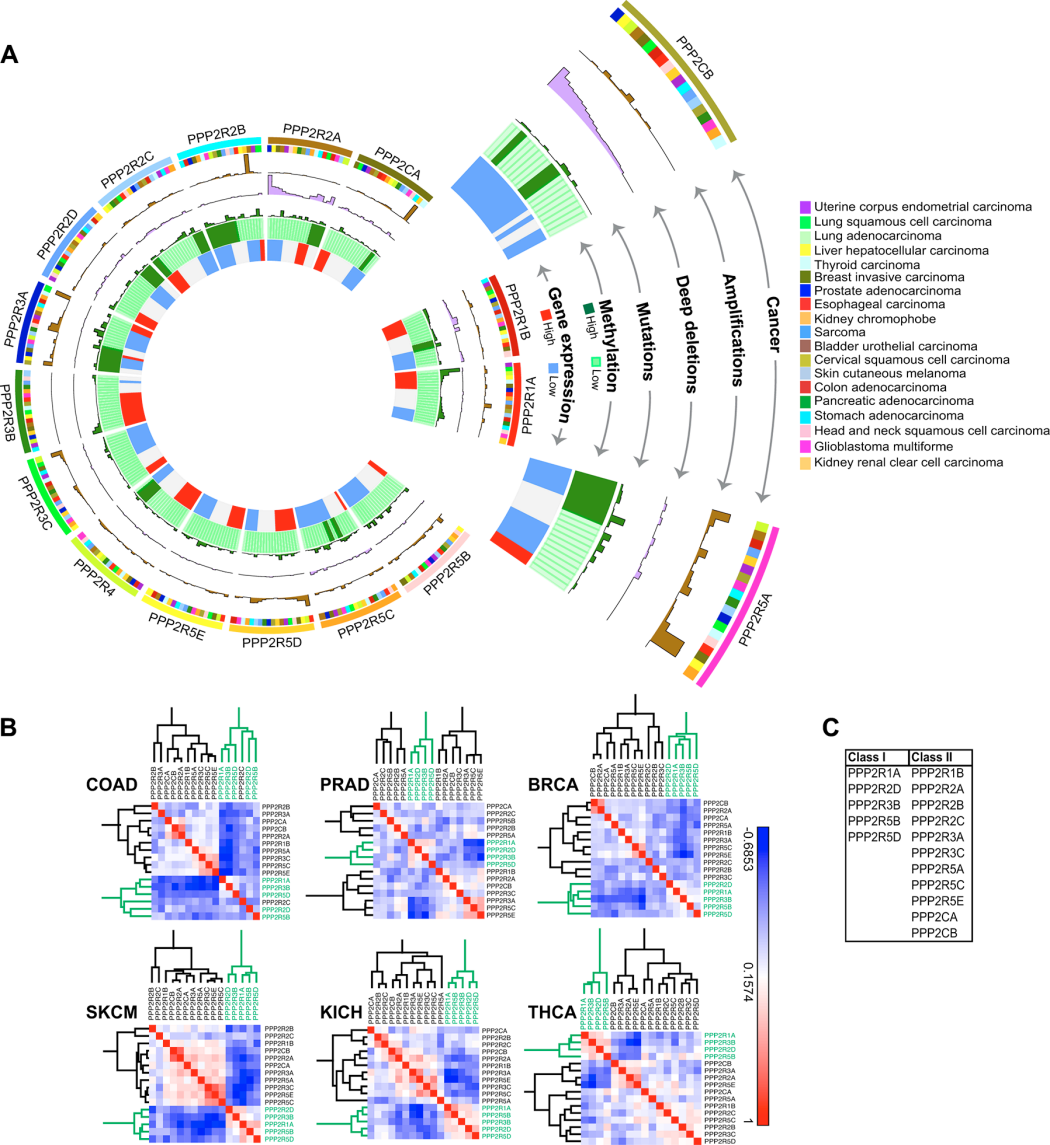
Gene expression analysis and correlation reveals two classes of PP2A subunits (**A**) Circos plot for each of the 17 PP2A subunits analyzed in 19 cancer types. Starting from the inner most ring, each subunit was analyzed for gene expression, methylation, mutations, deep deletions, and amplifications. The outermost ring shows the cancer type for analysis. The abbreviation of each cancer is represented as described in the TCGA portal. (**B**) Representative correlation clustergrams of the PP2A subunits derived from analysis of 19 cancer types. Two distinct classes of PP2A subunits whose expression negatively correlates emerge, highlighted in green. The subunits highlighted in green, PPP2R1A, PPP2R2D, PPP2R3B, PPP2R5B, and PPP2R5D are referred to as Class I. Negative correlations are shown as blue and positive correlations red as represented by the scale. The abbreviation of each cancer is represented as described in the TCGA portal. (**C**) A table listing the two groups of PP2A subunits identified from the clustergram in (B).

Having established that PP2A inhibition potentiates cell death in PLK1-overexpressing cells, we sought to identify the specific complex that is essential for the underlying SDL interaction. The usefulness of cantharidin in the clinical setting is limited by its renal and mucous membrane toxicity [55], and so, identification of the precise complex will allow us to design more specific PP2A inhibitors for therapeutic strategies in the future. To explore whether specific PP2A subunits could be valuable targets for anti-cancer therapy, we took an unbiased approach of systematically analyzing the genomic alterations of each of the subunits across 19 different tumor types. We expected that an in-depth analysis of molecular lesions would provide clues with respect to the role of the individual subunits. Altogether we analyzed data from 8164 patients for expression-based analyses and data from 7099 patients for methylation analyses. Supplementary Table S2 describes the number of patient data analyzed for each cancer type. The complete analyses of the 17-subunit members (including the PP2A activator PPP2R4) are represented in a circos plot (http://circos.ca/), with each layer representing either deep deletion, gene amplification, methylation or gene expression across multiple tumor types (Figure 4A). Only 19 cancer types were analyzed and represented in the circos plot because methylation data were unavailable for the remaining cancer types.

The regulatory subunit PPP2R2A and the catalytic subunit PPP2CB were commonly deleted due to loss-of-heterozygosity or deletions of the 8p chromosome arm [22], whereas the regulatory subunits PPP2R3A, PPP2R5A and PPP2R5D were frequently amplified across multiple tumors types (Figure 4A and Supplementary Figure S4A, S4B). The most commonly mutated PP2A subunit is PPP2R1A. Gain-of-function mutations of PPP2R1A [56] (Haesen and Sablina unpublished) were found in uterine and lung squamous cell cancers (Figure 4A and Supplementary Figure S4C). Promoter methylation analysis (Supplementary Figure S5A) revealed that PPP2R2B, and to some extent PPP2R3A and PPP2R5A are the only subunits predominantly methylated across multiple tumor types (Figure 4A). Consistent with the methylation profiles, gene expression analyses from TCGA dataset revealed that PPP2R2B was extensively down regulated in most tumors compared to normal samples (Supplementary Figure S5B). In contrast, some of the subunits including PPP2R3B, PPP2R1A, and PPP2R5D were highly expressed across multiple tumors (Figure 4A and Supplementary Figure S5B, S5C).

As indicated by our initial analyses, if some PP2A subunits are lost or down regulated, while others are amplified or highly expressed across multiple tumors, we expected a negative correlation in gene expression between these two subsets of PP2A subunits. Consistent with this idea, we observed two distinct classes of the subunits that negatively correlated in expression against each other (Spearman correlation > −0.3; *p* < 0.01). Specifically, we found PPP2R1A, PPP2R2D, PPP2R3B, PPP2R5B and PPP2R5D, clustered together (Class I) and negatively correlated with other PP2A members (Class II) (Figure 4B, 4C). This negative correlation between the two classes of PP2A subunits was observed irrespective of the tissue type (Figure 4B, 4C). Taken together, these genomic analyses strongly suggest that there are two broad classes of PP2A subunits that may have opposing functions during cancer development and progression.

### Class I PP2A subunits exhibit the most SDL interactions with mitotic regulators

**Figure 5:**
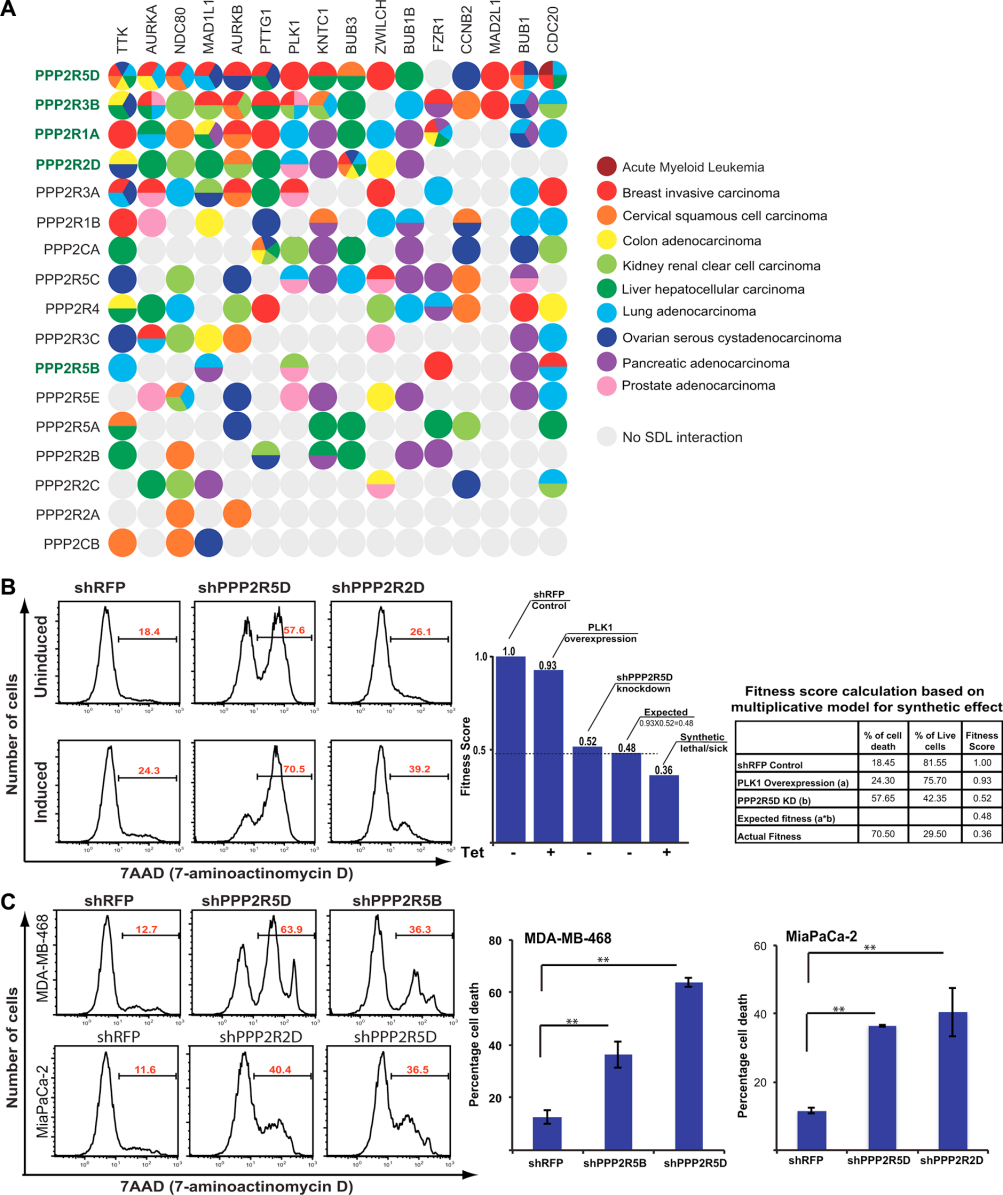
SDL interaction between PP2A subunits and mitotic components (**A**) A dot plot generated by DAISY analysis illustrating the predicted potential SDL interactions between the PP2A subunits and overexpressed mitotic proteins. The predicted interactions are color coded by cancer type. Class I subunits are highlighted in green. (**B**) Representative histograms displaying percentage of cell death (average; *N* = 2) in uninduced and PLK1-overexpressing HCT116 populations for shRFP, shPPP2R5D, and shPPP2R2D. A bar graph showing fitness scores to represent SDL interaction between PPP2R5D and PLK1. The table on the far right shows the conversion of cell death to fitness score. The expected survival fitness of the resultant double mutant based on a multiplicative model is 0.48 (0.93*0.52). Negative deviation (0.36) from the expected fitness is scored as synthetic lethal/sick interaction. (**C**) Representative histograms displaying the increase in cell death for the knockdown of the PP2A subunits PPP2R5D and PPP2R5B in MDA-MB-468 cells (*N* = 2), PPP2R2D and PPP2R5D in MiaPaCa-2 cells (*N* = 2), and non-targeting *sh*RFP control analyzed by 7-AAD staining. The average percentage of cell death is shown on each representative image. Quantification for the selected PP2A subunits knockdowns is shown to the right. **p < 0.05.

While the genomic analysis revealed two classes of PP2A subunits, to selectively target tumor cells that overexpress mitotic genes like PLK1, we decided to take a systematic approach to identify SDL interactions. However, it would be extremely challenging to test all 19 PP2A subunits with about 20 mitotic regulators across multiple cell lines. Hence, we utilized the DAISY method that computationally predicts SDL interactions [57], using mRNA expression (RNAseq) and somatic copy number alteration (SCNA) data of cancer patients from TCGA. We queried the TCGA patient samples and identified several potential interactions with significant *p*-values (FDR < 0.05; Figure 5A; Supplementary Table S3). This prediction algorithm analyzes the expression and SCNA of each PP2A subunit and each specific mitotic regulator in tumors and evaluates if the samples of high activation of mitotic regulator and low activation of the PP2A subunit are depleted as described earlier [57]. Our prediction analysis largely identified most of the Class I regulatory subunits PPP2R5D, PPP2R3B, PPP2R2D (with the exception of PPP2R3A), as well as the structural subunit PPP2R1A, to exhibit strong SDL interactions with several mitotic components (Figure 5A). On the other hand, PPP2CB, PPP2R2A, and PPP2R2C rarely exhibited any SDL interactions (Figure 5A). Interestingly, some of the subunits exhibited SDL interaction with a given mitotic component across multiple cancer types. For example the SDL interaction between PPP2R5D and TTK or PPP2R2D and BUB3 were identified across six different cancers (Figure 5A). Also some of the mitotic components like TTK exhibited SDL interactions with numerous PP2A subunits. These analyses strongly indicated that Class I subunits may represent the targetable vulnerabilities of the PP2A complex.

To experimentally validate, we first tested the SDL interactions between PLK1 and the PP2A subunits in the PLK1-inducible HCT116 cells. Only those PP2A subunits that are expressed in this cell line were considered. Consistent with the computational prediction, we found the Class I members, PPP2R5D, PPP2R1A, and PPP2R2D to cause selective lethality to PLK1-overexpressing cells (Figure 5B). We also tested the SDL interaction of PP2A subunits in breast and pancreatic cell lines that naturally overexpress PLK1. Given the heterogeneity among these cell lines, we did not expect the loss of the same PP2A subunits to cause lethality. In fact, according to the computationally predicted SDL interactions the same SDL interaction between any pairs of genes is not found across all cancer types (Figure 5A). Yet, we found PPP2R5D, and to some extent PPP2R2D and PPP2R1A, were among the top genes that exhibited SDL in MDA-MB-468 and MiaPaCa-2 cells (Figure 5C and Supplementary Figure S6A). Thus, we expect most Class I genes to be strongly exhibiting SDL interactions with different mitotic regulators. The efficiency of the knockdown was monitored by qPCR, except for PPP2CA, as the transcript level could not be quantified due to cell death upon knockdown (Supplementary Figure S6B).

**Figure 6:**
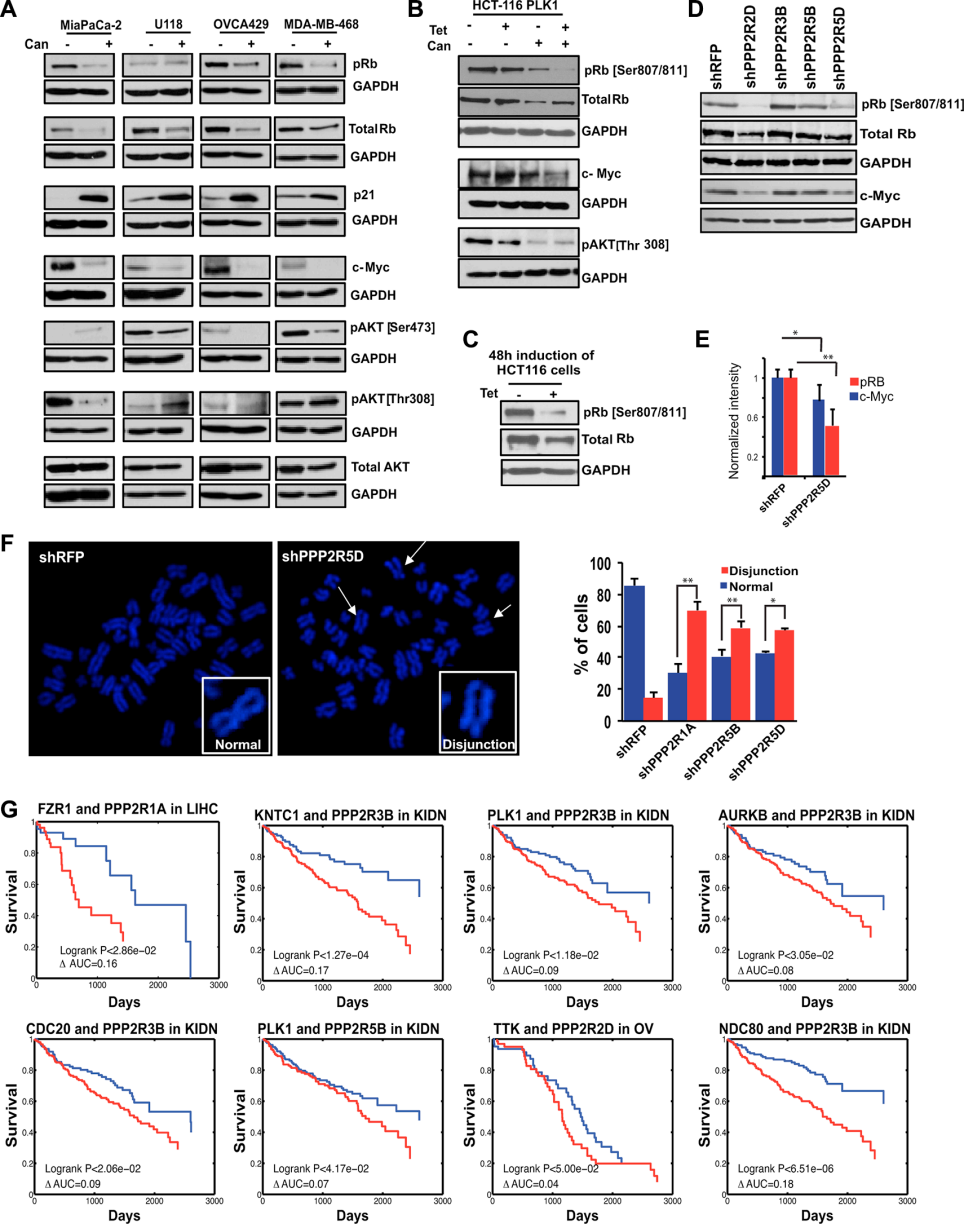
Identification of the PP2A regulatory subunit required for the survival of cancer cells and its clinical significance (**A**) Western blot analysis of naturally PLK1-overexpressing cancer cell lines in response to PP2A inhibition by cantharidin treatment. Levels of pRb (Ser 807/811), p21, c-Myc, pAKT (Ser473 and Thr308), and total AKT are represented with GAPDH as a loading control for MiaPaCa-2 pancreatic cells, U118 glioblastoma cells, OVCA429 ovarian cells, and MDA-MB-468 breast cancer cells. (**B**) Western blot analysis of HCT116-PLK1 uninduced and PLK1-induced cells after 24 hours with and without cantharidin treatment. Levels of pRb, total Rb, c-Myc, and pAKT [Thr308] are shown, with GAPDH used as a loading control. (**C**) Western blot analysis of pRb and total Rb in HCT116-PLK1 cells after 48 hours of induction. GAPDH is used as a loading control. (**D**) Western blots showing pRb, total Rb, and c-Myc in knockdowns of Class I PP2A subunits in MiaPaCa-2 cells. (**E**) Bar graph quantification showing the decrease in pRb (red) and c-Myc (blue) levels from (D). *p < 0.05; **p < 0.005. (**F**) Representative metaphase spread pictures for *sh*PPP2R5D and *sh*RFP and bar graph quantification for the PPP2R1A, PPP2R5D, and PPP2R5B knockdowns in HCT116-PLK1 cells relative to the non-targeting *sh*RFP control, with the percentage of cells displaying chromosome disjunction shown in red and the percentage of cells with normal chromosome alignment shown in blue. (**G**) Kaplan-Meier survival curves of patients which have a naturally occurring SAC-PP2A SDL interaction, in blue, and those who do not, in red. *p < 0.05; **p < 0.005.

To gain the mechanistic insight, we monitored how treatment with cantharidin affected the levels of a range of signaling proteins that regulate cell cycle in multiple cell lines that naturally overexpress PLK1. We found that cantharidin treatment altered the levels of pAKT, p21, c-Myc, and pRb, including the total Rb, in most of these cell lines that naturally overexpress PLK1 (Figure 6A). However, we found pRb and c-Myc were the only proteins that consistently decreased in the PLK1-induced, cantharidin-treated HCT116 cells compared to cantharidin treatment or PLK1 overexpression alone (Figure 6B). This suggested that the down regulation of these two proteins might play a key role in selectively affecting the proliferation of cantharidin-treated and PLK1-overexpressing cells. For example, levels of pAKT decreased irrespective of PLK1 overexpression, upon cantharidin treatment (Figure 6B). Interestingly, we also found that the levels of pRb and to some lesser extent total Rb, slightly decreased when PLK1 overexpression was induced over a prolonged duration (Figure 6C). This prompted us to assess the levels of pRb, total Rb and c-Myc when the Class I PP2A subunits were depleted. We found that loss of PPP2R5D and PPP2R2D resulted in consistent decrease in pRb and c-Myc but not total Rb in the PLK1-overexpressing MiaPaCa-2 cells (Figure 6D, 6E). While cantharidin treatment caused decrease in both total and pRb, loss of PPP2R5D and PPP2R2D did not affect the levels of total Rb. We reasoned that the total Rb level could be affected possibly due to the overall inhibition of multiple PP2A subunits by cantharidin and perhaps, to some extent by PP1 inhibition.

As previous studies have shown that loss of pRb leads to defects in chromosome condensation and cohesion [58], abnormal centromere structure [59], and accumulation of DNA damage [60], we hypothesized that overexpression of PLK1 in cantharidin-treated cells may cause excessive genotoxic insults that may be incompatible with viability. Consistent with this hypothesis, as cantharidin treatment led to the formation of chromosome disjunction and cohesion defects (Figure 3B), we next asked which of the Class I subunits phenocopy these cohesion defects upon depletion. We found that loss of PPP2R1A, PPP2R5B and PPP2R5D led to chromosome disjunction defects (Figure 6F). The remainder of the Class I subunits did not reveal any significant cohesion defects. Taken together, our analyses consistently identified loss of PPP2R5D as one of the important genetic vulnerability that affected both the pRb-pathway as well as sister chromatid cohesion in PLK1-overexpressing cells.

### Loss of Class I subunits provide significant clinical benefits to patients that naturally overexpress PLK1

While we have made efforts to show that targeting PP2A may selectively kill tumor cells overexpressing mitotic regulators, to evaluate the translational potential of these targets, we next asked if the patients with overexpression of the mitotic components, including PLK1, would actually benefit from targeting specific PP2A complexes. To find this, we focused on the targetable Class I subunits and categorized the TCGA patient data into two groups, one with mitotic gene overexpression (Group A) and the other without mitotic gene overexpression (Group B). Analysis of this data determined if the survival benefit of down regulation of the Class I subunits of PP2A is specifically significant in group A and not in group B. We performed this analysis for multiple cancer types for which significant clinical information is available and the complete analysis is provided in Supplementary Table S4. While loss of several Class I subunits and high expression of mitotic components conferred longer survival with a significant difference in clinical prognosis as determined by Kaplan-Meier analysis (log-rank *p*-value < 10^−7^) (Figure 6G), we did not observe any significant benefit to the patients who have lost the expression of the Class II catalytic subunit PPP2CB. In summary, our work proposes a provocative link between mitotic proteins and PP2A subunits, demonstrating the targetable vulnerabilities associated with PP2A inhibition.

## DISCUSSION

The detection of SDL interactions in cancer cells and how they may be applied in cancer therapeutics is gaining tremendous interest [61]. Current therapeutic strategies heavily depend on targeted sequencing of tumor biopsies that specifically account for the presence or absence of a given driver mutation. However, it is now clear that multiple drivers within a single tumor provide selective growth advantage to the tumor cells [3]. Recent studies that used multi-region sequencing have shown that the majority of driver mutations are heterogeneous and that an individual mutation might not be detected homogeneously across all regions of the same tumor [62, 63]. Thus for effective treatment, it is ideal to have a complete blueprint of the tumor landscape that accounts for any clonal or sub-clonal frequencies of a driver alteration even before beginning the treatment. However, generating such information is challenging. Given the degree of tumor heterogeneity, it is imperative to identify possible targets that, when inhibited using an appropriate selection rationale, can potentially overcome tumor heterogeneity and impart an improved clinical outcome. Inabilities to deal with tumor heterogeneity will typically lead to disease recurrence and missed opportunities to eradicate the entire tumor. It is, therefore, crucial to determine the context in which a given gene may function as a target to overcome tumor heterogeneity. Our present work provides the framework for such a context, i.e. PLK1 overexpression, in which PP2A inhibition can be effective in killing the tumor cells. As PLK1 overexpression induces gain or loss of chromosomes (Figure 1C), we expect that PLK1 overexpression can be used as a model to capture the heterogeneous tumor population. Our work demonstrates that inhibiting selective members of the PP2A complex has the potential to mitigate tumor heterogeneity associated with PLK1 overexpression.

Our work has also for the first time taken into account the expression and essentiality of all the PP2A subunits from patient data across multiple tumor types to establish a more detailed view of the distinct functional roles of different PP2A subunits. Importantly, our investigation provides some critical insight as to which specific subunits may function as potential targets. This is in contrast to much of the earlier work describing PP2A as a tumor suppressor, and points out the complexity of roles for PP2A that depend upon the specific subunits of PP2A expressed in different cell types and how they may come together in different trimeric PP2A complexes with distinct cellular roles.

Recent genomic sequencing analyses, copy number variation (CNV) studies, and functional analysis of the mitotic protein levels in real tumors suggest that mitotic proteins are often overexpressed in CIN cancers[10] (Figure 1A and Supplementary Figure S1). Up regulation of the mitotic checkpoint components *MAD1* or *MAD2* or *BUB1* is sufficient to induce CIN, suggesting that overexpression of checkpoint components play a key role in tumor formation [9, 18–20, 64]. Consistent with this idea, inhibition of MPS1, a key regulator of the mitotic checkpoint, by the small-molecule inhibitor NMS-P715 was sufficient to cause cell death in a variety of tumor cell lines and inhibit tumor growth in preclinical cancer models [65]. Despite these findings, there were still concerns that direct disruption of the mitotic checkpoint might facilitate CIN [11–13]. Complete mitotic checkpoint inhibition could be detrimental to normal cells, and this could defeat the tumor-selective basis of the approach [13]. However, selective inhibition of Class I PP2A subunits may provide a unique opportunity to potentiate apoptosis, when the mitotic components are overexpressed. Although we highlight the potential of targeting Class I subunits, we also note that the current efficiency of this selective killing is still marginal. For example, ~14 to 18% more killing alone was observed for the PLK1-overexpressing HCT116 cells when PPP2R5D is knocked down (Figure 5B). We believe that the identification of the trimeric PP2A complex and the corresponding substrate essential for sensitizing PLK1-overexpressing cells will further improve the development of more effective therapies. For example, use of phospho-proteomic approaches to identify the specific substrates of the Class I subunits and disrupting these substrate-specific interactions may be highly beneficial for sensitizing PLK1-overexpressing cells.

While we mostly investigated the SDL relation between PP2A and PLK1, we note from our chemical genetic analysis (Figure 2B) that the SDL relation of PP2A appears to be broadly applicable across many cell types overexpressing different mitotic regulatory proteins to varying degrees. Compared to previous studies [66] that evaluated the usage of cantharidin, here we show that the overexpression of mitotic components decreased the required dosage of this inhibitor ~3 to 5 fold, all the while achieving the same effect as seen with a standard dose of 8 to 10 micromolar [47, 67, 68]. Moreover, both cantharidin and its de-methylated analog nor-cantharidin are lipid soluble and less than 400 Daltons in size. These features are important requirements to cross the blood brain barrier. As such, our investigation on the selective killing of the glioblastoma cells that overexpress PLK1 by these molecules (Figure 2B) warrants further studies focused on brain tumors. In fact, a nor-cantharidin derivative, designed as a microsphere by linking with a long-chain saturated alkane group has been generated to improve its solubility (Patent # CN102973503 A). It is also noteworthy to point out that ongoing clinical trials with a more specific proprietary PP2A inhibitor, LB-100, may be less toxic and thus of more benefit to cancer patients [69].

One of the potential mechanism that can mediate the SDL interactions between the mitotic regulators like PLK1 and PPP2R5D could be defective cohesion between sister chromatids caused by a decrease in pRb, which in turn can induce precocious separation of sister chromatids and subsequent cell death [58–60]. Consistent with this, previous proteomic analysis identified the interaction between shugoshin and the isoforms of the B56 regulatory subunits of PP2A [70]. While this mechanism requires loss of PPP2R5D to cause decrease in pRb as we observed (Figure 6D), previous studies have also shown that PP2A can also control the phosphrylation status of CDKs, perhaps Cdk2-CyclinE and/or Cdk4-CyclinD that are known to maintain the phosphorylation of Rb [71, 72]. Thus, PPP2R5D loss may indirectly mediate a decrease in Rb phosphorylation. Another potential mechanism of the SDL is the inability of the spindle checkpoint protein BubR1 to recruit the B56 regulatory subunits upon PLK1-dependent phosphorylation [73, 74].

With emerging clinical successes in the usage of protein kinase inhibitors, it is clear that altering the signaling mechanisms within the cells can provide therapeutic benefits. Our work focused on the inhibition of the PP2A complex in the context of mitosis because of the SDL relation with mitotic components. However, the PP2A complex constitutes about one percent of cellular protein [75] with roles in multiple intricate cellular processes that control both cell growth and apoptosis. We anticipate that a systematic genome-wide SDL interaction network of the Class I subunits of PP2A combined with a synthetic lethal analysis with the tumor suppressor subunits of PP2A will reveal the complete network of cross-talk for this phosphatase family.

## MATERIALS AND METHODS

### Ethics statement

Investigation has been conducted in accordance with the ethical standards and according to the Declaration of Helsinki and according to national and international guidelines and has been approved by the authors' institutional review board.

### Cell culture, transfections, and transductions

Unless otherwise noted, all cell lines were cultured according the ATCC guidelines. HCT116-PLK1 and DLD1-MAD1 cells [9, 32] were induced with 500 ng/mL tetracycline (TET). Ethanol diluted in media (35%) served as the vehicle control for TET. Cells were treated with 4 μM cantharidin (Cayman Chemical) or DMSO for the vehicle control in all experiments unless stated. Transfections were carried out using X-tremeGENE 9 (Roche) as per the manufacturer's instructions. Pooled lentivirus containing five *sh*RNA sequences specific to each PP2A subunit and shRFP for a non-targeting control (Sigma) were generated by transfecting HEK293T cells with psPAX2, pMD2.G, and pLKO.1 containing the PP2A-*sh*RNA sequences. Media was replaced 18 hours after transfection with DMEM containing 2% (w/v) bovine serum albumin (BSA) and lentivirus was collected after 24 and 48 hours. Transducing cells with pooled PP2A- specific *sh*RNA lentiviruses generated stable PP2A subunit knockdowns. Transduced cells were selected with 2 μg/mL puromycin for 48 hours before assays were performed.

### Cell viability assays

Cells were seeded in 96 well plates and treated with varying concentrations of cantharidin and norcantharidin (Sigma). Stock solutions of cantharidin and norcantharidin were made in DMSO. After 48 hours of treatment, cell viability was analyzed using resazurin as per the manufacturer's recommendations (Fisher Scientific).

### FACS analysis

PP2A knockdown HCT116-PLK1 cells were induced with 500 ng/mL TET or ethanol vehicle control for 24 hours before FACS analysis. Single induction or constitutive inductions were done as shown in the schematic of Supplementary Figure S2A. Cell viability, cell cycle and aneuploidy were determined by propidium iodide (PI) (Thermo Fisher) staining following the manufacturers' recommendations. Apoptosis assays were performed using 7-AAD (BD Biosciences) as per the manufacturer's recommended procedure. Etoposide (Abcam) treated cells were used as a positive control for cell death. All data were analyzed using FlowJo Software (version 9.9 for Mac).

### Western blot and antibody array analysis

Protein lysates were collected and quantified using a bicinchoninic acid (BCA) assay (Thermo Scientific, 23225). Cell lysates containing 40 μg of protein were electrophoresed and probed using the antibody manufacturers' recommended procedure. The following antibodies were obtained from Santa Cruz: Mcl-1 (sc-819), RAD21 (sc-166973), GAPDH (sc-25778), c-Myc (sc-40), total Rb (sc-102), and PLK1 (sc-5585). The following antibodies were obtained from Cell Signaling: total AKT (9272), phospho-AKT [Ser473] (9271), phospho-AKT [Thr308] (13038), phospho-Rb (9308), p21 (2947), Bcl-2 (2870), PARP (9542), and cleaved caspase-3 (9661). The pH2AX antibody (05636) was obtained from EMD Millipore and the MAD1 (ab175245) and PLK1 (ab17057) antibodies from Abcam. Membranes were blocked with 5% milk in TBST or BSA for phospho-antibodies, and primary antibodies were diluted 1:1000 in blocking solution. Primary antibodies were detected with peroxidase-conjugated secondary antibody diluted 1:10,000 in blocking solution (Thermo Scientific, 31431, 31466) with ECL Western Blotting chemiluminescent substrate (Thermo Scientific) following the manufacturer's procedure.

### Indirect immunofluorescence microscopy

HCT116-PLK1 cells were treated with TET induction and/or cantharidin and fixed with 100% cold methanol for 10 minutes and permeabilized in 0.25% Triton X-100. Antibody incubation was carried out in 1% BSA in PBS for 1 hour at 37°C with the following antibodies from Abcam: NEDD1 (abcam57336), β-tubulin (abcam6046), CENPA (abcam13939). The γH2AX antibody (2607372) was obtained from EMD Millipore. Primary antibodies were detected with Alexafluor-594 and Alexafluor-488-conjugated secondary antibodies (Life Technologies, A11005, A11034). DNA was stained using DAPI. Three dimensional image stacks of mitotic cells were acquired in 0.2μm steps using a 63X oil-immersion objective on an FV300 confocal laser scanning biological microscope (Olympus). Image stacks were deconvolved using Fiji software. Centromere distance measurements were made manually using FV10-ASW 4.0 Viewer.

### Metaphase spread analysis

Cell cultures were incubated with 100 ng/mL nocodazole, trypsinized, and resuspended in 75 mM KCl for 15 minutes at room temperature, and then fixed with an ice-cold methanol and acetic acid (3:1, v/v) solution. Fixed cells were dropped on glass microscope slides, stained with DAPI, and imaged using a 63× oil-immersion objective lens on an FV300 confocal laser scanning biological microscope.

### Quantitative real-time PCR analysis

RNA was isolated from cell pellets using an RNeasy mini kit (Qiagen) according to the manufacturer's instructions including DNase treatment (Qiagen). RNA quantification and integrity was verified spectrophotometrically. An equal amount of RNA was used for cDNA conversion using the RT^2^ First strand kit (Qiagen) according to the manufacturer's instructions. Apoptosis-related gene expression was evaluated using RT^2^ Profiler human apoptosis PCR arrays (Qiagen, 330231) according to the manufacturer's instructions. Data analysis was performed using the ΔΔCT method as described in the manufacturer's web portal (SABiosciences). PP2A *sh*RNA knockdown was confirmed using Taqman real-time PCR gene expression assays (Life Technologies, 4331182 - assay ID's: Hs00953658_m1, Hs00270227_m1, Hs00739033_m1, Hs00396777_m1, Hs00160407_m1, Hs00203045_m1, Hs00215595_m1, Hs00196542_m1, Hs00196561_m1, Hs00604899_g1, Hs00605059_m1, Hs00952135_m1, Hs00427260_m1, Hs00602137_m1, Hs01026388_m1, Hs00988483_m1, Hs00603515_m1) following the manufacturer's instructions.

### Computational analysis

Gene expression analysis to compare the expression of a query gene with a normal counterpart was done across 24 different cancers. The circos plot was generated using data for only 19 cancers that had methylation data available. To calculate the SDL interactions between mitotic genes and PP2A genes, a much larger dataset containing gene expression (RNA-Seq), SCNA, and patient survival data was required for each cancer type and so TCGA patient data from 10 different cancer types of large sample sizes were analyzed. The evaluation was performed by molecular screening that determines a gene pair as an SDL pair if mitotic components up regulated and PP2A-down regulated samples are significantly depleted because such genetic state will be selected against in tumor population as described in Jerby-Arnon et al. [57]. This is followed by multiple hypothesis correction to the maximal *p*-value based on mRNA and SCNA. Kaplan-Meier survival analysis determines the clinical relevance of the SDL interaction by comparing the survival of mitotic gene up regulated and PP2A down regulated samples to the survival of the samples where mitotic regulators are up regulated and PP2A is not down regulated. We controlled for the false positive discovery by filtering the pairs where PP2A down regulation significantly improves patient survival irrespective of mitotic component up regulation.

To analyze the expression of PLK1 in the previously annotated, cantharidin-sensitive and cantharidin-resistant cell lines [36], we used the gene expression profile from the CCLE database. Briefly, we used the cytotoxicity of the NCI panel of cell lines to identify those cell types that had low IC50 and high IC50. From this, we analyzed the expression of PLK1 for only those top cell lines for which gene expression data is available from the CCLE database. We also used the CCLE database to determine median expression of PLK1 across multiple cell lines and classify them as two groups of cell lines that over/under express PLK1. Based on this expression-based classification and the availability of cell lines, we selected cell lines that naturally overexpress or underexpress PLK1.

## SUPPLEMENTARY MATERIALS FIGURES AND TABLES





## Abbreviations

CIN: chromosomal instability
SDL: synthetic dosage lethality
PP2A: protein phosphatase 2A
PLK1: polo-like kinase 1
TET: tetracycline
TNBC: triple negative breast cancer
CCLE: Cancer Cell Line Encyclopedia
PI: propidium iodide
BCL-2: B-cell lymphoma 2
SCNA: somatic copy number alteration
TCGA: The Cancer Genome Atlas
pAKT: phospho-AKT
CNV: copy number variation
pRb: phospho-Rb
GBM: glioblastoma.
