# Supplementary material for: Therapeutic relevance of the protein phosphatase 2A in cancer
Source: Oncotarget. 2016 Aug 19;7(38):61544–61. doi: 10.18632/oncotarget.11399 (PMC5308671; doi:10.18632/oncotarget.11399)
Supplement: Supplementary file 1 [file oncotarget-07-61544-s001.pdf]

Therapeutic relevance of the protein phosphatase 2A in cancer

Supplementary Materials

Supplementary Table S1: Table describing the alternative names of the PP2A subunits for easy reference

Gene	GeneID	Alternative	Subunit Type
PPP2CA	5515		catalytic subunit
PPP2CB	5516		catalytic subunit
PPP2R1A	5518	PR56α	Structural subunit
PPP2R1B	5519	PR56β	Structural subunit
PPP2R2A	5520	B55α	regulatory subunit B
PPP2R2B	5521	B55β	regulatory subunit B
PPP2R2C	5522	B55γ	regulatory subunit B
PPP2R2D	55844	B55δ	regulatory subunit B
PPP2R3A	5523	PR72	regulatory subunit B''
PPP2R3B	28227	PR48	regulatory subunit B''
PPP2R3C	55012		regulatory subunit B''
PPP2R5A	5525	B56α	regulatory subunit B'
PPP2R5B	5526	B56β	regulatory subunit B'
PPP2R5C	5527	B56γ	regulatory subunit B'
PPP2R5D	5528	B56δ	regulatory subunit B'
PPP2R5E	5529	B56ε	regulatory subunit B'
PPP2R4	5524	PTPA	activator, regulatory subunit 4

Supplementary Table S2: Summary of the patient samples downloaded and analyzed from the TCGA to generate Figure 4A

Abv.	Expression		Methylation		cBioPortal			
	Tumor	Normal	Tumor	Normal	Patients	Amplification	Deletion	Mutation
BLCA	405	19	419	21	127	28	19	21
BRCA	1084	100	799	98	963	255	148	45
CESC	297	3	309	3	191	27	22	23
CHOL	36	9						
COAD	476	41	315	38	220	6	25	36
ESCA	173	11	186	16	186	64	20	0
GBM	145	5	153	2	273	16	4	3
HNSC	515	44	529	50	279	79	28	26
KICH	66	25	66	1	66	3	1	2
KIRC	527	72	323	159	415	145	19	15
KIRP	289	32						
LIHC	369	51	380	50	193	40	28	24
LUAD	512	58	475	32	230	80	38	35
LUSC	498	51	372	43	178	50	31	32
PAAD	178	4	184	10	145	16	4	18
PCPG	176	3						
PRAD	483	51	503	50	332	32	110	14
READ	163	10						
SARC	258	2	265	4	240	49	31	12
SKCM	104	1	472	2	278	39	37	65
STAD	413	35	395	2	287	45	30	106
THCA	500	57	515	56	399	10	3	4
THYM	120	2						
UCEC	540	35	439	46	242	41	17	124

Gene	Number of Methylation Sites
PPP2CA	20
PPP2CB	9
PPP2R1A	13
PPP2R1B	20
PPP2R2A	17
PPP2R2B	47
PPP2R2C	47
PPP2R2D	39
PPP2R3A	14
PPP2R3C	12
PPP2R4	11
PPP2R5A	17
PPP2R5B	15
PPP2R5C	50
PPP2R5D	17
PPP2R5E	17
STRN	9
STRN3	25

Supplementary Table S3: FDR-corrected *p* values for the SDL interactions between the mitotic regulators and the PP2A subunits across different cancer types. Significant interactions are represented in Figure 5A. See Supplementary_Table_S3

Supplementary Table S4: Clinical significance data for all possible combinations for the Class I subunits with multiple mitotic regulators across different cancer types with sufficient clinical data. See Supplementary_Table_S4

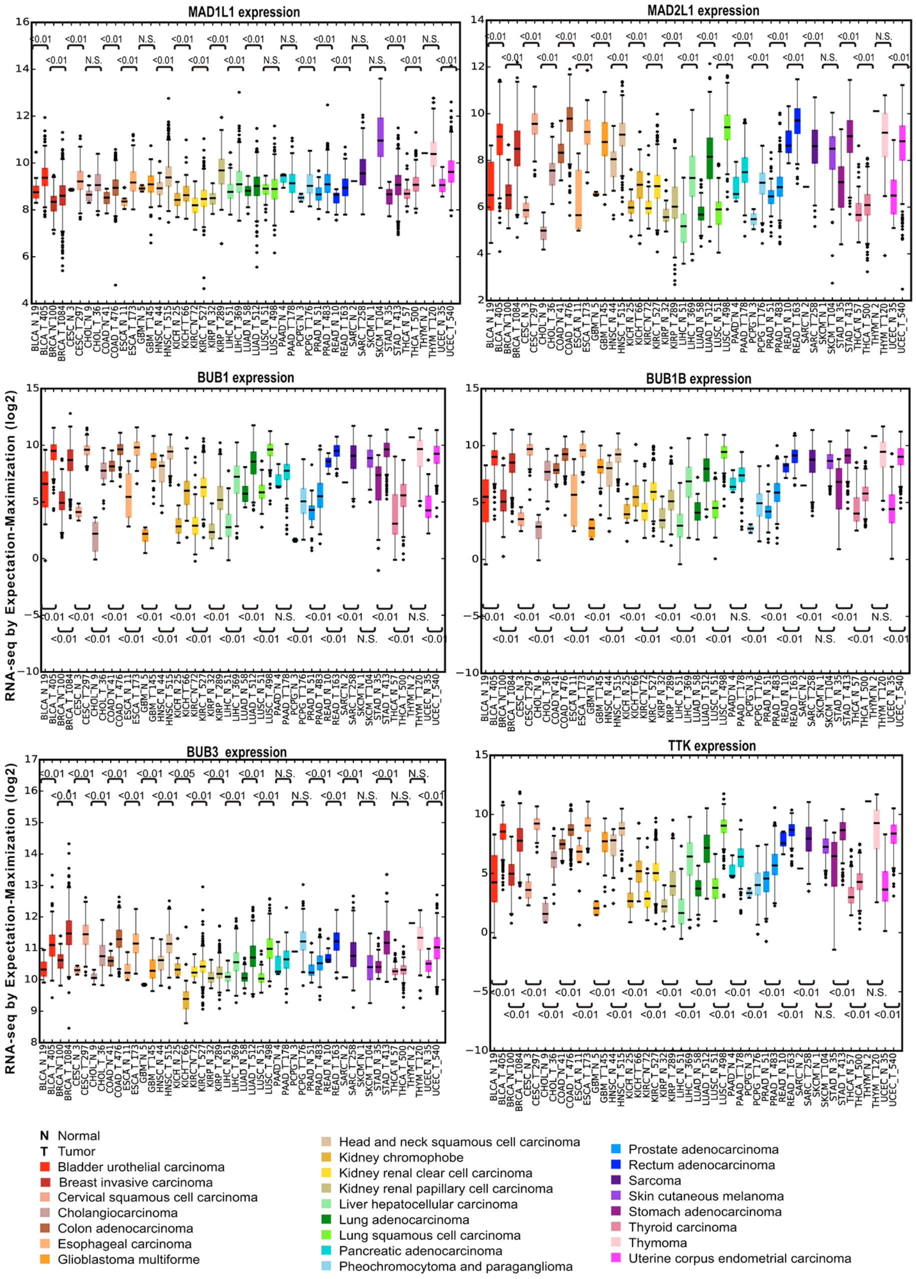

Supplementary Figure S1: Many mitotic components are overexpressed in a variety of tumor types. Expression scores for mitotic regulator proteins (MAD1L1, MAD2L1, BUB1, BUB1B, BUB3, and TTK) within 24 different types of cancer and normal tissue from TCGA. The numbers in x-axis labels denotes the number of patient samples in each cancer type. Statistical significance of the difference in expression between the normal and tumor samples are depicted for each cancer type. N.S. denotes not significant. The abbreviation of each cancer in the axis label is represented as described in the TCGA portal.

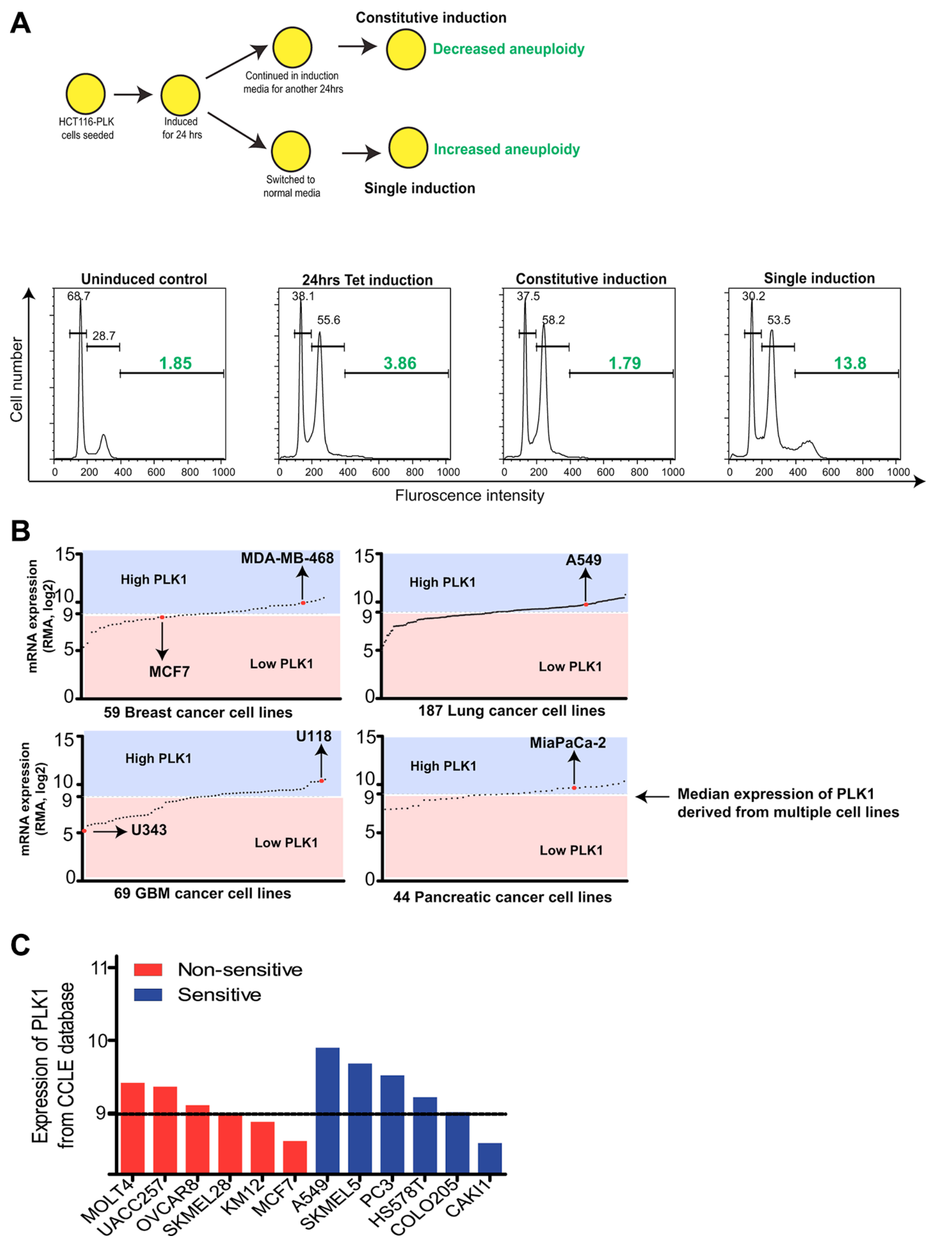

Supplementary Figure S2: PLK1 overexpression, aneuploidy, and cantharidin sensitivity. (**A**) A schematic of the constitutive versus single induction of HCT116-PLK1 cells with representative histograms showing the percentage of aneuploid cells in green. (**B**) Graphs showing the expression of PLK1 in different cancer types highlighting the cell lines used. The high and low classification was derived from the median expression of PLK1. (**C**) Expression levels of PLK1 in cell lines classified as either cantharidin-sensitive (blue) or insensitive (red).

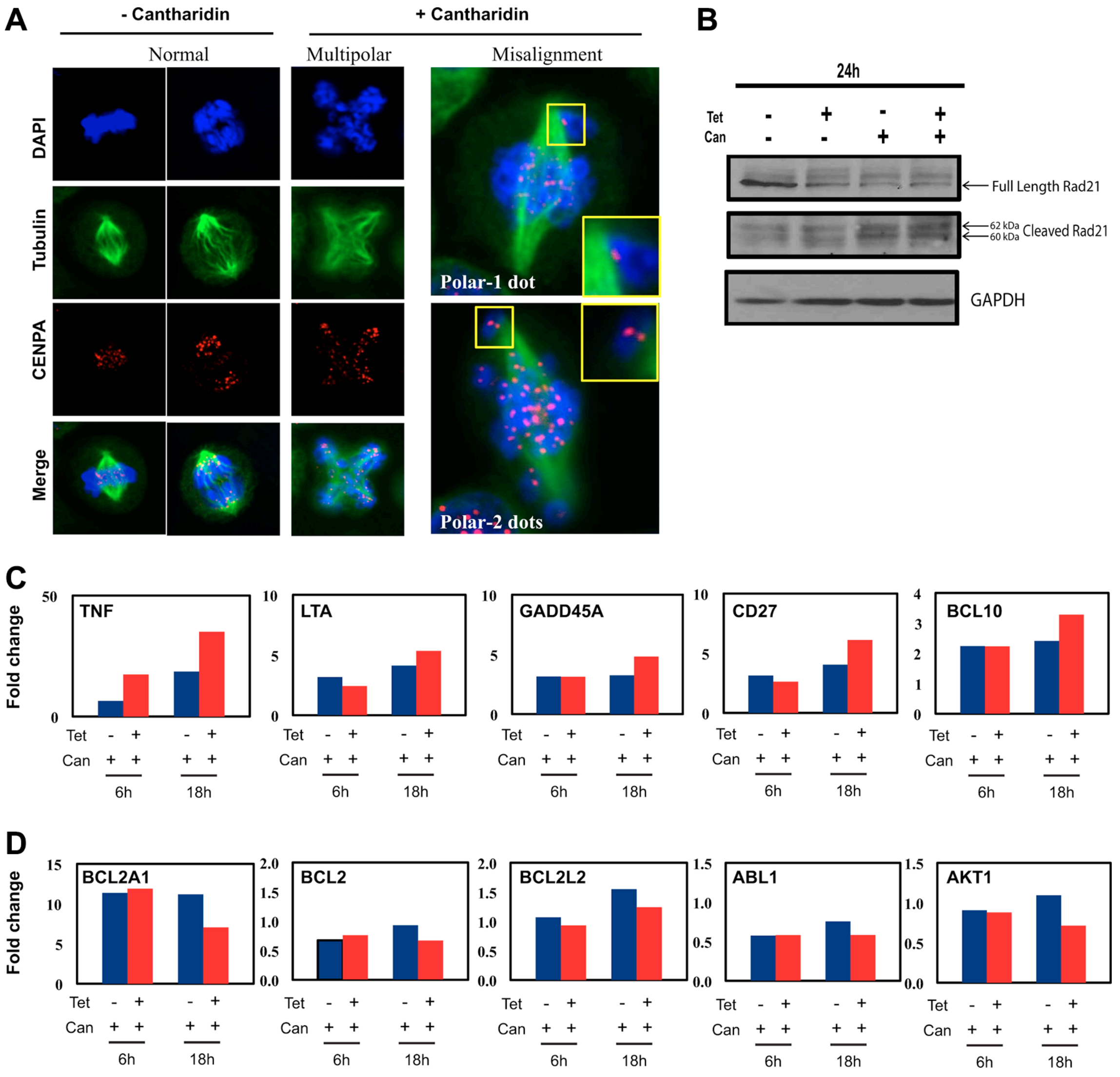

Supplementary Figure S3: Cantharidin treatment causes increased DNA damage and cell death. (**A**) Representative immunofluorescence pictures showing cantharidin and DMSO-treated HCT116 cells stained with DAPI and incubated with anti-β-tubulin and anti-CENPA antibodies. DNA is shown in blue, microtubules in green, and centromeres in red. The first two columns show examples of normal mitotic cells, while the last three columns are abnormal mitotic cells exhibiting chromosome misalignment or multipolar spindles. (**B**) Western blot analysis showing full length and cleaved Rad21 in HCT116-PLK1 cells uninduced and induced with TET for PLK1 expression and treated with cantharidin or DMSO for 24 hours. (**C** and **D**) Bar graphs showing the fold change in apoptosis-related gene expression in uninduced and induced HCT116-PLK1 cells treated with cantharidin or DMSO as analyzed by apoptotic PCR array. Genes promoting the death of cancer cells that showed an increase with cantharidin treatment are pictured in (C) and genes promoting the survival of cancer cells that showed a decrease with cantharidin treatment are pictured in (D).

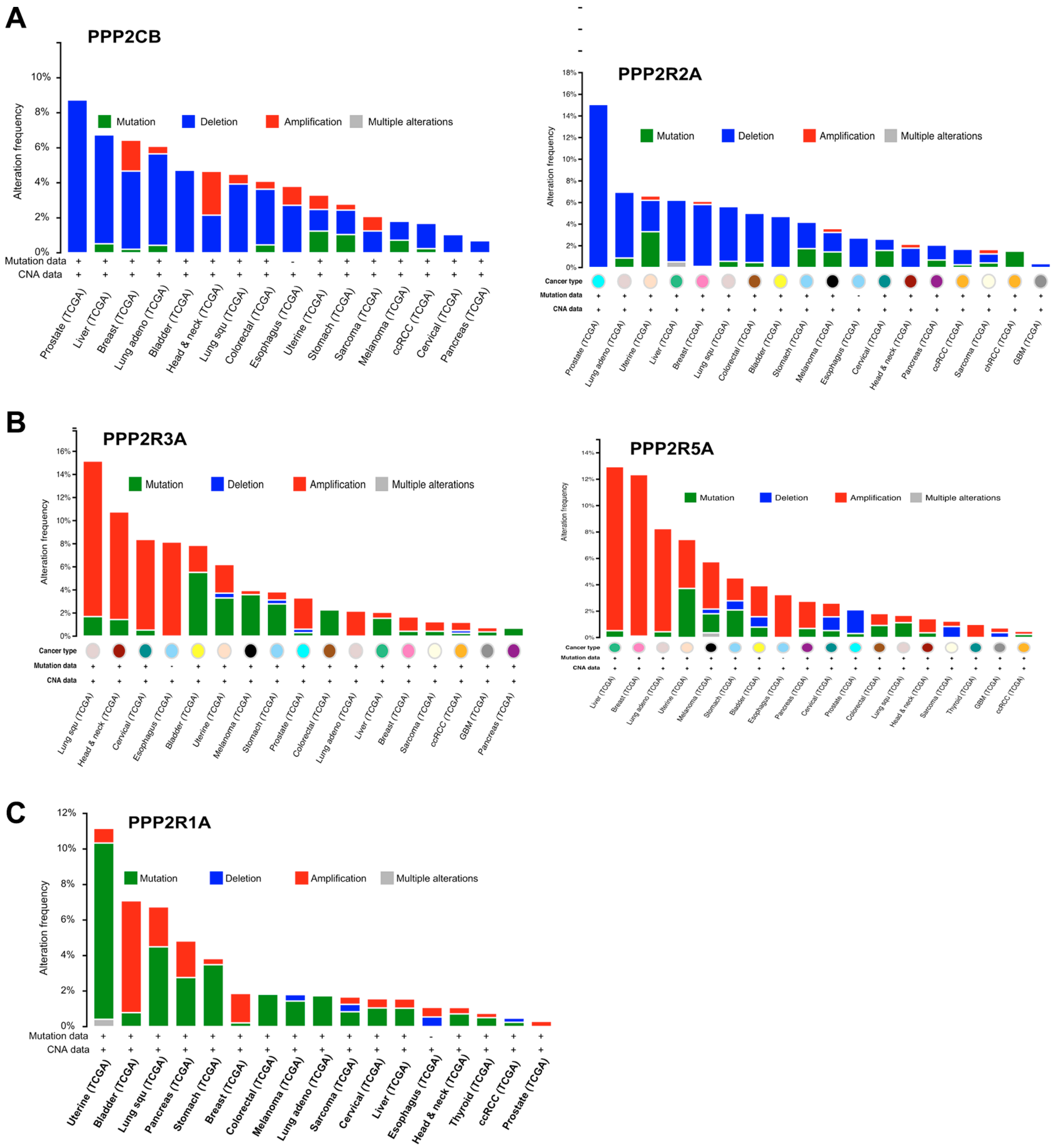

Supplementary Figure S4: PP2A subunits are frequently altered in cancer. (**A** to **C**) Detailed analysis of mutation and alteration frequency of PPP2CB and PPP2R2A in (A), PPP2R3A and PPP2R5A in (B), and PPP2R1A in (C) for each cancer type. The graphs were generated by c-bioportal.

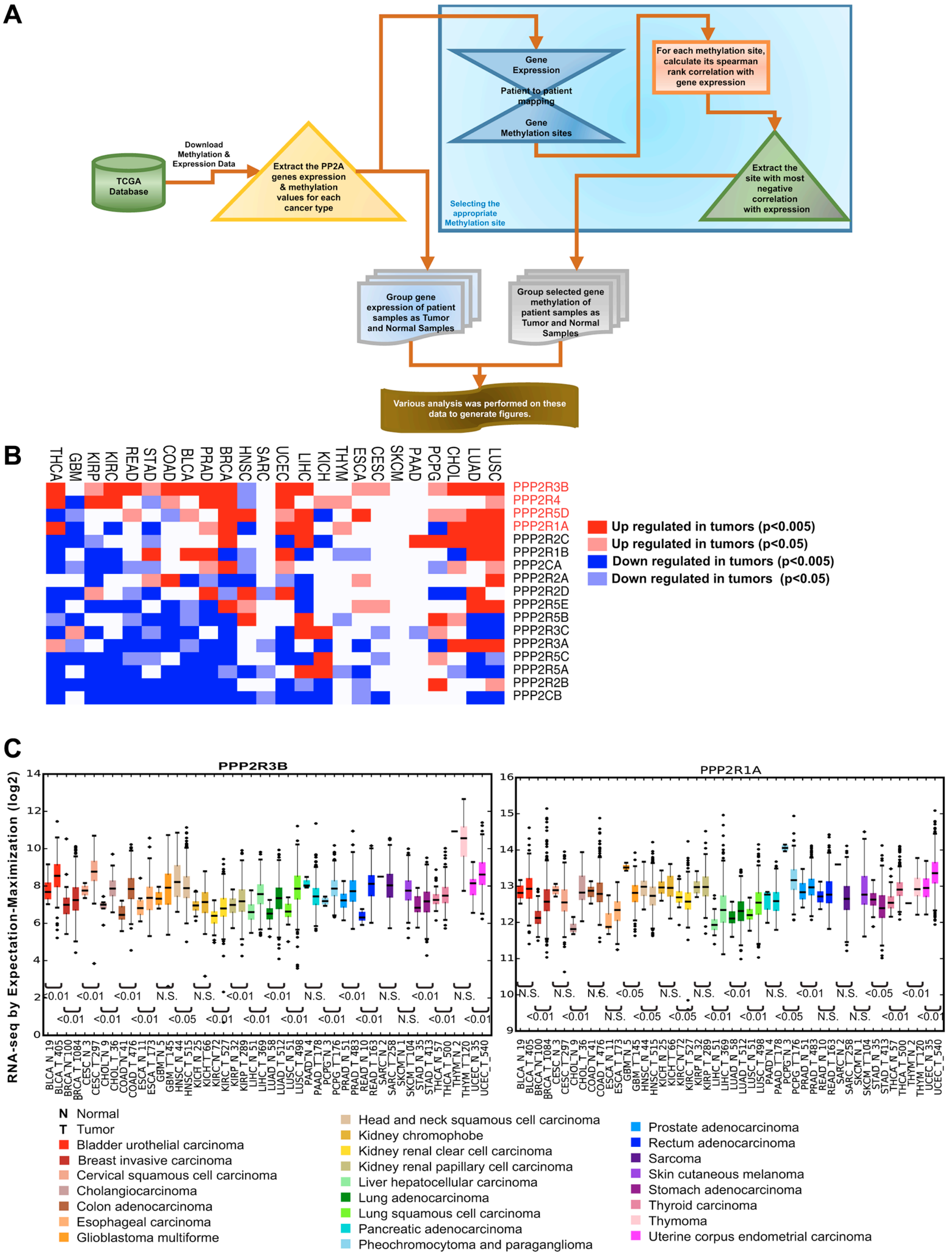

Supplementary Figure S5: Computational analysis of PP2A expression in cancer. (**A**) Workflow schematic of the methylation analyses of TCGA data. (**B**) Heat map showing the up and down regulation of each PP2A subunit in different cancer types. Blue indicates down regulation and red indicates up regulation. The top four up regulated PP2A subunits are highlighted in red. (**C**) Expression analysis of PPP2R3B and PPP2R1A in 24 cancer types and normal tissue controls using data from TCGA. The number patient samples in each cancer type is denoted by the numbers in x-axis labels. Statistical significance of the difference in expression between the normal and tumor samples are depicted for each cancer type. N.S. denotes not significant. The abbreviation of each cancer is represented as described in the TCGA portal.

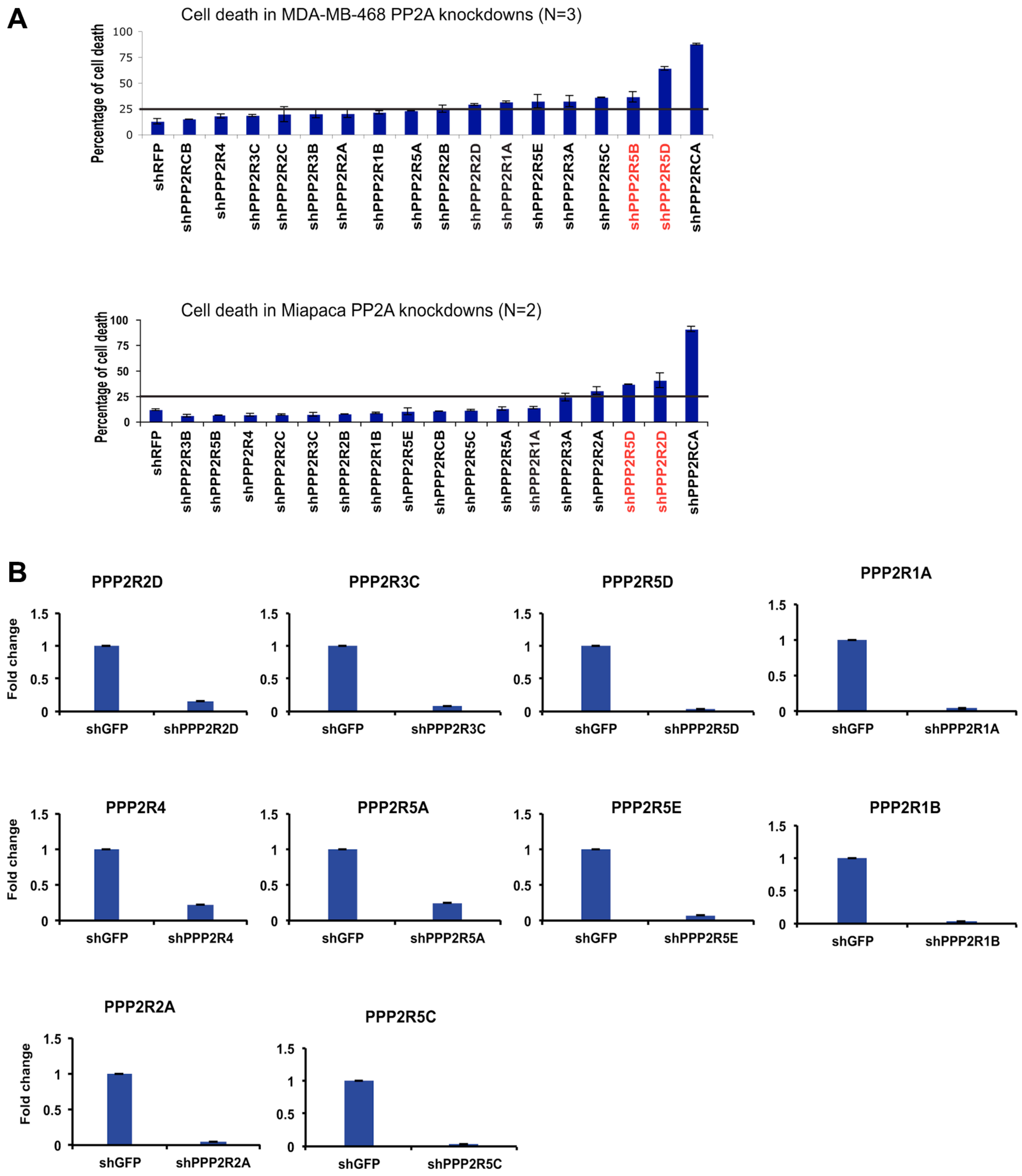

Supplementary Figure S6: PP2A knockdown efficiency and essentiality. (**A**) Bar graph quantification of the percentage of cell death as measured by 7-AAD staining for all PP2A subunit knockdowns relative to *sh*RFP in MDA-MB-468 ($N = 2$) and MiaPaCa-2 cells ($N = 2$). PP2A subunit knockdowns from Class I causing a significant increase in cell death are highlighted in red. (**B**) Bar graphs showing expression data for PP2A subunit *sh*RNA knockdown as analyzed by qPCR.
