# Supplementary material for: Therapeutic relevance of the protein phosphatase 2A in cancer
Source: Oncotarget. 2016 Aug 19;7(38):61544–61. doi: 10.18632/oncotarget.11399 (PMC5308671; doi:10.18632/oncotarget.11399)
Supplement: Supplementary file 2 [file oncotarget-07-61544-s002.docx]

Supplementary Table 3

| **SAC** | **PP2A** | **pancancer** | **Breast** | **Lung** | **Colon** | **Liver** | **Prostate** | **Ovarian** | **Cervical** | **Pancreas** | **Kidney** | **AML** |
| --- | --- | --- | --- | --- | --- | --- | --- | --- | --- | --- | --- | --- |
| BUB1 | PPP2CA | 1.00E+00 | 1.00E+00 | 1.00E+00 | 1.00E+00 | 1.00E+00 | 1.00E+00 | 3.85E-02 | 1.00E+00 | 1.00E+00 | 1.32E-01 | NaN |
| BUB1 | PPP2CB | 1.00E+00 | 1.00E+00 | 1.00E+00 | 1.00E+00 | 1.00E+00 | 1.00E+00 | 5.21E-02 | 2.06E-01 | 1.00E+00 | 1.00E+00 | NaN |
| BUB1 | PPP2R1A | 1.29E-04 | 1.61E-01 | 2.37E-05 | 1.00E+00 | 1.00E+00 | 1.00E+00 | 4.23E-02 | 2.28E-01 | 1.64E-02 | 6.31E-01 | NaN |
| BUB1 | PPP2R1B | 1.00E+00 | 1.00E+00 | 3.00E-04 | 1.00E+00 | 1.00E+00 | 1.00E+00 | 1.00E+00 | 1.00E+00 | 1.00E+00 | 1.00E+00 | NaN |
| BUB1 | PPP2R2A | 1.00E+00 | 1.00E+00 | 1.00E+00 | 4.57E-01 | 9.78E-01 | 1.00E+00 | 1.00E+00 | 1.00E+00 | 1.00E+00 | 1.00E+00 | NaN |
| BUB1 | PPP2R2B | 1.00E+00 | 1.00E+00 | 1.00E+00 | 1.00E+00 | 1.00E+00 | 1.00E+00 | 1.00E+00 | 1.00E+00 | 1.00E+00 | 4.55E-01 | NaN |
| BUB1 | PPP2R2C | 1.00E+00 | 1.00E+00 | 1.00E+00 | 1.00E+00 | 1.00E+00 | 1.00E+00 | 1.00E+00 | 1.00E+00 | 1.00E+00 | 1.00E+00 | NaN |
| BUB1 | PPP2R2D | 1.00E+00 | 1.00E+00 | 1.00E+00 | 1.00E+00 | 1.00E+00 | 1.00E+00 | 1.00E+00 | 1.00E+00 | 1.00E+00 | 1.00E+00 | NaN |
| BUB1 | PPP2R3A | 3.16E-06 | 5.22E-02 | 2.20E-05 | 1.00E+00 | 1.00E+00 | 1.21E-01 | 1.00E+00 | 1.00E+00 | 1.00E+00 | 1.00E+00 | NaN |
| BUB1 | PPP2R3B | 4.74E-04 | 1.00E+00 | 2.25E-04 | 5.81E-01 | 9.44E-01 | 1.00E+00 | 2.89E-02 | 1.00E+00 | 1.64E-02 | 1.24E-01 | NaN |
| BUB1 | PPP2R3C | 1.00E+00 | 1.00E+00 | 1.00E+00 | 1.00E+00 | 1.00E+00 | 1.00E+00 | 1.00E+00 | 1.00E+00 | 1.50E-02 | 1.00E+00 | NaN |
| BUB1 | PPP2R4 | 1.00E+00 | 1.00E+00 | 3.40E-04 | 1.00E+00 | 1.00E+00 | 1.00E+00 | 1.00E+00 | 1.00E+00 | 1.00E+00 | 1.00E+00 | NaN |
| BUB1 | PPP2R5A | 1.00E+00 | 1.00E+00 | 1.00E+00 | 1.00E+00 | 1.00E+00 | 5.74E-02 | 1.00E+00 | 1.00E+00 | 1.00E+00 | 1.00E+00 | NaN |
| BUB1 | PPP2R5B | 1.00E+00 | 1.00E+00 | 1.00E+00 | 1.00E+00 | 1.00E+00 | 1.00E+00 | 1.00E+00 | 1.00E+00 | 1.64E-02 | 1.00E+00 | NaN |
| BUB1 | PPP2R5C | 1.00E+00 | 1.00E+00 | 1.00E+00 | 1.00E+00 | 1.00E+00 | 4.88E-02 | 1.00E+00 | 2.00E-01 | 4.02E-02 | 1.00E+00 | NaN |
| BUB1 | PPP2R5D | 8.23E-12 | 1.49E-04 | 2.50E-05 | 1.00E+00 | 9.09E-01 | 1.00E+00 | 4.03E-02 | 1.35E-02 | 1.00E+00 | 1.00E+00 | NaN |
| BUB1 | PPP2R5E | 1.00E+00 | 1.00E+00 | 1.00E+00 | 1.00E+00 | 1.00E+00 | 7.86E-02 | 1.00E+00 | 1.62E-01 | 2.44E-02 | 1.00E+00 | NaN |
| BUB1 | STRN | 1.51E-12 | 5.51E-02 | 2.72E-10 | 1.94E-01 | 1.00E+00 | 3.76E-02 | 1.00E+00 | 1.39E-01 | 1.00E+00 | 1.00E+00 | NaN |
| BUB1 | STRN3 | 1.00E+00 | 1.00E+00 | 1.00E+00 | 1.00E+00 | 1.00E+00 | 3.40E-02 | 1.00E+00 | 2.01E-01 | 1.00E+00 | 1.00E+00 | NaN |
| BUB1B | PPP2CA | 1.00E+00 | 9.46E-01 | 1.00E+00 | 1.00E+00 | 8.83E-02 | 1.00E+00 | 1.94E-01 | 1.00E+00 | 2.03E-02 | 1.00E+00 | NaN |
| BUB1B | PPP2CB | 1.00E+00 | 1.00E+00 | 1.00E+00 | 1.00E+00 | 1.00E+00 | 1.00E+00 | 1.00E+00 | 1.00E+00 | 1.00E+00 | 1.00E+00 | NaN |
| BUB1B | PPP2R1A | 1.00E+00 | 1.00E+00 | 1.00E+00 | 1.00E+00 | 1.31E-01 | 1.00E+00 | 1.00E+00 | 1.00E+00 | 9.07E-03 | 7.47E-01 | NaN |
| BUB1B | PPP2R1B | 1.00E+00 | 1.00E+00 | 4.70E-02 | 1.00E+00 | 1.00E+00 | 5.01E-01 | 1.00E+00 | 1.00E+00 | 1.14E-02 | 1.00E+00 | NaN |
| BUB1B | PPP2R2A | 1.00E+00 | 1.00E+00 | 1.00E+00 | 1.10E-01 | 1.00E+00 | 1.00E+00 | 2.02E-01 | 1.00E+00 | 1.00E+00 | 1.00E+00 | NaN |
| BUB1B | PPP2R2B | 1.00E+00 | 1.00E+00 | 1.00E+00 | 1.00E+00 | 1.09E-01 | 1.00E+00 | 1.00E+00 | 1.00E+00 | 1.29E-02 | 1.00E+00 | NaN |
| BUB1B | PPP2R2C | 1.00E+00 | 1.00E+00 | 1.00E+00 | 1.00E+00 | 1.00E+00 | 1.00E+00 | 2.09E-01 | 1.00E+00 | 1.00E+00 | 1.00E+00 | NaN |
| BUB1B | PPP2R2D | 1.00E+00 | 9.66E-01 | 1.00E+00 | 1.10E-01 | 1.00E+00 | 9.17E-01 | 1.00E+00 | 1.00E+00 | 7.31E-03 | 1.00E+00 | NaN |
| BUB1B | PPP2R3A | 1.00E+00 | 1.00E+00 | 1.00E+00 | 1.00E+00 | 1.00E+00 | 9.72E-01 | 1.00E+00 | 1.00E+00 | 1.00E+00 | 1.00E+00 | NaN |
| BUB1B | PPP2R3B | 1.83E-01 | 1.00E+00 | 1.94E-02 | 8.90E-02 | 1.00E+00 | 1.00E+00 | 1.00E+00 | 1.00E+00 | 1.00E+00 | 1.00E+00 | NaN |
| BUB1B | PPP2R3C | 2.97E-01 | 9.90E-01 | 1.00E+00 | 1.00E+00 | 1.00E+00 | 1.00E+00 | 1.00E+00 | 1.00E+00 | 1.00E+00 | 7.14E-01 | NaN |
| BUB1B | PPP2R4 | 1.00E+00 | 1.00E+00 | 4.22E-02 | 1.00E+00 | 1.00E+00 | 8.97E-01 | 1.00E+00 | 1.00E+00 | 1.00E+00 | 1.00E+00 | NaN |
| BUB1B | PPP2R5A | 1.00E+00 | 1.00E+00 | 1.00E+00 | 1.00E+00 | 1.00E+00 | 1.00E+00 | 1.00E+00 | 9.96E-01 | 1.00E+00 | 6.72E-01 | NaN |
| BUB1B | PPP2R5B | 1.00E+00 | 1.00E+00 | 1.00E+00 | 1.00E+00 | 1.00E+00 | 1.00E+00 | 1.00E+00 | 1.00E+00 | 1.00E+00 | 1.00E+00 | NaN |
| BUB1B | PPP2R5C | 1.00E+00 | 1.00E+00 | 1.00E+00 | 1.00E+00 | 1.00E+00 | 1.00E+00 | 1.00E+00 | 1.00E+00 | 1.57E-02 | 7.00E-01 | NaN |
| BUB1B | PPP2R5D | 3.08E-01 | 8.32E-01 | 1.00E+00 | 1.00E+00 | 3.84E-02 | 1.00E+00 | 1.78E-01 | 1.00E+00 | 1.00E+00 | 7.22E-01 | NaN |
| BUB1B | PPP2R5E | 1.00E+00 | 1.00E+00 | 1.00E+00 | 9.49E-02 | 1.00E+00 | 1.00E+00 | 1.00E+00 | 1.00E+00 | 7.52E-03 | 7.64E-01 | NaN |
| BUB1B | STRN | 6.03E-04 | 4.28E-01 | 1.56E-02 | 9.15E-02 | 1.00E+00 | 1.00E+00 | 1.88E-01 | 9.13E-01 | 1.00E+00 | 1.00E+00 | NaN |
| BUB1B | STRN3 | 1.51E-01 | 1.00E+00 | 1.00E+00 | 1.03E-01 | 1.00E+00 | 9.73E-01 | 1.00E+00 | 9.59E-01 | 1.44E-02 | 6.65E-01 | NaN |
| BUB3 | PPP2CA | 1.00E+00 | 1.00E+00 | 1.00E+00 | 5.32E-01 | 3.26E-03 | 1.00E+00 | 1.00E+00 | 1.00E+00 | 1.00E+00 | 9.0269867e-01 1.0 | 0.00E+00 |
| BUB3 | PPP2CB | 1.00E+00 | 1.00E+00 | 1.00E+00 | 6.90E-01 | 1.00E+00 | 1.00E+00 | 1.00E+00 | 1.00E+00 | 2.72E-01 | 1.0000000e+00 4.6 | 9.90E+04 |
| BUB3 | PPP2R1A | 3.57E-02 | 2.45E-01 | 1.00E+00 | 1.00E+00 | 1.53E-03 | 1.00E+00 | 1.00E+00 | 1.00E+00 | 1.00E+00 | 9.0977801e-01 5.1 | 7.87E+04 |
| BUB3 | PPP2R1B | 1.00E+00 | 1.00E+00 | 1.00E+00 | 1.00E+00 | 1.00E+00 | 1.00E+00 | 3.81E-01 | 6.26E-02 | 1.00E+00 | 1.0000000e+00 1.0 | 0.00E+00 |
| BUB3 | PPP2R2A | 1.00E+00 | 1.00E+00 | 1.44E-01 | 3.95E-01 | 1.00E+00 | 1.00E+00 | 1.00E+00 | 1.00E+00 | 3.21E-01 | 1.0000000e+00 2.8 | 1.17E+04 |
| BUB3 | PPP2R2B | 1.00E+00 | 1.00E+00 | 1.00E+00 | 1.00E+00 | 3.26E-03 | 1.00E+00 | 1.00E+00 | 1.00E+00 | 1.00E+00 | 9.3507397e-01 1.0 | 0.00E+00 |
| BUB3 | PPP2R2C | 1.00E+00 | 1.00E+00 | 1.00E+00 | 1.00E+00 | 1.00E+00 | 1.00E+00 | 1.00E+00 | 1.00E+00 | 4.98E-01 | 9.8064475e-01 1.0 | 0.00E+00 |
| BUB3 | PPP2R2D | 2.18E-26 | 6.59E-04 | 1.80E-07 | 2.30E-02 | 1.30E-03 | 1.53E-01 | 1.73E-05 | 4.17E-02 | 1.00E+00 | 2.5263644e-01 2.1 | 3.81E+04 |
| BUB3 | PPP2R3A | 2.81E-02 | 1.00E+00 | 1.00E+00 | 1.00E+00 | 1.00E+00 | 1.00E+00 | 1.00E+00 | 8.86E-02 | 1.00E+00 | 1.0000000e+00 8.4 | 9.15E+04 |
| BUB3 | PPP2R3B | 5.18E-03 | 3.72E-01 | 1.00E+00 | 4.25E-01 | 2.35E-03 | 1.00E+00 | 6.19E-01 | 1.00E+00 | 1.00E+00 | 9.0647390e-01 7.4 | 2.43E+04 |
| BUB3 | PPP2R3C | 2.82E-02 | 1.00E+00 | 1.49E-01 | 1.00E+00 | 1.00E+00 | 1.00E+00 | 4.31E-01 | 8.86E-02 | 1.00E+00 | 1.0000000e+00 1.0 | 0.00E+00 |
| BUB3 | PPP2R4 | 1.00E+00 | 1.00E+00 | 1.00E+00 | 1.00E+00 | 1.00E+00 | 1.00E+00 | 1.00E+00 | 1.00E+00 | 2.91E-01 | 9.3730601e-01 3.9 | 5.10E+04 |
| BUB3 | PPP2R5A | 2.15E-02 | 3.79E-01 | 1.00E+00 | 1.00E+00 | 4.06E-03 | 1.00E+00 | 1.00E+00 | 1.00E+00 | 1.00E+00 | 1.0000000e+00 7.9 | 6.07E+04 |
| BUB3 | PPP2R5B | 1.00E+00 | 1.00E+00 | 1.00E+00 | 1.00E+00 | 1.00E+00 | 1.00E+00 | 1.00E+00 | 1.00E+00 | 1.00E+00 | 1.0000000e+00 1.0 | 0.00E+00 |
| BUB3 | PPP2R5C | 1.07E-03 | 1.48E-01 | 4.44E-02 | 1.00E+00 | 1.00E+00 | 1.00E+00 | 1.00E+00 | 8.46E-02 | 3.63E-01 | 1.0000000e+00 1.0 | 0.00E+00 |
| BUB3 | PPP2R5D | 6.91E-03 | 3.67E-01 | 1.37E-01 | 5.46E-01 | 1.33E-03 | 1.00E+00 | 1.00E+00 | 3.37E-02 | 1.00E+00 | 1.0000000e+00 1.0 | 0.00E+00 |
| BUB3 | PPP2R5E | 3.85E-03 | 2.24E-01 | 1.27E-01 | 1.00E+00 | 1.00E+00 | 3.96E-01 | 1.00E+00 | 9.27E-02 | 3.79E-01 | 1.0000000e+00 1.0 | 0.00E+00 |
| BUB3 | STRN | 2.92E-02 | 1.00E+00 | 1.00E+00 | 1.00E+00 | 1.00E+00 | 1.56E-01 | 1.00E+00 | 9.21E-02 | 1.00E+00 | 1.0000000e+00 7.6 | 6.26E+04 |
| BUB3 | STRN3 | 3.66E-03 | 1.00E+00 | 1.37E-01 | 1.00E+00 | 1.00E+00 | 3.72E-01 | 4.56E-01 | 8.98E-02 | 4.70E-01 | 1.0000000e+00 1.0 | 0.00E+00 |
| MAD2L1 | PPP2CA | 1.00E+00 | 1.00E+00 | 1.00E+00 | 1.00E+00 | 1.03E-01 | 1.00E+00 | 1.00E+00 | 1.00E+00 | NaN | NaN | NaN |
| MAD2L1 | PPP2CB | 1.00E+00 | 1.00E+00 | 1.00E+00 | 1.00E+00 | 1.00E+00 | 1.00E+00 | 1.00E+00 | 1.00E+00 | NaN | NaN | NaN |
| MAD2L1 | PPP2R1A | 5.97E-03 | 1.64E-01 | 7.53E-02 | 3.08E-01 | 1.00E+00 | 1.00E+00 | 8.34E-02 | 1.00E+00 | NaN | NaN | NaN |
| MAD2L1 | PPP2R1B | 1.00E+00 | 1.00E+00 | 6.79E-02 | 1.00E+00 | 1.06E-01 | 1.00E+00 | 1.00E+00 | 1.00E+00 | NaN | NaN | NaN |
| MAD2L1 | PPP2R2A | 1.00E+00 | 1.00E+00 | 1.00E+00 | 1.00E+00 | 1.00E+00 | 1.00E+00 | 8.93E-02 | 1.00E+00 | NaN | NaN | NaN |
| MAD2L1 | PPP2R2B | 1.00E+00 | 1.00E+00 | 1.00E+00 | 1.00E+00 | 1.04E-01 | 1.00E+00 | 1.00E+00 | 1.00E+00 | NaN | NaN | NaN |
| MAD2L1 | PPP2R2C | 1.00E+00 | 1.00E+00 | 1.00E+00 | 1.00E+00 | 1.00E+00 | 1.00E+00 | 1.00E+00 | 1.00E+00 | NaN | NaN | NaN |
| MAD2L1 | PPP2R2D | 1.00E+00 | 1.00E+00 | 1.00E+00 | 1.00E+00 | 1.00E+00 | 1.00E+00 | 1.00E+00 | 1.00E+00 | NaN | NaN | NaN |
| MAD2L1 | PPP2R3A | 4.44E-04 | 2.74E-01 | 1.00E+00 | 1.00E+00 | 1.10E-01 | 8.60E-01 | 1.00E+00 | 1.00E+00 | NaN | NaN | NaN |
| MAD2L1 | PPP2R3B | 2.50E-04 | 2.04E-02 | 7.46E-02 | 1.00E+00 | 1.00E+00 | 8.41E-01 | 7.66E-02 | 9.30E-01 | NaN | NaN | NaN |
| MAD2L1 | PPP2R3C | 5.92E-03 | 1.00E+00 | 1.00E+00 | 3.30E-01 | 1.00E+00 | 7.68E-01 | 9.75E-02 | 1.00E+00 | NaN | NaN | NaN |
| MAD2L1 | PPP2R4 | 1.00E+00 | 1.00E+00 | 7.49E-02 | 1.00E+00 | 1.00E+00 | 1.00E+00 | 8.33E-02 | 1.00E+00 | NaN | NaN | NaN |
| MAD2L1 | PPP2R5A | 1.00E+00 | 1.00E+00 | 1.00E+00 | 1.00E+00 | 1.00E+00 | 1.00E+00 | 7.74E-02 | 1.00E+00 | NaN | NaN | NaN |
| MAD2L1 | PPP2R5B | 1.00E+00 | 1.00E+00 | 1.00E+00 | 1.00E+00 | 1.03E-01 | 1.00E+00 | 1.00E+00 | 9.82E-01 | NaN | NaN | NaN |
| MAD2L1 | PPP2R5C | 3.55E-04 | 1.00E+00 | 1.00E+00 | 1.00E+00 | 1.00E+00 | 1.00E+00 | 1.47E-01 | 1.00E+00 | NaN | NaN | NaN |
| MAD2L1 | PPP2R5D | 4.08E-05 | 2.74E-02 | 5.00E-02 | 9.29E-02 | 1.00E+00 | 8.90E-01 | 1.00E+00 | 1.00E+00 | NaN | NaN | NaN |
| MAD2L1 | PPP2R5E | 1.00E+00 | 1.00E+00 | 1.00E+00 | 3.63E-01 | 1.00E+00 | 1.00E+00 | 8.14E-02 | 1.00E+00 | NaN | NaN | NaN |
| MAD2L1 | STRN | 5.88E-03 | 1.00E+00 | 5.10E-02 | 1.00E+00 | 1.00E+00 | 1.00E+00 | 1.00E+00 | 1.00E+00 | NaN | NaN | NaN |
| MAD2L1 | STRN3 | 4.55E-03 | 1.00E+00 | 1.00E+00 | 2.92E-01 | 1.00E+00 | 1.00E+00 | 7.96E-02 | 1.00E+00 | NaN | NaN | NaN |
| MAD1L1 | PPP2CA | 1.00E+00 | 1.00E+00 | 1.00E+00 | 1.00E+00 | 1.53E-01 | 1.00E+00 | 1.00E+00 | 1.00E+00 | 1.00E+00 | 1.00E+00 | NaN |
| MAD1L1 | PPP2CB | 1.00E+00 | 1.00E+00 | 1.00E+00 | 1.00E+00 | 1.00E+00 | 1.00E+00 | 2.69E-02 | 5.22E-01 | 1.00E+00 | 1.00E+00 | NaN |
| MAD1L1 | PPP2R1A | 1.00E+00 | 7.42E-02 | 1.00E+00 | 3.76E-02 | 1.32E-02 | 1.00E+00 | 1.00E+00 | 1.00E+00 | 6.52E-03 | 1.00E+00 | NaN |
| MAD1L1 | PPP2R1B | 1.00E+00 | 1.00E+00 | 1.80E-01 | 2.90E-02 | 1.00E+00 | 1.00E+00 | 1.00E+00 | 1.00E+00 | 1.00E+00 | 1.00E+00 | NaN |
| MAD1L1 | PPP2R2A | 1.00E+00 | 1.00E+00 | 1.00E+00 | 1.00E+00 | 1.00E+00 | 1.00E+00 | 1.00E+00 | 3.63E-01 | 1.00E+00 | 1.00E+00 | NaN |
| MAD1L1 | PPP2R2B | 1.00E+00 | 1.00E+00 | 1.00E+00 | 1.00E+00 | 1.00E+00 | 1.00E+00 | 1.00E+00 | 1.00E+00 | 1.00E+00 | 1.00E+00 | NaN |
| MAD1L1 | PPP2R2C | 1.00E+00 | 1.00E+00 | 1.00E+00 | 1.00E+00 | 1.00E+00 | 8.59E-01 | 1.00E+00 | 1.00E+00 | 1.40E-02 | 1.00E+00 | NaN |
| MAD1L1 | PPP2R2D | 1.00E+00 | 1.00E+00 | 1.00E+00 | 1.00E+00 | 4.79E-02 | 1.00E+00 | 1.00E+00 | 1.00E+00 | 1.00E+00 | 1.00E+00 | NaN |
| MAD1L1 | PPP2R3A | 7.83E-03 | 1.00E+00 | 1.00E+00 | 1.00E+00 | 1.00E+00 | 1.00E+00 | 4.53E-02 | 1.00E+00 | 1.00E+00 | 3.74E-06 | NaN |
| MAD1L1 | PPP2R3B | 5.45E-06 | 8.04E-03 | 1.00E+00 | 1.00E+00 | 1.00E+00 | 2.06E-01 | 1.00E+00 | 1.00E+00 | 1.00E+00 | 8.14E-10 | NaN |
| MAD1L1 | PPP2R3C | 1.00E+00 | 1.00E+00 | 1.00E+00 | 3.89E-02 | 1.00E+00 | 8.54E-01 | 1.00E+00 | 1.00E+00 | 1.00E+00 | 1.00E+00 | NaN |
| MAD1L1 | PPP2R4 | 1.00E+00 | 1.00E+00 | 1.80E-01 | 1.00E+00 | 1.00E+00 | 1.00E+00 | 1.00E+00 | 6.13E-01 | 1.00E+00 | 1.00E+00 | NaN |
| MAD1L1 | PPP2R5A | 1.00E+00 | 1.00E+00 | 1.00E+00 | 1.00E+00 | 1.00E+00 | 1.00E+00 | 1.00E+00 | 1.00E+00 | 1.00E+00 | 1.00E+00 | NaN |
| MAD1L1 | PPP2R5B | 1.00E+00 | 1.00E+00 | 7.98E-03 | 1.00E+00 | 1.00E+00 | 7.97E-01 | 1.00E+00 | 1.00E+00 | 1.17E-02 | 1.00E+00 | NaN |
| MAD1L1 | PPP2R5C | 1.00E+00 | 1.00E+00 | 1.00E+00 | 1.00E+00 | 1.00E+00 | 1.00E+00 | 1.00E+00 | 2.25E-01 | 1.00E+00 | 1.00E+00 | NaN |
| MAD1L1 | PPP2R5D | 1.45E-04 | 8.15E-03 | 4.10E-02 | 1.00E+00 | 5.36E-02 | 9.36E-01 | 2.96E-02 | 1.00E+00 | 1.00E+00 | 1.00E+00 | NaN |
| MAD1L1 | PPP2R5E | 1.00E+00 | 1.00E+00 | 1.79E-01 | 1.00E+00 | 1.00E+00 | 1.00E+00 | 1.00E+00 | 1.19E-01 | 1.00E+00 | 1.00E+00 | NaN |
| MAD1L1 | STRN | 1.00E+00 | 1.00E+00 | 1.00E+00 | 1.00E+00 | 1.00E+00 | 1.00E+00 | 1.00E+00 | 1.00E+00 | 1.00E+00 | 1.00E+00 | NaN |
| MAD1L1 | STRN3 | 1.00E+00 | 1.00E+00 | 7.03E-02 | 1.00E+00 | 1.00E+00 | 1.00E+00 | 1.00E+00 | 5.54E-01 | 1.00E+00 | 1.00E+00 | NaN |
| CDC20 | PPP2CA | 1.00E+00 | 1.00E+00 | 1.00E+00 | 1.00E+00 | 1.00E+00 | NaN | 1.00E+00 | 1.00E+00 | 1.00E+00 | 2.2969967e-02 1.0 | 0.00E+00 |
| CDC20 | PPP2CB | 1.00E+00 | 1.00E+00 | 1.00E+00 | 1.00E+00 | 1.00E+00 | NaN | 1.00E+00 | 1.00E+00 | 1.00E+00 | 1.0000000e+00 1.0 | 0.00E+00 |
| CDC20 | PPP2R1A | 1.00E+00 | 1.00E+00 | 2.16E-03 | 1.00E+00 | 1.00E+00 | NaN | 5.42E-01 | 2.91E-01 | 3.83E-01 | 1.0000000e+00 1.0 | 0.00E+00 |
| CDC20 | PPP2R1B | 1.00E+00 | 7.83E-02 | 2.14E-03 | 1.00E+00 | 1.00E+00 | NaN | 1.00E+00 | 1.00E+00 | 1.00E+00 | 1.0000000e+00 1.0 | 0.00E+00 |
| CDC20 | PPP2R2A | 1.00E+00 | 1.00E+00 | 1.00E+00 | 1.00E+00 | 1.00E+00 | NaN | 1.00E+00 | 1.00E+00 | 1.00E+00 | 1.0000000e+00 1.0 | 0.00E+00 |
| CDC20 | PPP2R2B | 1.00E+00 | 1.00E+00 | 1.00E+00 | 1.00E+00 | 1.00E+00 | NaN | 1.00E+00 | 2.41E-01 | 1.00E+00 | 1.0000000e+00 1.0 | 0.00E+00 |
| CDC20 | PPP2R2C | 1.00E+00 | 1.00E+00 | 1.15E-02 | 1.00E+00 | 1.00E+00 | NaN | 1.00E+00 | 1.00E+00 | 1.00E+00 | 2.0358461e-03 1.0 | 0.00E+00 |
| CDC20 | PPP2R2D | 1.00E+00 | 1.00E+00 | 1.00E+00 | 1.00E+00 | 1.00E+00 | NaN | 1.00E+00 | 1.00E+00 | 1.00E+00 | 1.0000000e+00 1.0 | 0.00E+00 |
| CDC20 | PPP2R3A | 4.28E-06 | 3.36E-04 | 1.00E+00 | 1.00E+00 | 1.00E+00 | NaN | 1.00E+00 | 2.97E-01 | 3.69E-01 | 1.0000000e+00 1.0 | 0.00E+00 |
| CDC20 | PPP2R3B | 1.24E-05 | 9.80E-02 | 2.78E-03 | 1.00E+00 | 1.00E+00 | NaN | 5.30E-01 | 2.02E-01 | 1.00E+00 | 3.0710460e-03 1.0 | 0.00E+00 |
| CDC20 | PPP2R3C | 1.00E+00 | 1.00E+00 | 2.07E-03 | 9.18E-02 | 1.00E+00 | NaN | 5.59E-01 | 2.40E-01 | 5.10E-01 | 1.0000000e+00 1.0 | 0.00E+00 |
| CDC20 | PPP2R4 | 1.00E+00 | 1.00E+00 | 1.00E+00 | 4.59E-02 | 1.00E+00 | NaN | 1.00E+00 | 1.00E+00 | 4.09E-01 | 1.0000000e+00 1.0 | 0.00E+00 |
| CDC20 | PPP2R5A | 1.00E+00 | 1.00E+00 | 1.00E+00 | 1.00E+00 | 2.09E-02 | NaN | 1.00E+00 | 2.29E-01 | 1.00E+00 | 1.0000000e+00 1.0 | 0.00E+00 |
| CDC20 | PPP2R5B | 1.00E+00 | 3.84E-02 | 2.29E-03 | 1.00E+00 | 1.00E+00 | NaN | 1.00E+00 | 1.00E+00 | 1.00E+00 | 1.0000000e+00 1.0 | 0.00E+00 |
| CDC20 | PPP2R5C | 3.18E-04 | 1.00E+00 | 1.00E+00 | 6.29E-02 | 1.00E+00 | NaN | 5.17E-01 | 2.20E-01 | 2.22E-01 | 1.0000000e+00 1.0 | 0.00E+00 |
| CDC20 | PPP2R5D | 1.96E-15 | 2.00E-06 | 2.95E-03 | 5.99E-02 | 2.22E-02 | NaN | 1.00E+00 | 2.07E-01 | 1.00E+00 | 1.0000000e+00 1.1 | 1.34E+01 |
| CDC20 | PPP2R5E | 1.00E+00 | 1.00E+00 | 2.37E-03 | 5.98E-02 | 1.00E+00 | NaN | 6.51E-01 | 1.00E+00 | 5.26E-01 | 1.0000000e+00 1.0 | 0.00E+00 |
| CDC20 | STRN | 1.00E+00 | 1.00E+00 | 1.00E+00 | 1.00E+00 | 1.00E+00 | NaN | 6.61E-01 | 1.00E+00 | 1.00E+00 | 1.0000000e+00 1.0 | 0.00E+00 |
| CDC20 | STRN3 | 1.00E+00 | 1.00E+00 | 4.81E-03 | 1.00E+00 | 1.00E+00 | NaN | 6.65E-01 | 1.00E+00 | 1.00E+00 | 1.0000000e+00 1.0 | 0.00E+00 |
| CENPE | PPP2CA | 1.00E+00 | 1.00E+00 | 1.00E+00 | 9.21E-01 | NaN | 8.94E-01 | 1.00E+00 | 1.00E+00 | NaN | 5.61E-01 | NaN |
| CENPE | PPP2CB | 1.00E+00 | 1.00E+00 | 1.00E+00 | 1.00E+00 | NaN | 1.00E+00 | 1.00E+00 | 1.00E+00 | NaN | 1.00E+00 | NaN |
| CENPE | PPP2R1A | 1.00E+00 | 7.64E-02 | 1.00E+00 | 1.00E+00 | NaN | 1.00E+00 | 1.00E+00 | 1.00E+00 | NaN | 1.00E+00 | NaN |
| CENPE | PPP2R1B | 1.00E+00 | 1.00E+00 | 1.00E+00 | 1.00E+00 | NaN | 9.24E-01 | 1.19E-02 | 1.00E+00 | NaN | 1.00E+00 | NaN |
| CENPE | PPP2R2A | 1.00E+00 | 1.00E+00 | 1.00E+00 | 1.00E+00 | NaN | 1.00E+00 | 1.27E-02 | 1.00E+00 | NaN | 1.00E+00 | NaN |
| CENPE | PPP2R2B | 1.00E+00 | 1.00E+00 | 1.00E+00 | 1.00E+00 | NaN | 1.00E+00 | 1.00E+00 | 1.00E+00 | NaN | 3.63E-01 | NaN |
| CENPE | PPP2R2C | 1.00E+00 | 1.00E+00 | 1.00E+00 | 9.49E-01 | NaN | 1.00E+00 | 1.00E+00 | 1.00E+00 | NaN | 1.00E+00 | NaN |
| CENPE | PPP2R2D | 1.00E+00 | 1.00E+00 | 1.00E+00 | 1.00E+00 | NaN | 1.00E+00 | 1.00E+00 | 7.15E-01 | NaN | 1.00E+00 | NaN |
| CENPE | PPP2R3A | 2.96E-01 | 7.32E-02 | 3.72E-01 | 1.00E+00 | NaN | 1.00E+00 | 1.23E-02 | 6.62E-01 | NaN | 1.00E+00 | NaN |
| CENPE | PPP2R3B | 7.86E-01 | 5.42E-02 | 1.00E+00 | 4.23E-01 | NaN | 9.21E-01 | 1.14E-02 | 1.00E+00 | NaN | 4.86E-01 | NaN |
| CENPE | PPP2R3C | 5.98E-01 | 3.19E-02 | 4.46E-01 | 9.08E-01 | NaN | 1.00E+00 | 8.17E-03 | 6.56E-01 | NaN | 1.00E+00 | NaN |
| CENPE | PPP2R4 | 4.13E-01 | 7.09E-02 | 1.00E+00 | 8.54E-01 | NaN | 1.00E+00 | 1.11E-02 | 1.00E+00 | NaN | 1.00E+00 | NaN |
| CENPE | PPP2R5A | 1.00E+00 | 1.00E+00 | 1.00E+00 | 1.00E+00 | NaN | 1.00E+00 | 1.24E-02 | 5.89E-01 | NaN | 1.00E+00 | NaN |
| CENPE | PPP2R5B | 1.00E+00 | 1.00E+00 | 3.66E-01 | 1.00E+00 | NaN | 1.00E+00 | 1.09E-02 | 1.00E+00 | NaN | 5.56E-01 | NaN |
| CENPE | PPP2R5C | 7.44E-01 | 1.00E+00 | 2.85E-01 | 1.00E+00 | NaN | 1.00E+00 | 1.00E+00 | 1.00E+00 | NaN | 1.00E+00 | NaN |
| CENPE | PPP2R5D | 7.19E-02 | 3.58E-02 | 3.82E-01 | 8.20E-01 | NaN | 1.00E+00 | 1.00E+00 | 1.00E+00 | NaN | 1.00E+00 | NaN |
| CENPE | PPP2R5E | 7.05E-01 | 1.00E+00 | 3.62E-01 | 9.10E-01 | NaN | 1.00E+00 | 1.00E+00 | 9.02E-01 | NaN | 1.00E+00 | NaN |
| CENPE | STRN | 6.88E-01 | 2.85E-02 | 4.34E-01 | 8.65E-01 | NaN | 9.63E-01 | 1.00E+00 | 5.98E-01 | NaN | 1.00E+00 | NaN |
| CENPE | STRN3 | 6.44E-02 | 2.49E-02 | 4.30E-01 | 8.47E-01 | NaN | 1.00E+00 | 1.10E-02 | 5.58E-01 | NaN | 1.00E+00 | NaN |
| ZW10 | PPP2CA | 1.00E+00 | 1.00E+00 | 1.00E+00 | 1.00E+00 | 6.07E-02 | 1.00E+00 | 2.48E-01 | 1.00E+00 | 6.31E-01 | 2.7545823e-01 1.0 | 0.00E+00 |
| ZW10 | PPP2CB | 1.00E+00 | 1.00E+00 | 1.00E+00 | 1.00E+00 | 1.00E+00 | 1.00E+00 | 1.00E+00 | 1.00E+00 | 1.00E+00 | 1.0000000e+00 1.0 | 0.00E+00 |
| ZW10 | PPP2R1A | 1.00E+00 | 1.00E+00 | 2.80E-03 | 3.42E-01 | 1.00E+00 | 1.00E+00 | 2.47E-01 | 1.04E-03 | 1.00E+00 | 1.0000000e+00 1.0 | 0.00E+00 |
| ZW10 | PPP2R1B | 3.79E-40 | 3.20E-08 | 9.91E-20 | 7.83E-03 | 2.34E-02 | 4.41E-01 | 2.92E-06 | 2.66E-03 | 4.07E-01 | 1.4381341e-02 7.1 | 5.07E+02 |
| ZW10 | PPP2R2A | 1.00E+00 | 1.00E+00 | 1.00E+00 | 1.00E+00 | 2.19E-02 | 1.00E+00 | 1.00E+00 | 1.00E+00 | 1.00E+00 | 1.0000000e+00 1.0 | 0.00E+00 |
| ZW10 | PPP2R2B | 1.00E+00 | 1.00E+00 | 1.00E+00 | 1.00E+00 | 1.00E+00 | 1.00E+00 | 2.31E-01 | 1.00E+00 | 1.00E+00 | 2.7622194e-01 1.0 | 0.00E+00 |
| ZW10 | PPP2R2C | 1.00E+00 | 1.00E+00 | 1.00E+00 | 1.00E+00 | 1.00E+00 | 1.00E+00 | 2.44E-01 | 1.00E+00 | 9.54E-01 | 1.0000000e+00 4.6 | 1.36E+04 |
| ZW10 | PPP2R2D | 1.00E+00 | 1.00E+00 | 1.00E+00 | 1.00E+00 | 1.00E+00 | 1.00E+00 | 1.00E+00 | 1.00E+00 | 1.00E+00 | 1.0000000e+00 1.0 | 0.00E+00 |
| ZW10 | PPP2R3A | 5.63E-06 | 8.86E-02 | 1.80E-03 | 1.00E+00 | 1.00E+00 | 4.45E-01 | 1.00E+00 | 1.00E+00 | 1.00E+00 | 1.0000000e+00 3.0 | 1.91E+04 |
| ZW10 | PPP2R3B | 1.00E+00 | 1.00E+00 | 1.00E+00 | 2.37E-01 | 1.00E+00 | 1.00E+00 | 1.00E+00 | 1.00E+00 | 9.80E-01 | 2.4412427e-01 1.0 | 0.00E+00 |
| ZW10 | PPP2R3C | 1.66E-03 | 1.00E+00 | 2.01E-03 | 1.00E+00 | 1.00E+00 | 1.00E+00 | 2.29E-01 | 1.00E+00 | 1.00E+00 | 3.9975418e-02 1.0 | 0.00E+00 |
| ZW10 | PPP2R4 | 1.00E+00 | 1.00E+00 | 1.00E+00 | 1.00E+00 | 2.57E-02 | 1.00E+00 | 2.32E-01 | 1.23E-03 | 9.94E-01 | 1.0000000e+00 1.0 | 0.00E+00 |
| ZW10 | PPP2R5A | 9.89E-03 | 1.67E-01 | 1.00E+00 | 1.00E+00 | 1.97E-02 | 1.00E+00 | 1.00E+00 | 1.00E+00 | 1.00E+00 | 1.0000000e+00 1.0 | 0.00E+00 |
| ZW10 | PPP2R5B | 1.00E+00 | 2.99E-02 | 1.41E-03 | 1.00E+00 | 2.40E-02 | 5.51E-01 | 2.42E-01 | 1.27E-03 | 1.00E+00 | 1.0000000e+00 1.7 | 5.39E+04 |
| ZW10 | PPP2R5C | 1.00E+00 | 1.00E+00 | 1.34E-04 | 1.00E+00 | 1.00E+00 | 1.00E+00 | 1.00E+00 | 1.00E+00 | 1.00E+00 | 2.9115975e-02 1.0 | 0.00E+00 |
| ZW10 | PPP2R5D | 6.24E-08 | 5.54E-05 | 2.38E-03 | 1.00E+00 | 2.54E-02 | 1.00E+00 | 1.00E+00 | 1.00E+00 | 9.80E-01 | 1.0000000e+00 1.0 | 0.00E+00 |
| ZW10 | PPP2R5E | 1.00E+00 | 1.00E+00 | 1.00E+00 | 1.00E+00 | 1.00E+00 | 6.45E-01 | 1.00E+00 | 1.00E+00 | 1.00E+00 | 4.3610501e-02 1.0 | 0.00E+00 |
| ZW10 | STRN | 9.00E-06 | 3.42E-02 | 2.21E-04 | 1.00E+00 | 1.00E+00 | 1.00E+00 | 1.00E+00 | 1.18E-03 | 4.77E-01 | 1.0000000e+00 1.0 | 0.00E+00 |
| ZW10 | STRN3 | 1.00E+00 | 1.00E+00 | 2.43E-03 | 1.00E+00 | 1.00E+00 | 1.00E+00 | 1.00E+00 | 1.00E+00 | 1.00E+00 | 4.3864280e-02 1.0 | 0.00E+00 |
| ZWILCH | PPP2CA | 1.00E+00 | 1.00E+00 | 1.00E+00 | 1.00E+00 | 4.07E-01 | 1.00E+00 | 1.00E+00 | 1.00E+00 | 9.62E-01 | 1.00E+00 | NaN |
| ZWILCH | PPP2CB | 1.00E+00 | 1.00E+00 | 1.00E+00 | 1.00E+00 | 1.00E+00 | 1.00E+00 | 1.00E+00 | 8.45E-01 | 1.00E+00 | 1.00E+00 | NaN |
| ZWILCH | PPP2R1A | 1.00E+00 | 1.00E+00 | 1.32E-03 | 1.00E+00 | 1.00E+00 | 1.00E+00 | 1.00E+00 | 9.74E-01 | 1.00E+00 | 1.00E+00 | NaN |
| ZWILCH | PPP2R1B | 1.00E+00 | 1.00E+00 | 9.19E-03 | 1.00E+00 | 1.00E+00 | 1.00E+00 | 1.00E+00 | 8.80E-01 | 1.00E+00 | 1.00E+00 | NaN |
| ZWILCH | PPP2R2A | 1.00E+00 | 1.00E+00 | 1.00E+00 | 1.00E+00 | 1.00E+00 | 1.00E+00 | 1.00E+00 | 1.00E+00 | 1.00E+00 | 1.00E+00 | NaN |
| ZWILCH | PPP2R2B | 1.00E+00 | 1.00E+00 | 1.00E+00 | 1.00E+00 | 4.41E-01 | 1.00E+00 | 1.00E+00 | 1.00E+00 | 1.00E+00 | 1.00E+00 | NaN |
| ZWILCH | PPP2R2C | 1.00E+00 | 1.00E+00 | 1.00E+00 | 1.39E-02 | 5.68E-01 | 8.52E-03 | 1.00E+00 | 1.00E+00 | 1.00E+00 | 1.00E+00 | NaN |
| ZWILCH | PPP2R2D | 1.00E+00 | 1.00E+00 | 1.00E+00 | 1.31E-02 | 1.00E+00 | 1.00E+00 | 1.00E+00 | 1.00E+00 | 1.00E+00 | 1.00E+00 | NaN |
| ZWILCH | PPP2R3A | 1.05E-02 | 2.14E-02 | 1.00E+00 | 1.00E+00 | 1.00E+00 | 1.00E+00 | 1.00E+00 | 1.00E+00 | 1.00E+00 | 1.00E+00 | NaN |
| ZWILCH | PPP2R3B | 1.00E+00 | 1.00E+00 | 1.00E+00 | 1.00E+00 | 1.00E+00 | 1.00E+00 | 1.00E+00 | 1.00E+00 | 1.00E+00 | 1.00E+00 | NaN |
| ZWILCH | PPP2R3C | 1.00E+00 | 1.00E+00 | 1.00E+00 | 1.00E+00 | 2.97E-01 | 1.20E-02 | 1.00E+00 | 1.00E+00 | 1.00E+00 | 1.00E+00 | NaN |
| ZWILCH | PPP2R4 | 1.00E+00 | 1.00E+00 | 1.00E+00 | 1.00E+00 | 1.00E+00 | 1.00E+00 | 1.00E+00 | 1.00E+00 | 1.00E+00 | 4.82E-02 | NaN |
| ZWILCH | PPP2R5A | 1.00E+00 | 1.00E+00 | 1.00E+00 | 1.00E+00 | 1.00E+00 | 1.00E+00 | 1.00E+00 | 1.00E+00 | 1.00E+00 | 1.00E+00 | NaN |
| ZWILCH | PPP2R5B | 1.00E+00 | 1.00E+00 | 1.00E+00 | 1.00E+00 | 1.00E+00 | 1.00E+00 | 1.00E+00 | 1.00E+00 | 1.00E+00 | 1.00E+00 | NaN |
| ZWILCH | PPP2R5C | 1.00E+00 | 1.71E-02 | 1.00E+00 | 1.00E+00 | 1.00E+00 | 1.07E-02 | 1.00E+00 | 8.89E-01 | 9.09E-01 | 1.00E+00 | NaN |
| ZWILCH | PPP2R5D | 5.73E-03 | 1.33E-02 | 1.00E+00 | 1.00E+00 | 5.17E-01 | 1.00E+00 | 1.00E+00 | 1.00E+00 | 1.00E+00 | 1.00E+00 | NaN |
| ZWILCH | PPP2R5E | 1.00E+00 | 1.00E+00 | 1.00E+00 | 1.22E-02 | 1.00E+00 | 1.00E+00 | 1.00E+00 | 1.00E+00 | 5.82E-01 | 1.00E+00 | NaN |
| ZWILCH | STRN | 1.50E-04 | 1.00E+00 | 3.49E-03 | 6.87E-03 | 4.93E-01 | 1.00E+00 | 1.00E+00 | 1.00E+00 | 9.04E-01 | 1.00E+00 | NaN |
| ZWILCH | STRN3 | 1.00E+00 | 1.00E+00 | 1.00E+00 | 1.21E-02 | 4.34E-01 | 1.00E+00 | 1.00E+00 | 1.00E+00 | 1.00E+00 | 1.00E+00 | NaN |
| KNTC1 | PPP2CA | 1.00E+00 | 8.75E-02 | 1.00E+00 | 1.00E+00 | 1.00E+00 | 8.25E-01 | 1.88E-01 | 1.00E+00 | 2.48E-02 | 1.00E+00 | NaN |
| KNTC1 | PPP2CB | 1.00E+00 | 1.00E+00 | 1.00E+00 | 1.00E+00 | 1.00E+00 | 1.00E+00 | 1.00E+00 | 1.00E+00 | 1.00E+00 | 1.00E+00 | NaN |
| KNTC1 | PPP2R1A | 1.00E+00 | 1.00E+00 | 1.00E+00 | 1.00E+00 | 1.00E+00 | 1.00E+00 | 1.00E+00 | 1.00E+00 | 1.73E-02 | 1.00E+00 | NaN |
| KNTC1 | PPP2R1B | 1.00E+00 | 1.00E+00 | 3.45E-01 | 9.99E-01 | 1.00E+00 | 1.00E+00 | 1.00E+00 | 4.35E-02 | 1.50E-02 | 1.00E+00 | NaN |
| KNTC1 | PPP2R2A | 1.00E+00 | 1.00E+00 | 1.00E+00 | 1.00E+00 | 1.00E+00 | 1.00E+00 | 1.00E+00 | 1.00E+00 | 1.00E+00 | 1.00E+00 | NaN |
| KNTC1 | PPP2R2B | 1.00E+00 | 1.00E+00 | 1.00E+00 | 1.00E+00 | 9.17E-03 | 1.00E+00 | 1.00E+00 | 1.00E+00 | 2.00E-02 | 1.00E+00 | NaN |
| KNTC1 | PPP2R2C | 1.00E+00 | 1.00E+00 | 1.00E+00 | 9.10E-01 | 1.00E+00 | 1.00E+00 | 1.00E+00 | 1.00E+00 | 1.00E+00 | 1.00E+00 | NaN |
| KNTC1 | PPP2R2D | 1.00E+00 | 1.00E+00 | 1.00E+00 | 1.00E+00 | 1.00E+00 | 1.00E+00 | 1.00E+00 | 1.00E+00 | 1.40E-02 | 1.00E+00 | NaN |
| KNTC1 | PPP2R3A | 7.38E-03 | 1.00E+00 | 1.00E+00 | 1.00E+00 | 1.00E+00 | 1.00E+00 | 1.00E+00 | 5.31E-02 | 1.00E+00 | 1.00E+00 | NaN |
| KNTC1 | PPP2R3B | 7.90E-05 | 9.21E-02 | 7.94E-03 | 9.64E-01 | 1.00E+00 | 1.00E+00 | 2.77E-01 | 4.14E-02 | 1.00E+00 | 3.56E-04 | NaN |
| KNTC1 | PPP2R3C | 1.00E+00 | 1.00E+00 | 1.00E+00 | 1.00E+00 | 1.00E+00 | 1.00E+00 | 1.00E+00 | 1.00E+00 | 1.00E+00 | 1.00E+00 | NaN |
| KNTC1 | PPP2R4 | 1.00E+00 | 1.00E+00 | 1.00E+00 | 1.00E+00 | 1.00E+00 | 1.00E+00 | 1.00E+00 | 1.00E+00 | 1.00E+00 | 1.00E+00 | NaN |
| KNTC1 | PPP2R5A | 1.00E+00 | 1.00E+00 | 1.00E+00 | 1.00E+00 | 7.24E-03 | 1.00E+00 | 1.00E+00 | 1.00E+00 | 1.00E+00 | 1.00E+00 | NaN |
| KNTC1 | PPP2R5B | 1.00E+00 | 1.00E+00 | 2.31E-01 | 1.00E+00 | 1.00E+00 | 1.00E+00 | 1.00E+00 | 1.00E+00 | 1.00E+00 | 1.00E+00 | NaN |
| KNTC1 | PPP2R5C | 1.00E+00 | 1.00E+00 | 1.00E+00 | 1.00E+00 | 1.00E+00 | 1.00E+00 | 1.00E+00 | 1.00E+00 | 1.58E-02 | 1.00E+00 | NaN |
| KNTC1 | PPP2R5D | 6.21E-03 | 1.43E-02 | 1.00E+00 | 1.00E+00 | 6.38E-03 | 1.00E+00 | 1.00E+00 | 1.00E+00 | 1.00E+00 | 1.00E+00 | NaN |
| KNTC1 | PPP2R5E | 1.00E+00 | 1.00E+00 | 1.00E+00 | 1.00E+00 | 1.00E+00 | 1.00E+00 | 1.93E-01 | 1.00E+00 | 1.29E-02 | 1.00E+00 | NaN |
| KNTC1 | STRN | 1.77E-04 | 1.76E-01 | 3.62E-01 | 1.00E+00 | 9.15E-03 | 9.98E-01 | 1.03E-01 | 1.00E+00 | 1.00E+00 | 1.00E+00 | NaN |
| KNTC1 | STRN3 | 1.00E+00 | 1.00E+00 | 1.00E+00 | 1.00E+00 | 1.00E+00 | 1.00E+00 | 1.00E+00 | 1.00E+00 | 2.35E-02 | 1.00E+00 | NaN |
| TTK | PPP2CA | 1.00E+00 | 1.00E+00 | 1.00E+00 | 1.00E+00 | 3.06E-02 | 1.25E-01 | 1.00E+00 | 1.00E+00 | 1.00E+00 | 4.11E-01 | NaN |
| TTK | PPP2CB | 1.00E+00 | 1.00E+00 | 1.00E+00 | 1.00E+00 | 1.00E+00 | 3.37E-01 | 1.00E+00 | 4.90E-03 | 1.00E+00 | 1.00E+00 | NaN |
| TTK | PPP2R1A | 1.00E+00 | 2.03E-02 | 1.00E+00 | 1.00E+00 | 1.00E+00 | 1.00E+00 | 1.00E+00 | 1.00E+00 | 1.00E+00 | 5.02E-01 | NaN |
| TTK | PPP2R1B | 1.00E+00 | 1.41E-03 | 1.00E+00 | 1.00E+00 | 1.00E+00 | 1.00E+00 | 1.00E+00 | 1.00E+00 | 1.00E+00 | 1.00E+00 | NaN |
| TTK | PPP2R2A | 1.00E+00 | 1.00E+00 | 1.00E+00 | 1.00E+00 | 1.00E+00 | 1.00E+00 | 1.00E+00 | 1.00E+00 | 1.00E+00 | 1.00E+00 | NaN |
| TTK | PPP2R2B | 1.00E+00 | 1.00E+00 | 1.00E+00 | 1.00E+00 | 3.54E-02 | 4.39E-01 | 1.00E+00 | 1.00E+00 | 1.00E+00 | 1.00E+00 | NaN |
| TTK | PPP2R2C | 1.00E+00 | 1.00E+00 | 1.00E+00 | 1.00E+00 | 1.00E+00 | 7.32E-02 | 1.00E+00 | 1.00E+00 | 9.36E-02 | 4.61E-01 | NaN |
| TTK | PPP2R2D | 1.00E+00 | 1.00E+00 | 1.00E+00 | 2.79E-03 | 1.00E+00 | 5.83E-01 | 2.57E-02 | 1.00E+00 | 1.00E+00 | 1.00E+00 | NaN |
| TTK | PPP2R3A | 1.87E-05 | 8.90E-06 | 3.41E-03 | 1.00E+00 | 1.00E+00 | 1.00E+00 | 2.60E-02 | 1.00E+00 | 1.26E-01 | 1.00E+00 | NaN |
| TTK | PPP2R3B | 1.00E+00 | 1.00E+00 | 1.00E+00 | 3.93E-03 | 3.21E-02 | 1.00E+00 | 1.55E-02 | 1.00E+00 | 1.00E+00 | 5.07E-01 | NaN |
| TTK | PPP2R3C | 1.00E+00 | 1.00E+00 | 1.00E+00 | 1.00E+00 | 1.00E+00 | 1.00E+00 | 2.18E-02 | 1.00E+00 | 1.00E+00 | 1.00E+00 | NaN |
| TTK | PPP2R4 | 1.00E+00 | 1.00E+00 | 1.21E-02 | 3.13E-03 | 1.00E+00 | 5.88E-01 | 1.00E+00 | 1.00E+00 | 1.00E+00 | 1.00E+00 | NaN |
| TTK | PPP2R5A | 1.00E+00 | 1.00E+00 | 1.00E+00 | 1.00E+00 | 3.03E-02 | 1.00E+00 | 1.00E+00 | 5.04E-03 | 1.00E+00 | 4.19E-01 | NaN |
| TTK | PPP2R5B | 1.00E+00 | 1.00E+00 | 1.02E-02 | 1.00E+00 | 1.00E+00 | 1.00E+00 | 1.00E+00 | 1.00E+00 | 1.10E-01 | 1.00E+00 | NaN |
| TTK | PPP2R5C | 1.00E+00 | 1.00E+00 | 1.00E+00 | 1.00E+00 | 1.00E+00 | 5.35E-01 | 1.49E-02 | 1.00E+00 | 1.00E+00 | 1.00E+00 | NaN |
| TTK | PPP2R5D | 2.34E-30 | 5.84E-14 | 5.16E-04 | 1.13E-06 | 4.02E-03 | 1.00E+00 | 1.64E-02 | 4.28E-03 | 1.01E-01 | 3.85E-01 | NaN |
| TTK | PPP2R5E | 1.00E+00 | 1.00E+00 | 1.00E+00 | 1.00E+00 | 1.00E+00 | 1.00E+00 | 1.00E+00 | 1.00E+00 | 1.00E+00 | 1.00E+00 | NaN |
| TTK | STRN | 2.14E-06 | 5.28E-03 | 4.23E-03 | 1.00E+00 | 3.40E-02 | 1.00E+00 | 1.00E+00 | 4.64E-03 | 1.00E+00 | 1.00E+00 | NaN |
| TTK | STRN3 | 1.00E+00 | 1.00E+00 | 1.00E+00 | 1.00E+00 | 1.00E+00 | 1.00E+00 | 1.45E-02 | 1.00E+00 | 1.00E+00 | 1.00E+00 | NaN |
| PLK1 | PPP2CA | 1.00E+00 | 1.00E+00 | 1.00E+00 | 1.00E+00 | 1.00E+00 | 1.00E+00 | 4.57E-01 | 1.00E+00 | 1.00E+00 | 3.02E-02 | NaN |
| PLK1 | PPP2CB | 1.00E+00 | 1.00E+00 | 1.00E+00 | 1.00E+00 | 3.25E-01 | 1.00E+00 | 1.00E+00 | 1.00E+00 | 1.00E+00 | 1.00E+00 | NaN |
| PLK1 | PPP2R1A | 7.25E-03 | 1.15E-01 | 3.51E-02 | 1.00E+00 | 5.21E-01 | 1.00E+00 | 2.34E-01 | 1.00E+00 | 1.00E+00 | 1.00E+00 | NaN |
| PLK1 | PPP2R1B | 1.00E+00 | 1.00E+00 | 4.84E-02 | 1.00E+00 | 1.00E+00 | 2.78E-02 | 1.00E+00 | 5.96E-02 | 1.00E+00 | 1.00E+00 | NaN |
| PLK1 | PPP2R2A | 1.00E+00 | 1.00E+00 | 6.73E-02 | 1.00E+00 | 2.00E-01 | 1.00E+00 | 1.00E+00 | 1.00E+00 | 1.00E+00 | 1.00E+00 | NaN |
| PLK1 | PPP2R2B | 1.00E+00 | 1.00E+00 | 1.00E+00 | 1.00E+00 | 1.00E+00 | 1.00E+00 | 1.00E+00 | 1.00E+00 | 1.00E+00 | 1.00E+00 | NaN |
| PLK1 | PPP2R2C | 1.00E+00 | 1.00E+00 | 1.00E+00 | 1.00E+00 | 1.00E+00 | 1.00E+00 | 1.00E+00 | 1.00E+00 | 1.00E+00 | 1.00E+00 | NaN |
| PLK1 | PPP2R2D | 1.00E+00 | 1.00E+00 | 1.00E+00 | 1.00E+00 | 1.00E+00 | 1.00E+00 | 1.00E+00 | 1.00E+00 | 1.00E+00 | 1.00E+00 | NaN |
| PLK1 | PPP2R3A | 6.23E-03 | 2.14E-02 | 6.75E-02 | 1.00E+00 | 1.00E+00 | 2.40E-02 | 1.00E+00 | 1.00E+00 | 1.00E+00 | 1.00E+00 | NaN |
| PLK1 | PPP2R3B | 1.48E-04 | 1.63E-02 | 4.26E-02 | 5.14E-01 | 1.00E+00 | 2.29E-02 | 4.20E-01 | 5.62E-02 | 1.00E+00 | 9.80E-03 | NaN |
| PLK1 | PPP2R3C | 1.00E+00 | 1.00E+00 | 1.00E+00 | 1.00E+00 | 1.00E+00 | 1.00E+00 | 4.38E-01 | 1.00E+00 | 1.00E+00 | 1.00E+00 | NaN |
| PLK1 | PPP2R4 | 1.00E+00 | 1.00E+00 | 1.00E+00 | 6.41E-01 | 3.32E-01 | 1.00E+00 | 4.38E-01 | 1.00E+00 | 1.20E-01 | 1.00E+00 | NaN |
| PLK1 | PPP2R5A | 1.00E+00 | 1.00E+00 | 1.00E+00 | 1.00E+00 | 1.00E+00 | 1.00E+00 | 1.00E+00 | 5.33E-02 | 1.02E-01 | 1.00E+00 | NaN |
| PLK1 | PPP2R5B | 1.00E+00 | 1.00E+00 | 1.00E+00 | 1.00E+00 | 1.00E+00 | 2.21E-02 | 4.72E-01 | 1.00E+00 | 1.00E+00 | 2.82E-02 | NaN |
| PLK1 | PPP2R5C | 1.00E+00 | 1.00E+00 | 4.54E-02 | 1.00E+00 | 1.00E+00 | 2.30E-02 | 4.16E-01 | 1.00E+00 | 1.00E+00 | 1.00E+00 | NaN |
| PLK1 | PPP2R5D | 6.90E-08 | 2.53E-04 | 1.00E+00 | 6.02E-01 | 1.00E+00 | 1.00E+00 | 3.29E-01 | 5.12E-02 | 1.00E+00 | 1.00E+00 | NaN |
| PLK1 | PPP2R5E | 1.00E+00 | 1.00E+00 | 1.00E+00 | 1.00E+00 | 1.00E+00 | 2.88E-02 | 1.00E+00 | 1.00E+00 | 1.00E+00 | 1.00E+00 | NaN |
| PLK1 | STRN | 2.26E-02 | 1.00E+00 | 5.63E-02 | 1.00E+00 | 6.03E-01 | 1.00E+00 | 1.00E+00 | 4.60E-02 | 1.00E+00 | 1.00E+00 | NaN |
| PLK1 | STRN3 | 1.00E+00 | 1.00E+00 | 1.00E+00 | 1.00E+00 | 1.00E+00 | 1.00E+00 | 4.49E-01 | 1.00E+00 | 1.00E+00 | 1.00E+00 | NaN |
| PLK4 | PPP2CA | 1.00E+00 | 1.00E+00 | 1.00E+00 | 1.00E+00 | 1.00E+00 | 1.00E+00 | 1.00E+00 | 1.00E+00 | NaN | 3.9770408e-02 1.0 | 0.00E+00 |
| PLK4 | PPP2CB | 1.00E+00 | 1.00E+00 | 1.00E+00 | 1.00E+00 | 1.00E+00 | 1.00E+00 | 1.00E+00 | 1.00E+00 | NaN | 2.7884749e-02 1.0 | 0.00E+00 |
| PLK4 | PPP2R1A | 1.00E+00 | 1.00E+00 | 1.00E+00 | 1.00E+00 | 1.00E+00 | 7.63E-01 | 1.00E+00 | 1.00E+00 | NaN | 1.0000000e+00 1.0 | 0.00E+00 |
| PLK4 | PPP2R1B | 1.00E+00 | 1.00E+00 | 1.89E-01 | 1.00E+00 | 1.00E+00 | 1.00E+00 | 1.00E+00 | 1.00E+00 | NaN | 1.0000000e+00 1.0 | 0.00E+00 |
| PLK4 | PPP2R2A | 1.00E+00 | 1.00E+00 | 1.00E+00 | 1.00E+00 | 1.00E+00 | 1.00E+00 | 3.78E-02 | 1.00E+00 | NaN | 4.9001387e-02 1.0 | 0.00E+00 |
| PLK4 | PPP2R2B | 1.00E+00 | 1.00E+00 | 1.00E+00 | 1.00E+00 | 1.00E+00 | 8.07E-01 | 1.00E+00 | 1.00E+00 | NaN | 3.2703810e-02 1.0 | 0.00E+00 |
| PLK4 | PPP2R2C | 1.00E+00 | 1.00E+00 | 1.00E+00 | 5.98E-01 | 1.00E+00 | 1.00E+00 | 1.00E+00 | 1.00E+00 | NaN | 1.0000000e+00 6.5 | 3.22E+04 |
| PLK4 | PPP2R2D | 1.00E+00 | 1.00E+00 | 1.00E+00 | 1.00E+00 | 1.00E+00 | 1.00E+00 | 1.00E+00 | 1.00E+00 | NaN | 1.0000000e+00 4.7 | 2.57E+04 |
| PLK4 | PPP2R3A | 1.64E-01 | 1.00E+00 | 1.00E+00 | 1.00E+00 | 1.00E+00 | 7.66E-01 | 3.92E-02 | 1.00E+00 | NaN | 1.0000000e+00 1.0 | 0.00E+00 |
| PLK4 | PPP2R3B | 8.56E-02 | 5.85E-01 | 1.00E+00 | 5.93E-01 | 1.00E+00 | 7.90E-01 | 2.98E-02 | 1.00E+00 | NaN | 3.6969309e-02 2.9 | 3.86E+04 |
| PLK4 | PPP2R3C | 5.67E-02 | 7.96E-02 | 7.35E-01 | 1.00E+00 | 1.00E+00 | 2.68E-01 | 3.70E-02 | 1.00E+00 | NaN | 3.9456077e-02 6.5 | 6.11E+04 |
| PLK4 | PPP2R4 | 1.00E+00 | 1.00E+00 | 1.00E+00 | 1.00E+00 | 1.00E+00 | 1.00E+00 | 1.00E+00 | 1.00E+00 | NaN | 1.0000000e+00 1.0 | 0.00E+00 |
| PLK4 | PPP2R5A | 1.00E+00 | 1.00E+00 | 1.00E+00 | 1.00E+00 | 1.00E+00 | 1.00E+00 | 1.00E+00 | 1.00E+00 | NaN | 1.0000000e+00 1.0 | 0.00E+00 |
| PLK4 | PPP2R5B | 1.00E+00 | 1.00E+00 | 7.56E-01 | 1.00E+00 | 1.00E+00 | 1.00E+00 | 1.00E+00 | 1.00E+00 | NaN | 1.0000000e+00 6.1 | 5.10E+04 |
| PLK4 | PPP2R5C | 5.11E-01 | 5.18E-01 | 1.00E+00 | 1.00E+00 | 1.00E+00 | 1.00E+00 | 1.00E+00 | 1.00E+00 | NaN | 3.4021962e-02 1.0 | 0.00E+00 |
| PLK4 | PPP2R5D | 1.27E-01 | 4.42E-01 | 7.91E-01 | 5.66E-01 | 1.00E+00 | 1.00E+00 | 2.91E-02 | 1.00E+00 | NaN | 1.0000000e+00 1.0 | 0.00E+00 |
| PLK4 | PPP2R5E | 4.84E-01 | 7.44E-01 | 1.00E+00 | 1.00E+00 | 1.00E+00 | 1.00E+00 | 1.00E+00 | 1.00E+00 | NaN | 2.6760749e-02 3.9 | 7.59E+04 |
| PLK4 | STRN | 3.66E-01 | 1.00E+00 | 7.18E-01 | 1.00E+00 | 1.00E+00 | 1.00E+00 | 1.00E+00 | 1.00E+00 | NaN | 2.6995971e-02 2.6 | 4.65E+04 |
| PLK4 | STRN3 | 5.48E-02 | 3.51E-01 | 1.00E+00 | 1.00E+00 | 1.00E+00 | 1.00E+00 | 1.00E+00 | 1.00E+00 | NaN | 3.5328723e-02 4.8 | 2.72E+04 |
| AURKB | PPP2CA | 1.00E+00 | 1.00E+00 | 1.00E+00 | 3.50E-01 | 1.00E+00 | NaN | 1.00E+00 | 1.00E+00 | 1.00E+00 | 1.0000000e+00 1.0 | 0.00E+00 |
| AURKB | PPP2CB | 1.00E+00 | 1.00E+00 | 1.00E+00 | 1.00E+00 | 1.00E+00 | NaN | 1.00E+00 | 1.00E+00 | 1.00E+00 | 1.0000000e+00 5.4 | 3.94E+04 |
| AURKB | PPP2R1A | 1.00E+00 | 2.33E-02 | 1.00E+00 | 1.00E+00 | 5.89E-01 | NaN | 1.00E+00 | 4.42E-03 | 2.78E-01 | 1.0000000e+00 1.0 | 0.00E+00 |
| AURKB | PPP2R1B | 1.00E+00 | 1.00E+00 | 1.00E+00 | 1.00E+00 | 1.00E+00 | NaN | 1.00E+00 | 1.00E+00 | 1.00E+00 | 1.0000000e+00 1.0 | 0.00E+00 |
| AURKB | PPP2R2A | 1.00E+00 | 1.00E+00 | 5.41E-01 | 8.16E-01 | 7.57E-01 | NaN | 1.00E+00 | 7.71E-03 | 1.00E+00 | 1.0000000e+00 2.6 | 8.81E+04 |
| AURKB | PPP2R2B | 1.00E+00 | 1.00E+00 | 1.00E+00 | 1.00E+00 | 1.00E+00 | NaN | 1.00E+00 | 1.00E+00 | 1.00E+00 | 1.0000000e+00 1.0 | 0.00E+00 |
| AURKB | PPP2R2C | 1.00E+00 | 1.00E+00 | 1.00E+00 | 3.93E-01 | 7.94E-01 | NaN | 1.00E+00 | 1.00E+00 | 6.40E-01 | 1.0000000e+00 1.0 | 0.00E+00 |
| AURKB | PPP2R2D | 1.00E+00 | 1.00E+00 | 1.00E+00 | 1.00E+00 | 6.01E-01 | NaN | 1.00E+00 | 8.59E-03 | 1.00E+00 | 2.2848434e-03 1.0 | 0.00E+00 |
| AURKB | PPP2R3A | 8.25E-03 | 4.64E-02 | 3.48E-01 | 1.00E+00 | 1.00E+00 | NaN | 1.00E+00 | 5.96E-03 | 1.00E+00 | 1.0000000e+00 1.0 | 0.00E+00 |
| AURKB | PPP2R3B | 3.05E-05 | 1.55E-02 | 1.00E+00 | 4.55E-01 | 6.73E-01 | NaN | 1.00E+00 | 5.59E-03 | 1.00E+00 | 5.5295933e-06 1.0 | 0.00E+00 |
| AURKB | PPP2R3C | 3.86E-03 | 1.00E+00 | 1.00E+00 | 1.00E+00 | 6.70E-01 | NaN | 1.00E+00 | 8.18E-03 | 9.16E-01 | 1.0000000e+00 4.7 | 4.37E+04 |
| AURKB | PPP2R4 | 1.00E+00 | 1.00E+00 | 6.13E-01 | 3.82E-01 | 7.24E-01 | NaN | 1.00E+00 | 1.00E+00 | 6.90E-01 | 1.1913354e-03 1.0 | 0.00E+00 |
| AURKB | PPP2R5A | 1.00E+00 | 1.00E+00 | 2.07E-01 | 6.51E-01 | 1.00E+00 | NaN | 1.33E-02 | 1.00E+00 | 1.00E+00 | 1.0000000e+00 3.0 | 7.36E+04 |
| AURKB | PPP2R5B | 1.00E+00 | 1.00E+00 | 1.00E+00 | 1.00E+00 | 1.00E+00 | NaN | 1.00E+00 | 1.00E+00 | 7.94E-01 | 1.0000000e+00 1.0 | 0.00E+00 |
| AURKB | PPP2R5C | 2.11E-02 | 1.00E+00 | 1.00E+00 | 3.62E-01 | 1.00E+00 | NaN | 1.05E-02 | 1.00E+00 | 7.36E-01 | 1.0000000e+00 2.9 | 7.22E+04 |
| AURKB | PPP2R5D | 1.85E-04 | 8.69E-04 | 1.00E+00 | 6.99E-01 | 5.73E-01 | NaN | 7.79E-04 | 1.00E+00 | 1.00E+00 | 1.0000000e+00 3.3 | 6.99E+04 |
| AURKB | PPP2R5E | 1.43E-02 | 1.00E+00 | 5.28E-01 | 6.13E-01 | 1.00E+00 | NaN | 9.60E-03 | 1.00E+00 | 7.66E-01 | 1.0000000e+00 4.1 | 1.94E+04 |
| AURKB | STRN | 1.00E+00 | 1.00E+00 | 2.82E-01 | 1.00E+00 | 1.00E+00 | NaN | 9.50E-03 | 1.00E+00 | 1.00E+00 | 1.0000000e+00 2.8 | 2.40E+04 |
| AURKB | STRN3 | 1.00E+00 | 1.00E+00 | 5.80E-01 | 7.56E-01 | 7.75E-01 | NaN | 1.00E+00 | 1.00E+00 | 1.00E+00 | 1.0000000e+00 4.4 | 9.40E+04 |
| AURKA | PPP2CA | 1.00E+00 | 1.00E+00 | 1.00E+00 | 1.00E+00 | 1.00E+00 | 1.00E+00 | 1.00E+00 | 1.00E+00 | 1.00E+00 | 1.58E-01 | NaN |
| AURKA | PPP2CB | 1.00E+00 | 1.00E+00 | 1.00E+00 | 1.00E+00 | 1.00E+00 | 1.00E+00 | 1.00E+00 | 1.00E+00 | 1.00E+00 | 1.00E+00 | NaN |
| AURKA | PPP2R1A | 8.25E-04 | 1.00E+00 | 5.98E-03 | 1.00E+00 | 5.96E-03 | 1.00E+00 | 1.00E+00 | 1.00E+00 | 2.21E-01 | 1.00E+00 | NaN |
| AURKA | PPP2R1B | 1.00E+00 | 1.00E+00 | 1.00E+00 | 1.00E+00 | 1.00E+00 | 2.08E-03 | 1.00E+00 | 1.00E+00 | 1.00E+00 | 1.00E+00 | NaN |
| AURKA | PPP2R2A | 1.00E+00 | 1.00E+00 | 1.00E+00 | 1.00E+00 | 1.00E+00 | 1.00E+00 | 1.00E+00 | 1.00E+00 | 1.00E+00 | 1.00E+00 | NaN |
| AURKA | PPP2R2B | 1.00E+00 | 1.00E+00 | 1.00E+00 | 1.00E+00 | 1.00E+00 | 1.00E+00 | 1.00E+00 | 1.00E+00 | 1.00E+00 | 1.00E+00 | NaN |
| AURKA | PPP2R2C | 1.00E+00 | 1.00E+00 | 1.00E+00 | 1.00E+00 | 8.25E-03 | 1.00E+00 | 1.20E-01 | 1.00E+00 | 1.00E+00 | 1.00E+00 | NaN |
| AURKA | PPP2R2D | 1.00E+00 | 1.00E+00 | 1.00E+00 | 1.00E+00 | 4.50E-03 | 1.00E+00 | 7.01E-02 | 1.00E+00 | 1.00E+00 | 1.00E+00 | NaN |
| AURKA | PPP2R3A | 1.00E+00 | 4.61E-03 | 1.00E+00 | 1.00E+00 | 1.00E+00 | 2.26E-03 | 8.58E-02 | 1.00E+00 | 1.00E+00 | 1.00E+00 | NaN |
| AURKA | PPP2R3B | 1.92E-09 | 1.50E-04 | 2.67E-03 | 1.00E+00 | 4.00E-03 | 9.29E-03 | 6.71E-02 | 1.00E+00 | 1.00E+00 | 2.52E-01 | NaN |
| AURKA | PPP2R3C | 4.26E-04 | 1.34E-04 | 5.26E-04 | 1.00E+00 | 1.00E+00 | 1.00E+00 | 1.00E+00 | 1.00E+00 | 4.76E-01 | 1.00E+00 | NaN |
| AURKA | PPP2R4 | 1.00E+00 | 1.00E+00 | 1.00E+00 | 1.00E+00 | 8.85E-03 | 1.00E+00 | 1.00E+00 | 1.00E+00 | 2.08E-01 | 1.00E+00 | NaN |
| AURKA | PPP2R5A | 1.00E+00 | 1.00E+00 | 1.00E+00 | 1.00E+00 | 1.00E+00 | 1.00E+00 | 1.43E-01 | 1.00E+00 | 6.40E-01 | 1.00E+00 | NaN |
| AURKA | PPP2R5B | 1.00E+00 | 1.00E+00 | 1.00E+00 | 1.00E+00 | 1.00E+00 | 1.00E+00 | 1.00E+00 | 1.00E+00 | 1.00E+00 | 3.61E-01 | NaN |
| AURKA | PPP2R5C | 1.00E+00 | 1.00E+00 | 1.00E+00 | 1.00E+00 | 1.00E+00 | 1.00E+00 | 1.00E+00 | 8.69E-02 | 2.26E-01 | 1.00E+00 | NaN |
| AURKA | PPP2R5D | 4.05E-14 | 2.58E-04 | 4.65E-04 | 2.42E-03 | 1.00E+00 | 1.00E+00 | 1.15E-01 | 7.26E-02 | 5.51E-01 | 1.00E+00 | NaN |
| AURKA | PPP2R5E | 1.00E+00 | 1.00E+00 | 1.00E+00 | 1.00E+00 | 1.00E+00 | 3.82E-03 | 1.00E+00 | 6.55E-02 | 2.75E-01 | 1.00E+00 | NaN |
| AURKA | STRN | 8.07E-10 | 1.00E+00 | 1.00E+00 | 7.17E-04 | 1.00E+00 | 3.85E-03 | 1.49E-01 | 6.70E-02 | 6.62E-01 | 1.00E+00 | NaN |
| AURKA | STRN3 | 1.00E+00 | 1.06E-03 | 5.29E-04 | 1.00E+00 | 1.00E+00 | 1.00E+00 | 1.00E+00 | 8.10E-02 | 5.09E-01 | 1.00E+00 | NaN |
| CCNB1 | PPP2CA | 2.55E-21 | 2.64E-07 | 2.27E-03 | 1.28E-01 | 2.75E-04 | 1.85E-02 | NaN | 1.93E-02 | NaN | 5.31E-04 | NaN |
| CCNB1 | PPP2CB | 1.00E+00 | 1.00E+00 | 5.14E-02 | 1.00E+00 | 1.00E+00 | 1.00E+00 | NaN | 1.00E+00 | NaN | 1.00E+00 | NaN |
| CCNB1 | PPP2R1A | 5.31E-01 | 1.00E+00 | 1.00E+00 | 1.00E+00 | 1.00E+00 | 1.00E+00 | NaN | 1.00E+00 | NaN | 1.00E+00 | NaN |
| CCNB1 | PPP2R1B | 1.00E+00 | 1.00E+00 | 1.00E+00 | 1.00E+00 | 1.00E+00 | 3.37E-02 | NaN | 1.00E+00 | NaN | 1.00E+00 | NaN |
| CCNB1 | PPP2R2A | 1.00E+00 | 1.00E+00 | 5.20E-02 | 1.00E+00 | 1.00E+00 | 1.00E+00 | NaN | 1.00E+00 | NaN | 1.00E+00 | NaN |
| CCNB1 | PPP2R2B | 1.00E+00 | 1.00E+00 | 5.62E-02 | 1.00E+00 | 1.00E+00 | 1.00E+00 | NaN | 3.49E-01 | NaN | 1.33E-01 | NaN |
| CCNB1 | PPP2R2C | 1.00E+00 | 1.00E+00 | 5.41E-02 | 1.00E+00 | 3.07E-02 | 1.00E+00 | NaN | 3.65E-01 | NaN | 1.00E+00 | NaN |
| CCNB1 | PPP2R2D | 1.00E+00 | 1.00E+00 | 5.42E-02 | 8.31E-01 | 1.00E+00 | 1.00E+00 | NaN | 1.00E+00 | NaN | 1.00E+00 | NaN |
| CCNB1 | PPP2R3A | 1.00E+00 | 1.00E+00 | 1.00E+00 | 7.18E-01 | 1.00E+00 | 1.00E+00 | NaN | 1.00E+00 | NaN | 1.00E+00 | NaN |
| CCNB1 | PPP2R3B | 4.55E-04 | 3.53E-01 | 5.34E-02 | 2.20E-01 | 1.09E-02 | 1.00E+00 | NaN | 4.37E-01 | NaN | 3.16E-01 | NaN |
| CCNB1 | PPP2R3C | 2.28E-01 | 1.00E+00 | 4.96E-02 | 1.00E+00 | 1.00E+00 | 1.00E+00 | NaN | 4.22E-01 | NaN | 1.00E+00 | NaN |
| CCNB1 | PPP2R4 | 1.00E+00 | 1.00E+00 | 5.63E-02 | 7.51E-01 | 1.00E+00 | 3.26E-02 | NaN | 1.00E+00 | NaN | 1.00E+00 | NaN |
| CCNB1 | PPP2R5A | 1.00E+00 | 3.93E-01 | 1.00E+00 | 7.56E-01 | 1.00E+00 | 1.00E+00 | NaN | 1.00E+00 | NaN | 1.00E+00 | NaN |
| CCNB1 | PPP2R5B | 1.00E+00 | 4.08E-01 | 4.86E-02 | 1.00E+00 | 1.00E+00 | 1.00E+00 | NaN | 1.00E+00 | NaN | 1.00E+00 | NaN |
| CCNB1 | PPP2R5C | 4.38E-01 | 1.49E-01 | 1.00E+00 | 1.00E+00 | 1.00E+00 | 1.00E+00 | NaN | 3.44E-01 | NaN | 1.00E+00 | NaN |
| CCNB1 | PPP2R5D | 1.02E-01 | 1.15E-01 | 1.00E+00 | 6.16E-01 | 2.77E-03 | 1.00E+00 | NaN | 2.50E-01 | NaN | 1.00E+00 | NaN |
| CCNB1 | PPP2R5E | 3.86E-02 | 4.48E-02 | 5.23E-02 | 1.00E+00 | 1.00E+00 | 1.00E+00 | NaN | 3.14E-01 | NaN | 1.00E+00 | NaN |
| CCNB1 | STRN | 1.00E+00 | 1.00E+00 | 1.00E+00 | 7.67E-01 | 1.00E+00 | 1.00E+00 | NaN | 1.00E+00 | NaN | 3.66E-01 | NaN |
| CCNB1 | STRN3 | 4.35E-01 | 1.09E-01 | 5.80E-02 | 1.00E+00 | 1.00E+00 | 1.00E+00 | NaN | 3.26E-01 | NaN | 1.00E+00 | NaN |
| CCNB2 | PPP2CA | 1.00E+00 | 1.00E+00 | 1.00E+00 | 1.00E+00 | 5.53E-01 | 1.00E+00 | 1.83E-02 | 1.00E+00 | 1.00E+00 | 1.00E+00 | NaN |
| CCNB2 | PPP2CB | 1.00E+00 | 1.00E+00 | 1.00E+00 | 1.00E+00 | 1.00E+00 | 1.00E+00 | 1.00E+00 | 1.00E+00 | 1.00E+00 | 1.00E+00 | NaN |
| CCNB2 | PPP2R1A | 1.00E+00 | 6.42E-02 | 1.74E-01 | 1.00E+00 | 1.00E+00 | 1.00E+00 | 1.00E+00 | 1.00E+00 | 4.41E-01 | 1.00E+00 | NaN |
| CCNB2 | PPP2R1B | 1.00E+00 | 7.05E-02 | 3.72E-01 | 1.00E+00 | 1.00E+00 | 1.00E+00 | 1.71E-02 | 1.31E-02 | 1.00E+00 | 1.00E+00 | NaN |
| CCNB2 | PPP2R2A | 1.00E+00 | 1.00E+00 | 1.00E+00 | 1.99E-01 | 1.00E+00 | 1.00E+00 | 1.00E+00 | 1.00E+00 | 1.00E+00 | 1.00E+00 | NaN |
| CCNB2 | PPP2R2B | 1.00E+00 | 1.00E+00 | 1.00E+00 | 1.00E+00 | 5.84E-01 | 1.00E+00 | 1.00E+00 | 1.00E+00 | 1.00E+00 | 1.00E+00 | NaN |
| CCNB2 | PPP2R2C | 1.00E+00 | 1.00E+00 | 1.00E+00 | 1.00E+00 | 5.96E-01 | 1.00E+00 | 1.92E-02 | 1.00E+00 | 1.00E+00 | 1.00E+00 | NaN |
| CCNB2 | PPP2R2D | 1.00E+00 | 1.00E+00 | 1.00E+00 | 2.02E-01 | 1.00E+00 | 1.00E+00 | 1.00E+00 | 1.00E+00 | 1.00E+00 | 1.00E+00 | NaN |
| CCNB2 | PPP2R3A | 2.17E-01 | 7.30E-02 | 1.00E+00 | 1.00E+00 | 6.18E-01 | 1.00E+00 | 1.00E+00 | 1.00E+00 | 1.00E+00 | 1.00E+00 | NaN |
| CCNB2 | PPP2R3B | 4.06E-01 | 7.42E-02 | 3.97E-01 | 2.71E-01 | 1.00E+00 | 1.00E+00 | 1.00E+00 | 8.14E-03 | 1.00E+00 | 1.00E+00 | NaN |
| CCNB2 | PPP2R3C | 1.00E+00 | 5.17E-02 | 1.00E+00 | 2.25E-01 | 1.00E+00 | 1.00E+00 | 1.00E+00 | 1.00E+00 | 2.97E-01 | 1.00E+00 | NaN |
| CCNB2 | PPP2R4 | 1.00E+00 | 1.00E+00 | 1.00E+00 | 1.00E+00 | 1.00E+00 | 1.00E+00 | 1.00E+00 | 1.43E-02 | 1.00E+00 | 1.00E+00 | NaN |
| CCNB2 | PPP2R5A | 1.00E+00 | 1.00E+00 | 1.00E+00 | 1.96E-01 | 1.00E+00 | 1.00E+00 | 1.00E+00 | 1.00E+00 | 1.00E+00 | 9.45E-03 | NaN |
| CCNB2 | PPP2R5B | 1.00E+00 | 6.52E-02 | 1.00E+00 | 1.00E+00 | 1.00E+00 | 1.00E+00 | 1.00E+00 | 1.00E+00 | 1.00E+00 | 1.00E+00 | NaN |
| CCNB2 | PPP2R5C | 1.00E+00 | 1.00E+00 | 1.00E+00 | 1.00E+00 | 1.00E+00 | 1.00E+00 | 1.00E+00 | 6.17E-03 | 2.85E-01 | 1.00E+00 | NaN |
| CCNB2 | PPP2R5D | 4.51E-01 | 5.56E-02 | 1.00E+00 | 1.00E+00 | 1.79E-01 | 1.00E+00 | 9.44E-03 | 1.00E+00 | 5.66E-01 | 1.00E+00 | NaN |
| CCNB2 | PPP2R5E | 1.00E+00 | 1.00E+00 | 1.00E+00 | 2.59E-01 | 1.00E+00 | 1.00E+00 | 1.00E+00 | 1.00E+00 | 3.31E-01 | 1.00E+00 | NaN |
| CCNB2 | STRN | 5.80E-02 | 1.00E+00 | 1.39E-01 | 2.10E-01 | 6.29E-01 | 1.00E+00 | 1.77E-02 | 1.26E-02 | 1.00E+00 | 1.00E+00 | NaN |
| CCNB2 | STRN3 | 1.00E+00 | 1.00E+00 | 1.00E+00 | 1.81E-01 | 1.00E+00 | 1.00E+00 | 1.00E+00 | 1.00E+00 | 1.00E+00 | 1.00E+00 | NaN |
| NDC80 | PPP2CA | 1.00E+00 | 1.00E+00 | 1.00E+00 | 7.37E-01 | 6.92E-01 | 1.00E+00 | 8.02E-01 | 1.00E+00 | 1.00E+00 | 1.00E+00 | NaN |
| NDC80 | PPP2CB | 1.00E+00 | 1.00E+00 | 1.00E+00 | 1.00E+00 | 1.00E+00 | 1.00E+00 | 1.00E+00 | 2.50E-02 | 1.00E+00 | 1.00E+00 | NaN |
| NDC80 | PPP2R1A | 1.00E+00 | 1.00E+00 | 1.00E+00 | 1.00E+00 | 1.00E+00 | 1.00E+00 | 6.89E-01 | 2.57E-02 | 6.80E-02 | 1.00E+00 | NaN |
| NDC80 | PPP2R1B | 1.00E+00 | 1.00E+00 | 1.00E+00 | 1.00E+00 | 1.00E+00 | 1.00E+00 | 1.00E+00 | 1.00E+00 | 6.27E-02 | 1.00E+00 | NaN |
| NDC80 | PPP2R2A | 1.00E+00 | 1.00E+00 | 1.00E+00 | 1.00E+00 | 1.00E+00 | 1.00E+00 | 1.00E+00 | 2.57E-02 | 1.00E+00 | 1.00E+00 | NaN |
| NDC80 | PPP2R2B | 1.00E+00 | 1.00E+00 | 1.00E+00 | 1.00E+00 | 4.70E-01 | 1.00E+00 | 1.00E+00 | 2.89E-02 | 1.00E+00 | 1.00E+00 | NaN |
| NDC80 | PPP2R2C | 1.00E+00 | 1.00E+00 | 1.00E+00 | 1.00E+00 | 1.00E+00 | 1.00E+00 | 1.00E+00 | 1.00E+00 | 6.49E-02 | 3.30E-02 | NaN |
| NDC80 | PPP2R2D | 1.00E+00 | 1.00E+00 | 1.00E+00 | 1.00E+00 | 1.00E+00 | 1.00E+00 | 1.00E+00 | 1.00E+00 | 1.00E+00 | 4.07E-02 | NaN |
| NDC80 | PPP2R3A | 1.04E-03 | 7.94E-02 | 1.35E-03 | 1.00E+00 | 1.00E+00 | 1.00E+00 | 1.00E+00 | 1.00E+00 | 1.00E+00 | 1.00E+00 | NaN |
| NDC80 | PPP2R3B | 1.04E-01 | 7.92E-02 | 1.00E+00 | 6.91E-01 | 1.00E+00 | 1.00E+00 | 1.00E+00 | 1.00E+00 | 1.00E+00 | 4.72E-02 | NaN |
| NDC80 | PPP2R3C | 1.00E+00 | 1.00E+00 | 1.00E+00 | 1.00E+00 | 1.00E+00 | 1.00E+00 | 1.00E+00 | 1.00E+00 | 1.00E+00 | 4.77E-02 | NaN |
| NDC80 | PPP2R4 | 1.00E+00 | 1.00E+00 | 1.23E-03 | 1.00E+00 | 1.00E+00 | 1.00E+00 | 8.72E-01 | 1.00E+00 | 1.00E+00 | 1.00E+00 | NaN |
| NDC80 | PPP2R5A | 1.00E+00 | 1.00E+00 | 1.00E+00 | 1.00E+00 | 1.00E+00 | 1.00E+00 | 8.52E-01 | 1.00E+00 | 1.00E+00 | 1.00E+00 | NaN |
| NDC80 | PPP2R5B | 1.00E+00 | 1.00E+00 | 1.00E+00 | 1.00E+00 | 1.00E+00 | 1.00E+00 | 1.00E+00 | 1.00E+00 | 1.00E+00 | 1.00E+00 | NaN |
| NDC80 | PPP2R5C | 6.05E-02 | 1.00E+00 | 1.00E+00 | 1.00E+00 | 1.00E+00 | 1.00E+00 | 1.00E+00 | 1.00E+00 | 1.00E+00 | 3.21E-02 | NaN |
| NDC80 | PPP2R5D | 8.60E-07 | 1.55E-05 | 1.13E-03 | 5.75E-01 | 7.66E-01 | 1.00E+00 | 9.06E-01 | 2.01E-02 | 1.00E+00 | 1.00E+00 | NaN |
| NDC80 | PPP2R5E | 1.07E-03 | 1.00E+00 | 1.10E-03 | 1.00E+00 | 1.00E+00 | 1.00E+00 | 8.61E-01 | 2.35E-02 | 1.00E+00 | 4.47E-02 | NaN |
| NDC80 | STRN | 8.09E-03 | 1.00E+00 | 8.49E-05 | 1.00E+00 | 9.18E-01 | 1.00E+00 | 1.00E+00 | 2.73E-02 | 1.00E+00 | 5.90E-02 | NaN |
| NDC80 | STRN3 | 5.55E-03 | 1.00E+00 | 2.13E-04 | 1.00E+00 | 1.00E+00 | 1.00E+00 | 1.00E+00 | 2.28E-02 | 1.00E+00 | 3.77E-02 | NaN |
| PTTG1 | PPP2CA | 7.77E-15 | 1.00E+00 | 3.00E-01 | 2.86E-03 | 6.11E-05 | 1.03E-01 | 7.77E-03 | 1.69E-03 | 3.27E-01 | 3.58E-10 | NaN |
| PTTG1 | PPP2CB | 1.00E+00 | 1.00E+00 | 1.00E+00 | 1.00E+00 | 1.00E+00 | 1.00E+00 | 1.00E+00 | 1.00E+00 | 1.00E+00 | 1.00E+00 | NaN |
| PTTG1 | PPP2R1A | 4.99E-01 | 1.59E-04 | 1.00E+00 | 1.91E-01 | 1.00E+00 | 1.00E+00 | 1.00E+00 | 1.00E+00 | 1.00E+00 | 1.00E+00 | NaN |
| PTTG1 | PPP2R1B | 1.00E+00 | 1.00E+00 | 1.00E+00 | 1.00E+00 | 1.00E+00 | 1.00E+00 | 1.34E-02 | 1.00E+00 | 5.54E-01 | 1.00E+00 | NaN |
| PTTG1 | PPP2R2A | 1.00E+00 | 1.00E+00 | 1.00E+00 | 1.00E+00 | 1.00E+00 | 1.00E+00 | 1.00E+00 | 1.00E+00 | 1.00E+00 | 1.00E+00 | NaN |
| PTTG1 | PPP2R2B | 1.02E-01 | 1.00E+00 | 1.56E-01 | 1.00E+00 | 1.00E+00 | 1.00E+00 | 8.36E-03 | 9.93E-01 | 1.00E+00 | 4.71E-04 | NaN |
| PTTG1 | PPP2R2C | 1.00E+00 | 1.00E+00 | 1.00E+00 | 2.87E-01 | 1.00E+00 | 1.00E+00 | 1.00E+00 | 9.04E-01 | 1.00E+00 | 1.00E+00 | NaN |
| PTTG1 | PPP2R2D | 1.00E+00 | 1.00E+00 | 8.07E-01 | 1.00E+00 | 2.50E-02 | 1.00E+00 | 1.00E+00 | 1.00E+00 | 1.00E+00 | 1.00E+00 | NaN |
| PTTG1 | PPP2R3A | 4.26E-01 | 1.00E+00 | 1.00E+00 | 1.00E+00 | 2.52E-02 | 1.00E+00 | 1.00E+00 | 1.00E+00 | 1.00E+00 | 1.00E+00 | NaN |
| PTTG1 | PPP2R3B | 7.12E-03 | 7.61E-04 | 7.24E-01 | 1.28E-01 | 1.77E-02 | 9.30E-02 | 1.00E+00 | 1.00E+00 | 1.00E+00 | 1.00E+00 | NaN |
| PTTG1 | PPP2R3C | 1.00E+00 | 1.00E+00 | 8.64E-01 | 1.00E+00 | 1.00E+00 | 1.00E+00 | 1.00E+00 | 1.00E+00 | 1.00E+00 | 1.00E+00 | NaN |
| PTTG1 | PPP2R4 | 3.14E-01 | 1.11E-03 | 1.58E-01 | 3.15E-01 | 1.00E+00 | 2.39E-01 | 1.00E+00 | 1.00E+00 | 1.00E+00 | 1.00E+00 | NaN |
| PTTG1 | PPP2R5A | 1.00E+00 | 1.00E+00 | 8.49E-01 | 1.60E-01 | 1.00E+00 | 1.00E+00 | 1.00E+00 | 1.00E+00 | 3.61E-01 | 1.00E+00 | NaN |
| PTTG1 | PPP2R5B | 1.00E+00 | 1.00E+00 | 6.86E-01 | 1.00E+00 | 1.00E+00 | 1.00E+00 | 1.00E+00 | 1.00E+00 | 1.00E+00 | 1.00E+00 | NaN |
| PTTG1 | PPP2R5C | 1.00E+00 | 1.00E+00 | 1.00E+00 | 1.00E+00 | 1.00E+00 | 1.00E+00 | 1.00E+00 | 1.00E+00 | 1.00E+00 | 1.00E+00 | NaN |
| PTTG1 | PPP2R5D | 1.88E-01 | 3.06E-04 | 1.00E+00 | 1.00E+00 | 4.65E-03 | 1.00E+00 | 5.35E-03 | 9.46E-01 | 5.47E-01 | 1.00E+00 | NaN |
| PTTG1 | PPP2R5E | 1.00E+00 | 1.00E+00 | 1.00E+00 | 1.00E+00 | 1.00E+00 | 1.00E+00 | 1.00E+00 | 1.00E+00 | 1.00E+00 | 1.00E+00 | NaN |
| PTTG1 | STRN | 1.00E+00 | 1.00E+00 | 1.00E+00 | 1.00E+00 | 1.00E+00 | 2.38E-01 | 1.00E+00 | 1.00E+00 | 4.22E-01 | 1.00E+00 | NaN |
| PTTG1 | STRN3 | 1.00E+00 | 1.00E+00 | 7.84E-01 | 1.00E+00 | 1.00E+00 | 1.00E+00 | 1.00E+00 | 1.00E+00 | 1.00E+00 | 1.00E+00 | NaN |
| FZR1 | PPP2CA | 1.00E+00 | 1.00E+00 | 1.00E+00 | 1.00E+00 | 9.63E-02 | NaN | 1.00E+00 | 1.00E+00 | 1.00E+00 | 9.69E-02 | NaN |
| FZR1 | PPP2CB | 2.83E-03 | 1.00E+00 | 1.00E+00 | 1.00E+00 | 1.00E+00 | NaN | 4.65E-01 | 4.04E-01 | 1.00E+00 | 1.00E+00 | NaN |
| FZR1 | PPP2R1A | 1.26E-06 | 1.81E-03 | 2.89E-04 | 4.15E-02 | 1.40E-02 | NaN | 1.00E+00 | 5.32E-01 | 2.38E-02 | 1.32E-01 | NaN |
| FZR1 | PPP2R1B | 1.00E+00 | 1.00E+00 | 1.00E+00 | 1.00E+00 | 1.00E+00 | NaN | 5.42E-01 | 1.00E+00 | 1.00E+00 | 2.06E-01 | NaN |
| FZR1 | PPP2R2A | 1.00E+00 | 1.00E+00 | 1.00E+00 | 1.00E+00 | 1.00E+00 | NaN | 5.38E-01 | 4.70E-01 | 1.00E+00 | 1.87E-01 | NaN |
| FZR1 | PPP2R2B | 1.00E+00 | 1.00E+00 | 1.00E+00 | 1.00E+00 | 1.00E+00 | NaN | 4.63E-01 | 1.00E+00 | 2.50E-03 | 1.00E+00 | NaN |
| FZR1 | PPP2R2C | 1.00E+00 | 1.00E+00 | 1.00E+00 | 1.00E+00 | 9.69E-02 | NaN | 1.00E+00 | 1.00E+00 | 5.77E-02 | 1.00E+00 | NaN |
| FZR1 | PPP2R2D | 1.00E+00 | 1.00E+00 | 1.00E+00 | 1.00E+00 | 9.35E-02 | NaN | 1.00E+00 | 4.20E-01 | 1.00E+00 | 1.00E+00 | NaN |
| FZR1 | PPP2R3A | 1.00E+00 | 1.00E+00 | 6.06E-04 | 5.63E-01 | 1.00E+00 | NaN | 1.00E+00 | 4.84E-01 | 1.00E+00 | 1.00E+00 | NaN |
| FZR1 | PPP2R3B | 3.42E-03 | 8.34E-04 | 1.00E+00 | 5.50E-01 | 9.47E-02 | NaN | 1.00E+00 | 2.49E-01 | 8.97E-03 | 1.00E+00 | NaN |
| FZR1 | PPP2R3C | 9.09E-02 | 1.00E+00 | 1.00E+00 | 1.00E+00 | 1.00E+00 | NaN | 1.00E+00 | 1.00E+00 | 6.81E-02 | 1.00E+00 | NaN |
| FZR1 | PPP2R4 | 5.04E-02 | 1.00E+00 | 1.56E-03 | 1.00E+00 | 1.00E+00 | NaN | 1.35E-01 | 1.00E+00 | 4.89E-03 | 1.00E+00 | NaN |
| FZR1 | PPP2R5A | 1.00E+00 | 1.00E+00 | 1.00E+00 | 3.67E-01 | 3.47E-02 | NaN | 1.00E+00 | 4.14E-01 | 1.00E+00 | 1.00E+00 | NaN |
| FZR1 | PPP2R5B | 1.00E+00 | 3.03E-03 | 1.00E+00 | 1.00E+00 | 1.00E+00 | NaN | 5.11E-01 | 1.00E+00 | 1.00E+00 | 1.00E+00 | NaN |
| FZR1 | PPP2R5C | 2.53E-01 | 1.00E+00 | 1.00E+00 | 1.00E+00 | 1.00E+00 | NaN | 1.00E+00 | 1.00E+00 | 3.15E-02 | 1.00E+00 | NaN |
| FZR1 | PPP2R5D | 1.00E+00 | 1.00E+00 | 1.00E+00 | 6.68E-01 | 5.82E-02 | NaN | 1.00E+00 | 1.00E+00 | 1.00E+00 | 1.00E+00 | NaN |
| FZR1 | PPP2R5E | 1.00E+00 | 1.00E+00 | 1.00E+00 | 1.00E+00 | 1.00E+00 | NaN | 1.00E+00 | 1.00E+00 | 1.00E+00 | 1.00E+00 | NaN |
| FZR1 | STRN | 1.00E+00 | 1.00E+00 | 4.12E-05 | 1.00E+00 | 1.00E+00 | NaN | 1.00E+00 | 1.00E+00 | 1.00E+00 | 1.00E+00 | NaN |
| FZR1 | STRN3 | 1.00E+00 | 1.00E+00 | 1.00E+00 | 1.00E+00 | 1.00E+00 | NaN | 1.00E+00 | 1.00E+00 | 1.00E+00 | 1.00E+00 | NaN |

Supplementary Table 4

| cancer types | | Breast cancer | Lung cancer | Colon cancer | Linver cancer | Prostate cancer | Ovarian cancer | Cervical cancer | Pancreatic cancer | Kidney cancer | AML |
| --- | --- | --- | --- | --- | --- | --- | --- | --- | --- | --- | --- |
| genes | | clinical | clinical | clinical | clinical | clinical | clinical | clinical | clinical | clinical | clinical |
| SAC genes | PP2A genes | logrank test | logrank test | logrank test | logrank test | logrank test | logrank test | logrank test | logrank test | logrank test | logrank test |
| AURKA | PPP2R5D | 6.13E-01 | 5.43E-01 | 7.12E-01 | 7.59E-01 | 7.74E-01 | 6.62E-01 | 7.87E-01 | 6.55E-01 | 4.51E-02 | 1.29E-01 |
| AURKA | PPP2R5B | 1.00E+00 | 4.89E-01 | 5.52E-01 | 3.98E-02 | 3.88E-01 | 8.54E-01 | 2.04E-01 | 9.25E-01 | 4.50E-02 | 2.16E-02 |
| AURKA | PPP2R3B | 5.23E-01 | 8.57E-01 | 1.35E-01 | 4.97E-01 | 1.06E-01 | 3.91E-01 | 8.96E-01 | 9.83E-01 | 6.16E-03 | 6.49E-02 |
| AURKA | PPP2R2D | 6.42E-01 | 5.18E-01 | 1.84E-02 | 1.00E+00 | 5.88E-01 | 2.75E-01 | 7.54E-01 | 5.51E-01 | 6.89E-01 | 9.16E-01 |
| AURKA | PPP2R1A | 5.72E-01 | 2.15E-01 | 1.17E-01 | 4.63E-02 | 2.35E-01 | 1.00E+00 | 1.16E-01 | 3.67E-01 | 9.02E-01 | 1.00E+00 |
| AURKA | PPP2CB | 1.00E+00 | 2.50E-01 | 8.88E-01 | 1.00E+00 | 8.96E-01 | 7.28E-01 | 1.44E-01 | 1.00E+00 | 9.59E-01 | 6.28E-01 |
| AURKB | PPP2R5D | 2.31E-01 | 3.18E-01 | 9.53E-01 | 2.70E-01 | 4.59E-01 | 5.02E-01 | 1.24E-01 | 6.53E-01 | 3.91E-01 | 5.08E-01 |
| AURKB | PPP2R5B | 1.00E+00 | 5.07E-01 | 9.08E-02 | 3.60E-02 | 6.94E-01 | 7.95E-01 | 3.10E-02 | 8.52E-01 | 8.62E-02 | 1.00E+00 |
| AURKB | PPP2R3B | 5.89E-01 | 9.18E-01 | 2.10E-01 | 1.11E-01 | 1.06E-01 | 9.84E-01 | 7.82E-01 | 1.00E+00 | 3.05E-02 | 1.02E-01 |
| AURKB | PPP2R2D | 8.72E-01 | 5.56E-01 | 1.11E-02 | 2.46E-01 | 4.64E-01 | 6.98E-01 | 3.91E-01 | 5.66E-01 | 7.35E-01 | 9.91E-01 |
| AURKB | PPP2R1A | 5.18E-01 | 9.45E-02 | 3.35E-01 | 1.29E-02 | 3.69E-01 | 1.00E+00 | 2.69E-01 | 2.18E-01 | 8.02E-01 | 1.00E+00 |
| AURKB | PPP2CB | 1.00E+00 | 4.60E-01 | 1.81E-01 | 1.00E+00 | 3.34E-01 | 8.39E-01 | 6.26E-01 | 1.00E+00 | 9.07E-01 | 6.26E-01 |
| BUB1 | PPP2R5D | 6.57E-01 | 5.24E-01 | 8.58E-01 | 3.79E-01 | 3.87E-01 | 7.51E-01 | 9.68E-01 | 8.60E-01 | 3.25E-01 | 3.15E-01 |
| BUB1 | PPP2R5B | 7.47E-02 | 2.95E-01 | 2.06E-01 | 3.52E-02 | 2.74E-01 | 9.02E-01 | 2.13E-01 | 9.10E-01 | 1.58E-02 | 1.96E-02 |
| BUB1 | PPP2R3B | 1.07E-01 | 8.35E-01 | 7.41E-01 | 3.63E-01 | 1.20E-01 | 2.13E-01 | 8.19E-01 | 9.69E-01 | 1.88E-03 | 9.82E-02 |
| BUB1 | PPP2R2D | 8.29E-01 | 5.23E-01 | 9.01E-02 | 3.62E-01 | 2.38E-01 | 1.07E-01 | 8.08E-01 | 7.76E-01 | 3.39E-01 | 9.66E-01 |
| BUB1 | PPP2R1A | 2.86E-01 | 2.77E-01 | 7.16E-02 | 3.98E-02 | 1.85E-01 | 1.00E+00 | 3.89E-02 | 2.07E-01 | 2.71E-01 | 1.00E+00 |
| BUB1 | PPP2CB | 1.00E+00 | 2.70E-01 | 1.11E-01 | 1.00E+00 | 9.06E-01 | 9.70E-01 | 4.75E-01 | 3.26E-01 | 9.66E-01 | 5.78E-01 |
| BUB1B | PPP2R5D | 5.03E-01 | 5.92E-01 | 8.15E-01 | 3.66E-01 | 7.29E-01 | 5.70E-01 | 5.96E-01 | 4.74E-01 | 5.59E-01 | 2.46E-01 |
| BUB1B | PPP2R5B | 8.50E-01 | 3.76E-01 | 1.12E-01 | 1.83E-01 | 3.14E-01 | 7.43E-01 | 1.61E-01 | 9.10E-01 | 2.87E-03 | 2.18E-02 |
| BUB1B | PPP2R3B | 1.63E-01 | 9.14E-01 | 2.32E-01 | 1.53E-01 | 9.74E-02 | 4.24E-01 | 1.86E-01 | 9.97E-01 | 1.84E-04 | 1.06E-01 |
| BUB1B | PPP2R2D | 9.18E-01 | 6.31E-01 | 3.95E-02 | 4.68E-01 | 5.82E-01 | 5.72E-01 | 6.45E-01 | 7.39E-01 | 7.45E-02 | 9.66E-01 |
| BUB1B | PPP2R1A | 5.69E-01 | 1.56E-01 | 2.74E-01 | 6.52E-03 | 1.92E-01 | 1.00E+00 | 9.11E-02 | 3.65E-01 | 2.29E-01 | 1.00E+00 |
| BUB1B | PPP2CB | 1.00E+00 | 3.68E-01 | 1.00E+00 | 1.00E+00 | 9.11E-01 | 9.90E-01 | 5.88E-01 | 3.69E-01 | 9.83E-01 | 4.85E-01 |
| BUB3 | PPP2R5D | 7.67E-02 | 5.47E-01 | 8.01E-01 | 4.15E-01 | 6.67E-01 | 9.20E-01 | 6.98E-01 | 3.57E-01 | 5.16E-01 | 4.36E-01 |
| BUB3 | PPP2R5B | 3.23E-01 | 4.69E-01 | 1.73E-01 | 1.82E-01 | 4.81E-01 | 8.29E-01 | 3.41E-02 | 9.94E-01 | 1.00E+00 | 1.00E+00 |
| BUB3 | PPP2R3B | 6.62E-02 | 2.25E-01 | 6.27E-02 | 5.63E-02 | 4.19E-02 | 8.33E-01 | 7.14E-01 | 9.97E-01 | 1.49E-02 | 3.09E-01 |
| BUB3 | PPP2R2D | 5.65E-01 | 5.99E-01 | 9.25E-02 | 3.15E-01 | 5.12E-01 | 7.03E-01 | 3.79E-01 | 7.77E-01 | 3.99E-01 | 9.96E-01 |
| BUB3 | PPP2R1A | 5.79E-01 | 2.20E-01 | 2.31E-01 | 1.73E-02 | 1.35E-01 | 1.00E+00 | 1.76E-01 | 4.06E-01 | 7.05E-01 | 1.00E+00 |
| BUB3 | PPP2CB | 5.46E-01 | 2.21E-01 | 1.94E-01 | 1.00E+00 | 4.74E-01 | 9.35E-01 | 4.04E-01 | 1.00E+00 | 9.53E-01 | 8.90E-01 |
| CCNB1 | PPP2R5D | 5.21E-01 | 3.92E-01 | 7.64E-01 | 4.57E-01 | 7.30E-01 | 8.09E-01 | 7.39E-01 | 5.81E-01 | 1.71E-01 | 1.70E-01 |
| CCNB1 | PPP2R5B | 1.00E+00 | 5.53E-01 | 1.05E-01 | 1.65E-02 | 7.46E-01 | 9.21E-01 | 8.48E-02 | 9.64E-01 | 1.66E-02 | 1.72E-02 |
| CCNB1 | PPP2R3B | 4.01E-01 | 8.55E-01 | 8.39E-02 | 3.44E-01 | 1.00E-01 | 8.82E-01 | 7.02E-01 | 1.00E+00 | 1.31E-02 | 2.34E-02 |
| CCNB1 | PPP2R2D | 8.08E-01 | 6.07E-01 | 9.12E-02 | 2.94E-01 | 4.05E-01 | 7.49E-01 | 4.23E-01 | 5.66E-01 | 2.91E-01 | 9.28E-01 |
| CCNB1 | PPP2R1A | 5.65E-01 | 2.70E-01 | 3.98E-01 | 2.40E-02 | 3.97E-02 | 1.00E+00 | 6.76E-01 | 2.80E-01 | 4.84E-01 | 1.00E+00 |
| CCNB1 | PPP2CB | 1.00E+00 | 4.61E-01 | 2.49E-01 | 1.00E+00 | 4.39E-01 | 8.54E-01 | 7.13E-01 | 3.47E-01 | 9.76E-01 | 5.41E-01 |
| CCNB2 | PPP2R5D | 5.27E-01 | 6.69E-01 | 7.54E-01 | 6.20E-01 | 5.58E-01 | 6.43E-01 | 1.35E-01 | 7.34E-01 | 4.37E-01 | 8.09E-02 |
| CCNB2 | PPP2R5B | 1.32E-01 | 6.83E-01 | 1.02E-01 | 3.01E-02 | 3.22E-01 | 8.14E-01 | 1.95E-01 | 8.86E-01 | 1.14E-02 | 8.41E-03 |
| CCNB2 | PPP2R3B | 6.98E-01 | 9.34E-01 | 1.75E-01 | 9.88E-02 | 6.68E-02 | 8.60E-01 | 6.45E-01 | 1.00E+00 | 1.90E-03 | 2.37E-02 |
| CCNB2 | PPP2R2D | 8.61E-01 | 8.42E-01 | 9.08E-02 | 2.20E-01 | 5.20E-01 | 5.25E-01 | 7.42E-01 | 5.17E-01 | 1.71E-01 | 9.55E-01 |
| CCNB2 | PPP2R1A | 5.07E-01 | 5.41E-02 | 2.74E-01 | 6.83E-02 | 7.38E-02 | 1.00E+00 | 1.16E-01 | 3.22E-01 | 2.20E-01 | 1.00E+00 |
| CCNB2 | PPP2CB | 1.00E+00 | 3.85E-01 | 2.96E-01 | 1.40E-01 | 2.33E-01 | 9.81E-01 | 5.09E-01 | 2.59E-01 | 9.81E-01 | 6.38E-01 |
| CDC20 | PPP2R5D | 6.88E-01 | 4.04E-01 | 7.51E-01 | 4.24E-01 | 4.10E-01 | 6.67E-01 | 7.03E-01 | 7.21E-01 | 4.96E-01 | 3.20E-01 |
| CDC20 | PPP2R5B | 1.00E+00 | 6.06E-01 | 4.85E-02 | 2.79E-02 | 6.60E-01 | 6.51E-01 | 6.32E-02 | 8.89E-01 | 3.96E-02 | 1.93E-02 |
| CDC20 | PPP2R3B | 5.53E-01 | 9.46E-01 | 3.02E-01 | 6.64E-01 | 8.59E-02 | 8.36E-01 | 7.40E-01 | 1.00E+00 | 2.06E-02 | 1.21E-01 |
| CDC20 | PPP2R2D | 8.84E-01 | 2.75E-01 | 6.71E-02 | 3.14E-01 | 4.32E-01 | 2.09E-01 | 5.50E-01 | 6.32E-01 | 5.64E-01 | 9.87E-01 |
| CDC20 | PPP2R1A | 5.31E-01 | 1.51E-01 | 2.50E-01 | 1.47E-01 | 3.09E-01 | 1.00E+00 | 3.48E-01 | 7.41E-01 | 8.07E-01 | 1.00E+00 |
| CDC20 | PPP2CB | 1.00E+00 | 4.08E-01 | 2.27E-01 | 1.00E+00 | 3.42E-01 | 9.00E-01 | 2.59E-01 | 1.00E+00 | 9.55E-01 | 5.51E-01 |
| CENPE | PPP2R5D | 5.13E-01 | 4.31E-01 | 7.54E-01 | 4.68E-01 | 5.96E-01 | 7.69E-01 | 6.92E-01 | 7.45E-01 | 4.74E-01 | 7.51E-02 |
| CENPE | PPP2R5B | 7.58E-02 | 3.91E-01 | 1.80E-01 | 7.97E-02 | 4.87E-01 | 7.93E-01 | 1.63E-01 | 8.81E-01 | 5.80E-03 | 3.34E-02 |
| CENPE | PPP2R3B | 1.33E-01 | 7.68E-01 | 5.55E-01 | 3.33E-01 | 7.75E-02 | 6.61E-01 | 6.12E-01 | 9.82E-01 | 3.94E-05 | 3.55E-02 |
| CENPE | PPP2R2D | 7.23E-01 | 3.32E-01 | 5.88E-02 | 3.90E-01 | 7.77E-01 | 2.36E-01 | 3.88E-01 | 3.38E-01 | 8.60E-02 | 9.32E-01 |
| CENPE | PPP2R1A | 1.94E-01 | 2.45E-01 | 4.18E-01 | 2.20E-02 | 7.35E-02 | 2.14E-01 | 1.98E-01 | 7.52E-02 | 1.87E-01 | 1.00E+00 |
| CENPE | PPP2CB | 1.00E+00 | 6.51E-01 | 2.69E-01 | 1.00E+00 | 3.06E-01 | 9.04E-01 | 1.85E-01 | 5.02E-01 | 9.94E-01 | 6.61E-01 |
| FZR1 | PPP2R5D | 2.43E-01 | 3.11E-01 | 8.35E-01 | 2.42E-01 | 5.17E-01 | 7.85E-01 | 2.38E-01 | 1.13E-01 | 8.40E-01 | 5.12E-01 |
| FZR1 | PPP2R5B | 1.00E+00 | 5.72E-01 | 1.00E+00 | 3.12E-01 | 6.43E-01 | 7.40E-01 | 8.89E-01 | 9.79E-01 | 1.09E-01 | 1.00E+00 |
| FZR1 | PPP2R3B | 2.23E-01 | 5.38E-01 | 6.68E-01 | 1.34E-01 | 1.14E-01 | 7.32E-01 | 2.97E-01 | 9.98E-01 | 3.20E-01 | 6.24E-01 |
| FZR1 | PPP2R2D | 9.82E-01 | 1.64E-01 | 3.67E-02 | 6.32E-01 | 6.64E-01 | 8.43E-01 | 8.56E-01 | 1.12E-01 | 9.77E-01 | 9.98E-01 |
| FZR1 | PPP2R1A | 7.39E-01 | 1.47E-01 | 4.77E-01 | 2.86E-02 | 4.18E-01 | 1.00E+00 | 4.12E-01 | 3.87E-01 | 9.83E-01 | 1.90E-02 |
| FZR1 | PPP2CB | 1.00E+00 | 4.89E-01 | 3.06E-01 | 1.33E-01 | 2.94E-01 | 9.24E-01 | 6.89E-01 | 4.15E-01 | 9.83E-01 | 6.49E-01 |
| KNTC1 | PPP2R5D | 5.62E-01 | 5.22E-01 | 3.32E-01 | 7.15E-01 | 8.46E-01 | 3.49E-01 | 7.85E-01 | 6.54E-01 | 3.29E-01 | 5.63E-01 |
| KNTC1 | PPP2R5B | 1.00E+00 | 3.21E-01 | 1.81E-01 | 7.61E-01 | 2.98E-01 | 9.06E-01 | 8.73E-01 | 7.99E-01 | 6.26E-02 | 1.00E+00 |
| KNTC1 | PPP2R3B | 7.27E-01 | 7.12E-01 | 7.52E-01 | 9.84E-02 | 1.25E-01 | 6.20E-01 | 5.94E-01 | 9.13E-01 | 1.27E-04 | 6.15E-01 |
| KNTC1 | PPP2R2D | 6.35E-01 | 3.01E-01 | 2.21E-02 | 1.68E-01 | 6.54E-01 | 6.81E-01 | 8.40E-02 | 4.56E-01 | 5.52E-01 | 9.99E-01 |
| KNTC1 | PPP2R1A | 5.52E-01 | 2.41E-02 | 5.54E-01 | 5.24E-03 | 1.06E-01 | 1.79E-01 | 3.44E-01 | 3.79E-01 | 4.99E-01 | 1.76E-01 |
| KNTC1 | PPP2CB | 2.96E-01 | 8.18E-01 | 4.74E-01 | 1.00E+00 | 9.10E-01 | 7.71E-01 | 1.03E-01 | 1.00E+00 | 9.96E-01 | 6.98E-01 |
| MAD1L1 | PPP2R5D | 9.06E-01 | 6.15E-01 | 9.60E-01 | 1.77E-01 | 8.34E-01 | 6.82E-01 | 6.15E-01 | 3.06E-01 | 1.93E-01 | 2.92E-01 |
| MAD1L1 | PPP2R5B | 1.00E+00 | 6.35E-01 | 4.15E-01 | 8.50E-02 | 5.95E-01 | 2.81E-01 | 2.83E-01 | 9.53E-01 | 1.00E+00 | 1.00E+00 |
| MAD1L1 | PPP2R3B | 7.40E-01 | 5.83E-01 | 6.10E-01 | 5.98E-01 | 1.33E-01 | 5.93E-01 | 7.27E-01 | 9.81E-01 | 9.20E-02 | 3.43E-01 |
| MAD1L1 | PPP2R2D | 9.05E-01 | 1.54E-01 | 9.67E-02 | 6.55E-02 | 3.15E-01 | 5.92E-01 | 3.83E-01 | 7.01E-01 | 8.83E-01 | 9.37E-01 |
| MAD1L1 | PPP2R1A | 7.23E-01 | 3.87E-01 | 6.92E-01 | 1.00E+00 | 3.42E-01 | 6.13E-02 | 1.93E-01 | 4.70E-01 | 8.99E-01 | 1.00E+00 |
| MAD1L1 | PPP2CB | 1.00E+00 | 8.99E-01 | 4.93E-01 | 1.45E-01 | 2.89E-01 | 2.80E-01 | 6.30E-01 | 3.76E-01 | 9.92E-01 | 6.60E-01 |
| MAD2L1 | PPP2R5D | 5.75E-01 | 4.55E-01 | 9.37E-01 | 2.58E-01 | 7.16E-01 | 8.91E-01 | 5.49E-01 | 4.62E-01 | 1.62E-01 | 1.62E-01 |
| MAD2L1 | PPP2R5B | 8.17E-02 | 5.07E-01 | 1.33E-01 | 4.53E-02 | 2.01E-01 | 7.17E-01 | 7.27E-01 | 9.75E-01 | 3.17E-03 | 1.97E-02 |
| MAD2L1 | PPP2R3B | 2.25E-01 | 8.13E-01 | 7.22E-01 | 2.69E-01 | 8.10E-02 | 5.29E-01 | 7.97E-01 | 1.00E+00 | 5.91E-04 | 4.23E-02 |
| MAD2L1 | PPP2R2D | 6.73E-01 | 5.76E-01 | 6.63E-02 | 5.21E-01 | 5.10E-01 | 1.74E-01 | 7.13E-01 | 3.43E-01 | 6.11E-01 | 9.39E-01 |
| MAD2L1 | PPP2R1A | 3.60E-01 | 1.58E-01 | 3.01E-01 | 9.93E-03 | 2.02E-01 | 5.21E-03 | 7.68E-02 | 3.41E-01 | 3.78E-01 | 1.00E+00 |
| MAD2L1 | PPP2CB | 1.00E+00 | 2.82E-01 | 8.69E-01 | 1.00E+00 | 9.26E-01 | 8.07E-01 | 3.99E-01 | 1.60E-01 | 9.91E-01 | 9.52E-01 |
| NDC80 | PPP2R5D | 2.44E-01 | 3.08E-01 | 9.58E-01 | 5.00E-01 | 5.25E-01 | 8.40E-01 | 6.44E-01 | 6.22E-01 | 5.83E-01 | 1.69E-01 |
| NDC80 | PPP2R5B | 1.00E+00 | 3.08E-01 | 3.08E-02 | 6.71E-02 | 1.01E-01 | 9.68E-01 | 7.49E-02 | 9.58E-01 | 3.30E-03 | 1.62E-02 |
| NDC80 | PPP2R3B | 2.08E-01 | 2.89E-01 | 4.20E-01 | 1.67E-01 | 1.24E-01 | 7.24E-01 | 4.87E-01 | 9.98E-01 | 6.51E-06 | 9.91E-02 |
| NDC80 | PPP2R2D | 7.08E-01 | 5.65E-01 | 1.16E-01 | 2.28E-01 | 4.95E-01 | 7.66E-01 | 3.92E-01 | 7.43E-01 | 2.09E-01 | 9.57E-01 |
| NDC80 | PPP2R1A | 9.71E-02 | 2.35E-01 | 7.15E-01 | 3.82E-02 | 9.03E-02 | 1.00E+00 | 5.01E-01 | 6.05E-01 | 3.23E-01 | 1.00E+00 |
| NDC80 | PPP2CB | 1.00E+00 | 4.47E-01 | 1.00E+00 | 1.00E+00 | 9.29E-01 | 7.84E-01 | 4.92E-01 | 1.42E-01 | 9.95E-01 | 4.76E-01 |
| PLK1 | PPP2R5D | 6.05E-01 | 5.78E-01 | 9.42E-01 | 2.76E-01 | 4.82E-01 | 8.03E-01 | 1.64E-01 | 6.83E-01 | 1.01E-01 | 2.95E-01 |
| PLK1 | PPP2R5B | 7.44E-02 | 3.09E-01 | 1.21E-01 | 2.91E-02 | 3.22E-01 | 9.51E-01 | 2.17E-01 | 9.59E-01 | 4.17E-02 | 7.59E-03 |
| PLK1 | PPP2R3B | 6.05E-01 | 9.34E-01 | 2.38E-01 | 2.61E-01 | 7.31E-02 | 6.24E-01 | 8.36E-01 | 9.76E-01 | 1.18E-02 | 1.42E-01 |
| PLK1 | PPP2R2D | 8.68E-01 | 6.62E-01 | 6.86E-02 | 4.17E-01 | 4.84E-01 | 6.00E-01 | 7.67E-01 | 5.02E-01 | 4.76E-01 | 9.68E-01 |
| PLK1 | PPP2R1A | 3.16E-01 | 2.16E-01 | 7.30E-02 | 3.94E-02 | 2.93E-01 | 1.00E+00 | 1.50E-01 | 5.64E-01 | 4.58E-01 | 1.00E+00 |
| PLK1 | PPP2CB | 1.00E+00 | 3.52E-01 | 1.00E+00 | 1.00E+00 | 3.26E-01 | 9.64E-01 | 3.29E-01 | 1.00E+00 | 9.62E-01 | 5.71E-01 |
| PLK4 | PPP2R5D | 3.91E-01 | 6.17E-01 | 7.37E-01 | 3.92E-01 | 5.67E-01 | 9.45E-01 | 6.83E-01 | 5.28E-01 | 5.51E-01 | 4.01E-01 |
| PLK4 | PPP2R5B | 1.13E-01 | 2.30E-01 | 1.38E-01 | 7.89E-02 | 4.60E-01 | 8.72E-01 | 6.54E-01 | 9.29E-01 | 4.30E-04 | 3.21E-02 |
| PLK4 | PPP2R3B | 3.14E-01 | 9.02E-01 | 7.78E-01 | 9.21E-02 | 1.51E-01 | 2.42E-01 | 9.64E-01 | 9.85E-01 | 2.65E-06 | 1.04E-01 |
| PLK4 | PPP2R2D | 7.23E-01 | 4.37E-01 | 1.10E-02 | 2.20E-01 | 9.77E-02 | 1.05E-01 | 6.70E-01 | 3.53E-01 | 1.74E-01 | 9.54E-01 |
| PLK4 | PPP2R1A | 2.74E-01 | 2.37E-01 | 1.91E-01 | 2.49E-03 | 3.26E-01 | 1.00E+00 | 2.27E-01 | 1.60E-01 | 3.48E-01 | 1.48E-01 |
| PLK4 | PPP2CB | 1.00E+00 | 4.82E-01 | 1.34E-01 | 1.00E+00 | 8.86E-01 | 9.48E-01 | 3.41E-01 | 2.84E-01 | 9.97E-01 | 5.32E-01 |
| PTTG1 | PPP2R5D | 6.68E-01 | 3.30E-01 | 8.62E-01 | 3.81E-01 | 5.63E-01 | 7.74E-01 | 5.67E-01 | 7.47E-01 | 3.39E-01 | 1.57E-01 |
| PTTG1 | PPP2R5B | 1.00E+00 | 6.97E-01 | 1.43E-01 | 4.26E-03 | 5.39E-01 | 6.41E-01 | 1.09E-02 | 9.52E-01 | 9.99E-02 | 3.24E-02 |
| PTTG1 | PPP2R3B | 5.56E-01 | 6.60E-01 | 1.05E-01 | 2.72E-01 | 1.01E-01 | 9.84E-01 | 6.44E-01 | 9.99E-01 | 6.26E-03 | 4.12E-02 |
| PTTG1 | PPP2R2D | 8.84E-01 | 7.17E-01 | 1.75E-02 | 2.64E-01 | 7.14E-01 | 7.62E-01 | 2.54E-01 | 5.02E-01 | 4.53E-01 | 9.85E-01 |
| PTTG1 | PPP2R1A | 4.92E-01 | 5.38E-02 | 2.68E-01 | 7.41E-02 | 1.94E-01 | 4.17E-03 | 3.02E-01 | 5.21E-01 | 7.10E-01 | 1.01E-01 |
| PTTG1 | PPP2CB | 1.00E+00 | 7.17E-01 | 1.85E-01 | 1.00E+00 | 3.45E-01 | 8.95E-01 | 5.55E-01 | 1.00E+00 | 9.94E-01 | 8.34E-01 |
| TTK | PPP2R5D | 6.58E-01 | 3.97E-01 | 8.95E-01 | 4.24E-01 | 7.30E-01 | 8.03E-01 | 6.97E-01 | 7.76E-01 | 3.06E-01 | 2.76E-01 |
| TTK | PPP2R5B | 1.18E-01 | 5.97E-01 | 2.29E-01 | 9.62E-02 | 2.40E-01 | 8.63E-01 | 1.42E-01 | 7.30E-01 | 1.44E-02 | 6.50E-03 |
| TTK | PPP2R3B | 3.02E-01 | 8.74E-01 | 3.04E-01 | 1.45E-01 | 9.71E-02 | 1.32E-01 | 9.66E-01 | 9.45E-01 | 2.29E-03 | 5.46E-02 |
| TTK | PPP2R2D | 9.72E-01 | 6.09E-01 | 7.18E-02 | 3.94E-01 | 6.13E-01 | 5.00E-02 | 8.43E-01 | 4.33E-01 | 1.32E-01 | 9.67E-01 |
| TTK | PPP2R1A | 5.00E-01 | 1.81E-01 | 1.10E-01 | 1.35E-02 | 1.98E-01 | 1.00E+00 | 1.03E-01 | 2.87E-01 | 4.68E-01 | 1.00E+00 |
| TTK | PPP2CB | 1.00E+00 | 4.81E-01 | 3.92E-01 | 1.00E+00 | 9.26E-01 | 9.96E-01 | 3.12E-01 | 6.56E-01 | 9.83E-01 | 4.80E-01 |
| ZW10 | PPP2R5E | 8.27E-01 | 9.46E-01 | 4.02E-01 | 6.85E-01 | 4.38E-02 | 1.96E-01 | 6.92E-01 | 2.28E-01 | 1.83E-01 | 8.72E-01 |
| ZW10 | PPP2R5D | 8.71E-01 | 6.21E-01 | 3.32E-01 | 7.04E-01 | 3.14E-01 | 8.41E-01 | 5.65E-01 | 2.44E-01 | 4.21E-01 | 1.24E-01 |
| ZW10 | PPP2R5B | 2.95E-02 | 4.69E-01 | 4.00E-01 | 5.31E-01 | 3.46E-01 | 4.17E-01 | 5.24E-01 | 8.68E-01 | 2.01E-02 | 5.65E-03 |
| ZW10 | PPP2R3B | 7.79E-01 | 7.84E-01 | 3.64E-01 | 5.93E-01 | 6.24E-02 | 8.16E-01 | 8.35E-01 | 9.01E-01 | 6.54E-02 | 1.56E-01 |
| ZW10 | PPP2R2D | 8.02E-01 | 8.22E-01 | 1.74E-01 | 2.60E-01 | 1.09E-01 | 7.34E-01 | 2.37E-01 | 5.16E-01 | 8.12E-01 | 9.61E-01 |
| ZW10 | PPP2R1A | 8.54E-01 | 3.02E-01 | 9.12E-02 | 1.83E-01 | 2.06E-01 | 2.13E-02 | 4.40E-01 | 2.87E-01 | 1.85E-01 | 1.00E+00 |
| ZW10 | PPP2CB | 2.32E-01 | 4.21E-01 | 1.00E+00 | 2.27E-02 | 2.38E-01 | 5.87E-01 | 8.49E-02 | 4.04E-01 | 3.98E-01 | 6.78E-01 |
| ZWILCH | PPP2R5D | 2.88E-01 | 7.36E-01 | 8.48E-01 | 9.23E-02 | 5.85E-01 | 2.27E-01 | 7.01E-01 | 4.22E-01 | 2.29E-01 | 1.24E-01 |
| ZWILCH | PPP2R5B | 1.00E+00 | 6.22E-01 | 8.93E-02 | 1.87E-01 | 4.94E-01 | 9.60E-01 | 5.95E-01 | 7.78E-01 | 1.00E+00 | 1.00E+00 |
| ZWILCH | PPP2R3B | 4.22E-01 | 8.92E-01 | 2.72E-01 | 6.22E-02 | 5.63E-02 | 9.17E-01 | 2.93E-01 | 9.95E-01 | 3.90E-02 | 5.16E-02 |
| ZWILCH | PPP2R2D | 7.85E-01 | 6.08E-01 | 7.63E-02 | 3.00E-01 | 7.52E-01 | 6.34E-01 | 7.29E-01 | 1.00E+00 | 9.96E-01 | 8.96E-01 |
| ZWILCH | PPP2R1A | 3.98E-01 | 5.66E-02 | 2.11E-01 | 7.48E-03 | 6.27E-02 | 1.06E-01 | 1.67E-02 | 4.18E-01 | 8.81E-01 | 5.42E-02 |
| ZWILCH | PPP2CB | 6.05E-01 | 2.95E-01 | 2.61E-01 | 1.00E+00 | 3.61E-01 | 9.89E-01 | 5.58E-01 | 6.30E-02 | 9.99E-01 | 8.54E-01 |
